# Navigating risk: A holistic framework for supporting rural livelihoods adaptation in Solomon Islands

**DOI:** 10.1007/s13280-025-02162-4

**Published:** 2025-03-28

**Authors:** Bethany Rose Smith, Hensllyn Boseto, Aubrey Vavu, Relna Peter, Stephanie Duce, Amy Diedrich

**Affiliations:** 1 College of Science and Engineering, James Cook University, Bebegu Yumba Campus, 1 James Cook Drive, Douglas, Townsville, QLD Australia; 2 Centre for Sustainable Tropical Fisheries and Aquaculture, College of Science and Engineering, James Cook University, Townsville, QLD Australia; 3 Ecological Solutions Solomon Islands, PO Box 180, Gizo, Western Province Solomon Islands

**Keywords:** Community adaptation planning, Multi-disciplinary perspective, Risk assessment, Rural livelihoods, Solomon Islands, Vulnerability assessment

## Abstract

This paper presents the Livelihoods-Based Risk Profiling Framework (LRPF) to support rural livelihoods adaptation in Western Province, Solomon Islands. Our framework addresses narrow disciplinary perspectives to provide a more comprehensive understanding of risks to rural livelihoods. We expand previous climate-focused frameworks by incorporating non-climatic hazards and key socio-economic factors that influence vulnerability. We employed a participatory mixed-methods approach using focus group workshops and household surveys to assess risk across three rural communities. The data were analysed to produce decision support tools including livelihood risk profiles, vulnerability index tables, and risk interaction diagrams. These tools identified priority adaptation initiatives targeting key risk drivers, demonstrating the LRPF’s capacity to support communities in addressing complex risks and aiding external stakeholders to better align their interventions with local realities. By adopting a holistic perspective, the LRPF can enhance the robustness of adaptation planning, leading to more effective and sustainable adaptation outcomes for rural livelihoods.

**Supplementary Information:**

The online version contains supplementary material available at 10.1007/s13280-025-02162-4.

## Introduction

As climate-change impacts proliferate, a growing body of research has focused on understanding threats to rural livelihoods (Msimanga and Mukwada 2022), comprising the capabilities, assets, and activities required for a means of living (DFID 1999). Community-based adaptation planning (from here on referred to as adaptation planning) plays a crucial role in addressing these threats (Barrowman and Butler 2020). Specifically, adaptation planning engages communities in identifying potential climate-change impacts and developing strategies to reduce their likelihood (Butler et al. 2016). This participatory approach promotes adaptation that is tailored to local contexts and priorities, thereby increasing its long-term effectiveness (Werners et al. 2021).

To date, the concept of vulnerability has been widely applied to support adaptation planning (Kim et al. 2021). At the local scale, community-based vulnerability assessments (CBVAs) provide a framework for understanding and analysing local sensitivity and capacity for adaptation to the impacts of climate change (Adger 2006). However, climate-change CBVAs have been limited by narrow perspectives that fail to consider the full range of factors contributing to vulnerability (Smith and Diedrich 2024). This research problem specifically includes: (i) a focus on climate change as the main threat to livelihoods, (ii) limited consideration of local socio-economic characteristics, and (iii) failure to address spatial and temporal dynamics (McDowell et al. 2016; Jurgilevich et al. 2017; Ford et al. 2018). These limitations have caused CBVAs to inform ineffective or even harmful adaptation for the communities they aim to support (Ford et al. 2018).

Climate-change CBVAs commonly apply (Smith and Diedrich 2024) the Intergovernmental Panel on Climate Change (IPCC) AR4 Vulnerability Framework (IPCC 2007). However, in 2014 the IPCC transitioned from assessing vulnerability to climate change, to evaluating the risks posed by climate change, captured in their AR5 Risk Framework (IPCC 2014). This transition aimed to address the narrow perspectives of the AR4 by incorporating holistic concepts from the disaster risk reduction discipline (Reisinger et al. 2016). Unlike CBVAs, community-based risk assessments (CBRAs) aim to predict potential livelihood impacts by comprehensively evaluating locally relevant multi-hazard exposures and the underlying socio-economic characteristics that influence the likelihood these impacts will occur (Gero et al. 2010). Given this holistic perspective, risk is increasingly viewed as a more robust concept for supporting adaptation planning (Van Aalst et al. 2008; Marin-Puig et al. 2022). However, the AR5 has not yet been applied to rural livelihoods contexts (Smith and Diedrich 2024), and a lack of clear guidelines for its implementation masks potential benefits (Ishtiaque et al. 2022).

CBRAs may be particularly valuable in remote countries, such as Solomon Islands, which are at high risk of impact from climate change (Msimanga and Mukwada 2022). This heightened risk is partly due to local dependence on the natural environment to meet livelihood security objectives (LSOs) (i.e. the ability of a household to meet basic needs) (Frankenberger and McCaston 1998), including access to food, water, housing, energy, and income (SPC 2016). This dependence increases local exposure to climate change, as livelihoods rely heavily on ecosystem services susceptible to hazards like sea-level rise, extreme weather, and changes in temperature and rainfall patterns (Marshall et al. 2013). Recognising these risks, Solomon Islands has prioritised national efforts towards adaptation planning (Solomon Islands Ministry of Environment 2023). However, many of these efforts overlook the impacts of non-climatic hazards on rural livelihoods (McNamara et al. 2020; Minter and Van der Ploeg 2023).

The primary research objective of this paper is to support adaptation planning efforts by developing and implementing the Livelihoods-Based Risk Profiling Framework (LRPF) which applies and expands the AR5 (IPCC 2014) to rural livelihoods. Our framework specifically addresses the limitations of CBVAs to illustrate how a holistic risk-based approach can achieve more robust outcomes for adaptation planning. To achieve this, we incorporate both climatic and non-climatic threats to rural livelihoods, along with a thorough analysis of the socio-economic factors that contribute to vulnerability. We note that while the LRPF addresses rural livelihoods risk, its outputs are designed to support communities in meeting their basic needs, rather than providing adaptation insights for specific livelihood activities like farming or fishing.

In this paper, we introduce the LRPF and provide a detailed step-by-step method for its implementation. We implement the LRPF in three rural communities in Western Province, Solomon Islands and produce a range of decision support tools for each community that summarise risk. We interpret these tools to demonstrate how the LRPF’s holistic perspective can provide more robust guidance for adaptation planning. We use these tools to identify priority adaptation initiatives for each community and describe the underlying risk drivers behind each initiative. At a broader scale, we explore how a holistic perspective on risk can help external stakeholders align their support for adaptation efforts with local realities. Through these interpretations, we reflect on how the LRPF can support more effective and sustainable adaptation outcomes for rural livelihoods.

## Materials and methods

This study employed a mixed-methods research design to assess community-level risks to rural livelihoods in Solomon Islands using the Livelihoods-Based Risk Profiling Framework (LRPF). Below, we outline this framework, with a specific focus on expansions made to the AR5, before detailing its implementation. The LRPF assesses risk by combining quantitative and qualitative data from focus group workshops, household surveys, and informal conversations. This mixed-methods design facilitates the creation of decision support tools that assist in selecting priority adaptation initiatives for rural livelihoods.

### Livelihoods-Based Risk Profiling Framework

**Fig. 1:**
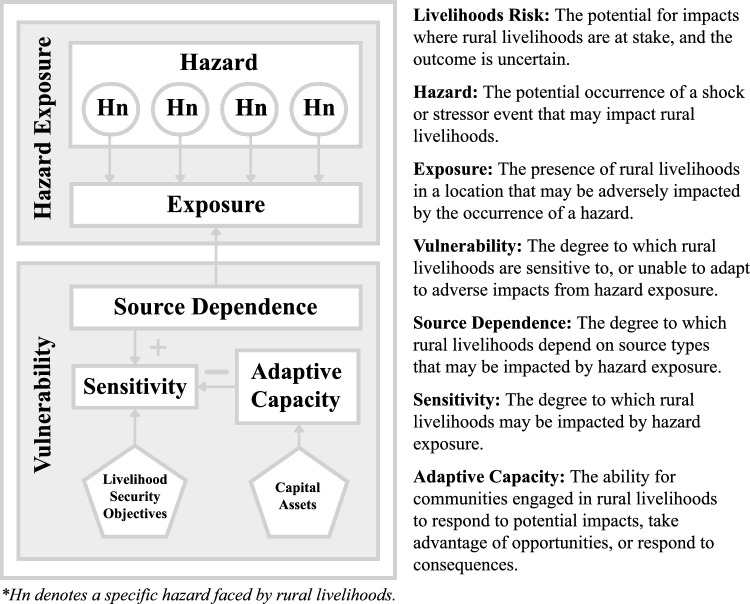
The Livelihoods-Based Risk Profiling Framework (LRPF) and associated definitions of the components that define risk

The LRPF is an expanded version of the AR5 Risk Framework (IPCC 2014) that is oriented towards understanding community-level risk in the context of rural livelihoods (Fig. 1). In the LRPF, risk represents the ‘potential for impacts where rural livelihoods are at stake and the outcome is uncertain’. Risk in the AR5 is comprised of hazard exposure and vulnerability, and vulnerability is comprised of sensitivity and adaptive capacity. We adapted the AR5 definitions of these components of risk to fit the rural livelihood’s context. This included adopting a holistic approach to hazards, that encompassed both climatic and non-climatic factors. Additional modifications to the AR5 included expansion of the types of socio-economic factors that contribute to vulnerability. Specifically, we introduced a series of composite indicators of livelihood security objectives (LSOs) (i.e. water, food, housing, energy, and income insecurity) to assess sensitivity (SPC 2016) and incorporated composite indicators of capital assets into the adaptive capacity component (i.e. human, financial, social, physical, and natural capital) (DFID 1999).

We also introduced source dependence as an additional component of vulnerability, encompassing the natural or modified ecosystems, as well as artificial infrastructure, that communities rely on to meet their LSOs. We considered source dependence to influence sensitivity to hazard exposure. For example, communities that depend on coral reef fisheries for food security may be more sensitive to climate-change impacts on these fisheries than communities that depend on a diversity of food sources. By incorporating source dependence, the LRPF establishes a direct link between exposure to a specific hazard and vulnerability (Filho et al. 2020; Kim et al. 2021).

We developed an index to assess the vulnerability component of livelihoods risk using a systematic review of adaptive capacity and sensitivity indicators applied in prior CBVAs (Smith and Diedrich 2024). These indicators were refined to the local context via a consultative process with local experts. Table 1 presents the final list of adaptive capacity (n = 19) and sensitivity (n = 10) indicators included in the vulnerability index and their definitions. These indicators reflect the socio-economic characteristics underlying a community’s vulnerability to various hazards. Further details on each indicator’s origins, adaptations, and contributions to vulnerability can be found in the supplementary information.

**Table 1:** The underlying components, composite indicators, and indicators comprising the LRPF vulnerability index. Weighting values (W%) represent the values assigned to aggregate these elements into an overall vulnerability index score. Further description on each indicator, their contribution to vulnerability, and the data collection and analysis methods are provided in the supplementary information

Indicator	Definition	W%
Adaptive capacity	The ability for communities engaged in rural livelihoods to respond to potential impacts, take advantage of opportunities, or respond to consequences	**50**
Human Capital	The skills, knowledge, ability to labour and good health that together enable people to pursue different livelihood strategies and achieve their livelihood objectives	**8**
HC1: Dependency Ratio	The ratio of dependent (i.e. non-working adults) to non-dependent household members (i.e. working adults)	6.7
HC2: Health Condition	The number of times in the last year household members missed their livelihood responsibilities due to being sick, ill or injured	6.5
HC3: Access to Healthcare	The amount of time a household can access healthcare in times of need	10.1
HC4: Livelihood Diversity Index	The number of unique livelihood activities that members of a household are engaged in	23.3
HC5: Extent of Coping Strategies	The extent to which a household believes they can cope with locally relevant hazard exposure	53.4
Financial Capital	The financial resources that people use to achieve their livelihood objectives	**24.7**
FC1: Household Savings	The total amount of savings accumulated by a household	45
FC2: Household Income	The amount of income a household acquires in a one-month period	16.7
FC3: Household Expenditure	Household monthly expenditure on basic needs (e.g. food, water, housing, energy and healthcare)	9.8
FC4: Income Satisfaction	The extent to which a household is satisfied with their ability to purchase basic goods	2.7
FC5: Access to Financial Services	Household access to key financial services including bank accounts, pensions, and loans	25.8
Social Capital	The social resources upon which people draw in pursuit of their livelihood objectives	**13.6**
SC1: Social Networks	The number of social relationships a household can turn to for support in times of need	13.2
SC2: Inclusion in Decision Making	The extent of household satisfaction with their inclusion in community decision making	9.8
SC3: Local Institutional Memberships	Household membership in local institutions (e.g. groups, organisations, and associations)	6.4
SC4: Satisfaction with Leadership	The extent to which a household is satisfied with the leadership in their community	3
SC5: Trust	The level of trust a household has in community members and leaders	14.6
SC6: Collective Action	The extent to which a household participates in community-based activities	22.1
SC7: Perceptions of Fair Access to Livelihood Opportunities	Household perception of fair access to livelihood opportunities within their community	30.9
Physical Capital	The basic infrastructure and goods needed to support livelihoods	**7.8**
PC1: Access to Livelihoods-Based Assets	Household access to key assets required to support livelihoods	100
Natural Capital	Fair access of households to stocks of natural resources that are important for livelihoods	**45.9**
NC1: Perceptions of Fair Access to Natural Resources	Household perception of fair access to natural resources within their community	100
Sensitivity	The degree to which rural livelihoods may be impacted by hazard exposure	**50**
Water Security	Access to adequate quantities of acceptable quality water for sustaining livelihoods and ensuring protection against water borne diseases	**52.8**
WS1: Access to Drinking Water	Household has access to improved sources of drinking water within a 30 minute return distance	10.8
WS2: Water Sufficiency	Household has access to sufficient quantities of water to meet basic needs	38.9
WS3: Water Quality	The extent to which a household is satisfied with the quality of their drinking water	42.7
WS4: Access to Sanitation	Household has access to an improved form of sanitation	7.6
Food Security	Access to sufficient, safe, and nutritious foods to maintain a healthy and active lifestyle	**25.1**
FS1: Food Sufficiency	Household has uninterrupted access to sufficient volumes of food to meet household requirements	80
FS2: Food Consumption Score	The frequency of consumption of different food groups during the previous 7 days	20
Housing Security	Access to adequate housing	5.7
HS1: Housing Condition	The type of material used for the roof, flooring, and walls of a household’s shelter	100
Energy Security	Access to uninterrupted energy sources	4
ES1: Cooking Sufficiency	The amount of time a household has problems accessing cooking fuel	87.5
ES2: Lighting Sufficiency	The amount of time a household has problems accessing lighting	12.5
Income Security	Access to reliable and stable income that can meet basic needs	12.4
IS1: Income Stability	The extent to which household income has fluctuated over the previous 12 months	100

While source dependence contributes to a community’s overall sensitivity, it was not included as a component of the vulnerability index (Fig. 1). This decision reflects the ‘generalised’ perspective of adaptive capacity and sensitivity indicators, which encompass socio-economic characteristics influenced by various hazard types. In contrast, source dependence was treated as a hazard specific component of vulnerability, directly tied to specific hazard types. By omitting source dependence from the vulnerability index, we aimed to clearly distinguish between general vulnerability factors and those specific to certain hazards.

### Study communities

**Fig. 2:**
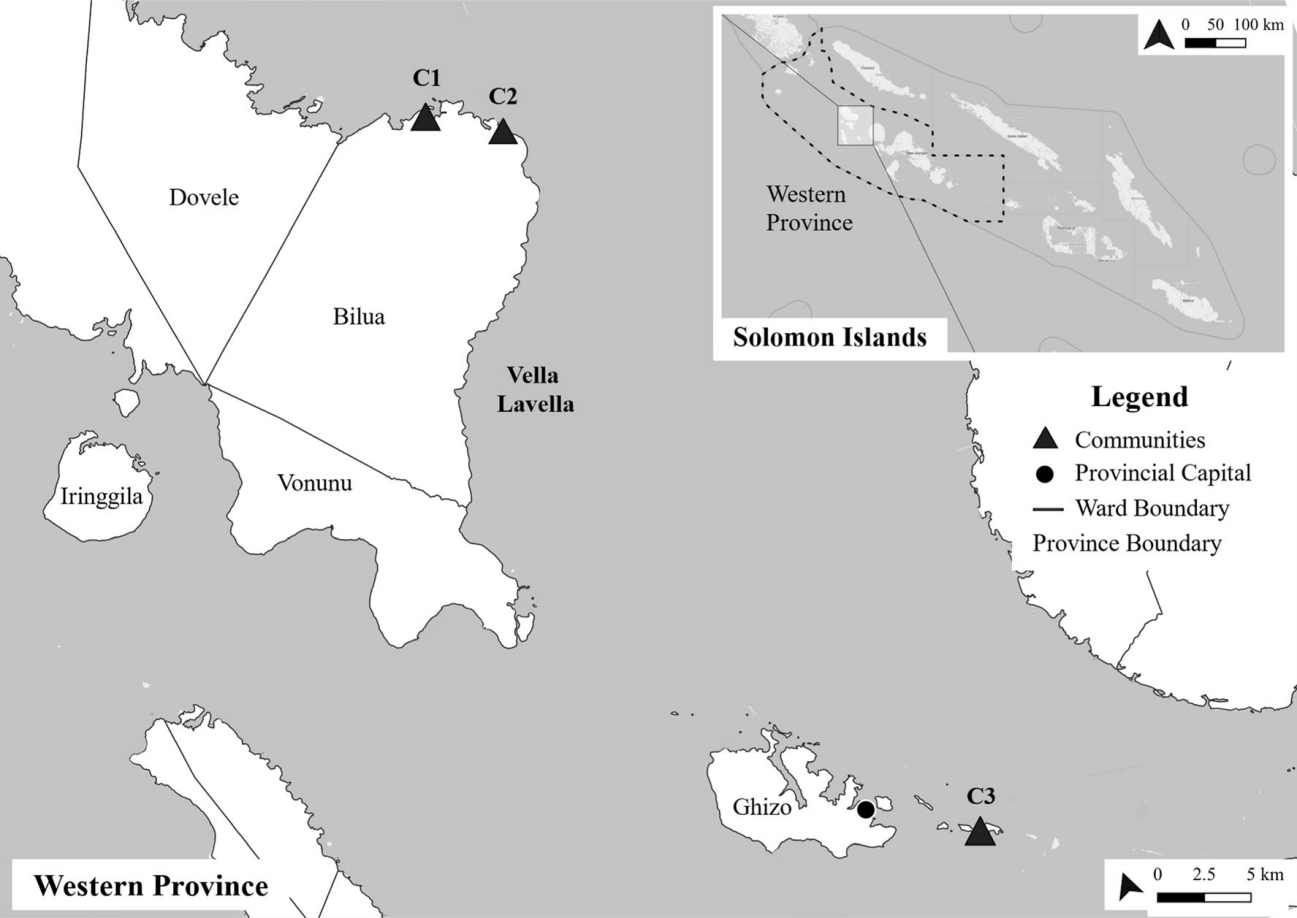
The location of LRPF study communities in Western Province, Solomon Islands

The LRPF was implemented in three rural communities in Western Province, Solomon Islands. Two were in Bilua Ward on the island of Vella La Vella (C1 and C2) and one in Ghizo Ward (C3) on an island approximately 5km from the provincial capital of Gizo (Fig. 2). Communities were selected based on their rural location, high dependence on natural resources for livelihoods, and having an interest in participating in community-based adaptation planning. Communities differed in size, comprising 150 (C1), 141 (C2), and 50 (C3) households. Community livelihoods were primarily subsistence, centring around gardening and selling produce at market in C1, gardening and cash crop farming in C2, and coastal fishing in C3. For all communities, access to basic utilities was limited, with households reliant on locally sourced natural resources to access water, food, building materials, cooking fuel, and income (Table 2). The remoteness of the communities limited their access to facilities such as healthcare centres, markets, shops, and banks, located in Gizo town.

**Table 2:** Demographic characteristics of survey respondents across the three study communities

Characteristic	C1	C2	C3
Number of households interviewed	63	76	50
Percentage of households interviewed	42	54	100
Percentage of male respondents	43	41	54
Age of respondent			
20–29 years old (%)	19	13	18
30–39 years old (%)	25	25	42
40–49 years old (%)	22	22	12
50–59 years old (%)	24	21	8
Over 60 years old (%)	10	19	20
Size of Household			
1–3 household members (%)	8	9	22
4–6 household members (%)	46	58	42
7–9 household members (%)	32	30	28
10 or more household members (%)	14	3	8
Percentage of non-resident households^a^	17	16	22
Subsistence level^b^	2.06	1.96	2.78
Standard Deviation	0.66	0.59	1.03
Primary livelihood activity			
Food gardening (%)	48	43	14
Fishing (%)	3	5	56
Cash crop farming (%)	5	38	0
Market seller (%)	21	4	10
Use of local ecosystems for access to essential needs			
Water (%)	100	100	100
Food (%)	98	100	54
Housing materials^c^ (%)	60	65	37
Cooking fuel (%)	100	100	98
Lighting (%)	0	0	0
Income (%)	92	91	84

^a^Non-resident households did not originally reside in the community

^b^Five-point ordinal scale representing the % of food that a household produces or catches that is sold at market (5 represents where 100% of food is sold at market)

^c^Presented as average dependence across roofing, walls, and flooring materials

### Data collection

Unlike the AR5, the LRPF does not assign a single empirical value to risk, but summarises quantitative and qualitative data through a range of decision support tools. We designed a mixed-methods approach to gather the data underlying the LRPF, using established methods for assessing community-level risk and vulnerability (Smith and Diedrich 2024). Qualitative and spatial data were collected through focus group mapping workshops to gain a detailed understanding of a community’s experiences with hazard exposure. While a household-level survey was conducted to gather data on vulnerability. This collected quantifiable information on source dependence, adaptive capacity, and sensitivity, as well as household-level hazard exposure, enriching the data obtained from the focus group workshops. Finally, qualitative data from informal conversations were incorporated to further contextualise risk.

Data collection occurred from March to September 2022 under James Cook University’s human ethics protocol H8497 ensuring adherence to all relevant ethical considerations, including informed consent and confidentiality. All research activities were facilitated by local researchers and conducted in Solomon Island Pijin and Bilua, the Vella La Vella dialect.

#### Focus group mapping workshops

Focus group mapping workshops were conducted to elicit local perceptions on the main types of hazard that communities were exposed to. Participants were selected with community leaders’ support to ensure representation across the community’s demographic and spatial extent. The workshop included a minimum of twelve individuals, comprising six males and females, four youth, adults, and elders, and representatives from a range of livelihood activities. Workshops were held in a community gathering space, with groups sex-disaggregated based on local advice.

During the first workshop activity, participants received cards representing eight hazards identified by local experts to impact rural livelihoods in Western Province. These hazards included commercial logging, coastal fishing, sea-level rise, flooding, increased rainfall, decreased rainfall, temperature increase, and COVID-19. In this context, coastal fishing was viewed as both a livelihood activity and a hazard due to declines in wild fish availability linked to overexploitation (Gillet and Tauti 2018). Participants received blank cards to list additional hazards impacting local livelihoods. Participants were then given 45 minutes to rank the cards from highest to lowest concern based on their perceived livelihood impact. Hazards deemed irrelevant by the community were excluded from the ranking process. Consensus ranking scores for male and female groups were reached through facilitated discussions and negotiations.

Following the ranking exercise, participants engaged in two mapping activities, each lasting about an hour. Using coloured pens, participants marked up Bing or Google Earth satellite images (depending on image quality) of a community’s land and marine areas, printed and laminated onto A0-sized posters. The extent of these areas was confirmed by community leaders during preliminary site visits in March 2022. Before mapping commenced, local researchers oriented participants by highlighting key landmarks on the images. Participants then drew the locations of the top-ranking hazards from the previous activity. Only hazards exhibiting spatial variation at the community scale, such as sea-level rise, were mapped, excluding hazards with broader-scale variations, like temperature change. Upon completion, we photographed the marked images for digitisation before wiping them clean for the second mapping activity.

In the second activity, participants were asked to draw the locations of resources used to meet their LSOs, such as wells for water security, or mangroves for housing security. They marked these locations on the satellite images using polygons or points and added notes identifying each source type and its LSO purpose. To facilitate this activity, participants were also provided with A3 posters of Western Province, acknowledging that some resources, like markets, may exist outside the community. Marked-up images representing LSO source locations were photographed upon completion.

#### Household surveys

Quantitative data on hazard exposure and vulnerability (i.e. sensitivity, adaptive capacity, and source dependence) were obtained from household surveys. Survey questions, provided in the supplementary information, were comprised of Likert-scale, open-ended, and categorical question types. The survey was translated into the local language, Pijin, by the local research team using a back-translation method. It was then piloted with Pijin speaking participants in the provincial capital of Gizo, who were randomly selected from people encountered on the street.

Convenience sampling was used to select households within each community. Community leaders assisted in accessing a diverse representation of households throughout a community’s geographic area. Due to time constraints, a minimum quota of 40% of households was established. This resulted in a sample size of 63 (C1), 76 (C2), and 50 (C3) representing 42%, 54%, and 100% of households respectively. Local researchers conducted the surveys via face-to-face interviews at participant’s homes in either Pijin or Bilua. Each survey session lasted approximately 40 minutes. Households were initially approached during the daytime. If this timing was inconvenient, local representatives, who helped researchers engage households, arranged to return at a more suitable time. Surveys alternated as much as possible between male and female household heads to provide a balanced representation of responses across sexes (Table 2).

### Data analysis

#### Hazard exposure

Analysis of the focus group hazard ranking activity was conducted to obtain a mean ranking score for each hazard, combining responses from male and female groups. We acknowledge that the focus group data were sex-disaggregated and note that differences in ranking scores were observed that would be of relevance to a risk profiling exercise that emphasised gender dimensions. However, this level of detail was outside the scope of this paper, which is intended to give an overview of the LRPF.

After obtaining aggregated ranking scores for individual hazards, we categorised them into thematic groups, including climate change, resource exploitation, environmental, and socio-economic issues. We then calculated mean scores for each theme by averaging the scores of the underlying hazards, presenting these as ‘levels of concern’ ranging from very low to very high. These levels reflected the perceived impact of each hazard on rural livelihoods.

The hand-drawn maps were digitised using QGIS 3.36.0 to create individual vector polygons. These polygons depicted: (i) the spatial distribution of high concern hazards exhibiting spatial variation at the community scale and ii) the locations and descriptions of source types used for LSOs. Source types were categorised as natural ecosystems (e.g. oceans and forests), modified ecosystems (e.g. food gardens and cash crop plantations), and artificial infrastructure (e.g. rainwater tanks). Hazard polygons were then overlayed onto source type polygons to characterise their interactions. These interactions were further defined through the analysis of qualitative data from workshops and informal conversations, offering insights into how different hazards intersect and influence one another. This contributed to a deeper understanding of risk dynamics within the local context.

Finally, we analysed open-ended questions from household surveys to further identify the hazards faced by different LSO source types, enhancing our understanding of broader-scale hazard exposures not covered in the mapping exercise. These data were summarised at the community level.

#### Vulnerability

Household survey data on LSO source dependence were analysed to identify the primary sources used for accessing water, food, housing, energy, and income. The data were summarised at the community level to establish percentage dependence on each source type, which were categorised as natural ecosystems, modified ecosystems, or artificial infrastructure.

To construct the vulnerability index, we first used raw data from household surveys to calculate the standardised indicators listed in Table 1, following the detailed methods provided in the supplementary information. A multi-criteria evaluation (MCE) analysis using the analytic hierarchy process (AHP) was employed to weight the indicators that comprised the composite indicators (i.e. LSOs and capital asset categories) and subsequently to calculate vulnerability and its components (Saaty 1980; Maanan et al. 2018). The weighting values presented in Table 1 reflect the AHP outcomes, where the research team assessed the relative importance of indicators, composite indicators, and vulnerability components through pairwise comparisons. To achieve a consensus on the weighting score among the research team the Delphi technique was used, applying a maximum consistency ratio of 10% to ensure consistent logic (Kaynak and Macaulay 1984).

Composite indicator scores were calculated in R using the baseR, tidyverse, and scales packages. The following formula was applied: *Aggregated Score* = w_1_*Score_1_ + w_2_*Score_2_ + … + w_n_*Score_n_; where the *Aggregated Score* represents a composite indicator, w_n_ represents indicator weightings, and Score_n_ represents indicator values. Composite indicators were standardised to a 0–100 scale and then aggregated to produce values for the vulnerability components (sensitivity and adaptive capacity) using the same formula. These components were also standardised. Given the inverse relationship between adaptive capacity (AC) and sensitivity (S), their aggregation to produce the final vulnerability (V) index followed the formula: V = *f*(AC-S). Household-level vulnerability index scores were standardised and combined to obtain a mean value for community vulnerability, along with its underlying components and composite indicators. These values were classified into ranks: very low (0–20), low (21–40), moderate (41–60), high (61–80), and very high (81–100), with ranks for adaptive capacity and its associated composite indicators occurring in reverse order. The vulnerability index was contextualised through qualitative data analysis, revealing interactions between indicators, LSO source dependence, and hazard exposure.

#### Risk-based decision support tools

As mentioned previously, the LRPF does not calculate a single value for risk but integrates qualitative and quantitative data on risk components (Fig. 1). This approach offers a comprehensive overview of risk to guide prioritised adaptation initiatives for rural livelihoods. We developed a suite of decision support tools that effectively capture the complex factors contributing to the LRPF. These included: livelihood risk profiles (LRPs), vulnerability index tables, and risk interaction diagrams.

The LRPs provided a baseline overview of risk by summarising data on hazard exposure and vulnerability, inclusive of the vulnerability index and source dependence. LRPs visually represented risk by displaying: (i) relevant hazards and their level of concern, (ii) the exposure of LSO sources to different hazard types, (iii) spatial interactions between hazards and LSO sources, (iv) local dependence on LSO sources, and v) vulnerability index outputs (e.g. composite indicator summaries of sensitivity and adaptive capacity). Data were presented using a traffic light scale, ranging from red (very high potential for impact) to green (very low potential for impact). The LRPs were supplemented by a vulnerability index table that reflected the contribution of individual adaptive capacity and sensitivity indicators to the overall index score. Finally, qualitative data were summarised using risk interaction diagrams adapted from Gill and Malamud’s (2016) method, to provide a contextual understanding of interactions among risk components and their underlying drivers.

Together, these decision support tools were used to identify priority adaptation initiatives that target the most critical drivers of risk within a community. By analysing the interactions among risk components, we ensured that adaptation initiatives accounted for the complexity of these relationships. For example, flooding may be recognised as a high concern hazard due to its magnitude, severity, and historical patterns of exposure to LSO sources. However, if a community has low dependence on these exposed sources to meet their LSOs, their overall vulnerability to flooding may be low. Consequently, adaptation responses to flooding may be deprioritised in favour of addressing high concern hazards that impact LSO sources vital for meeting essential needs like food or water.

## Results

**Fig. 3:**
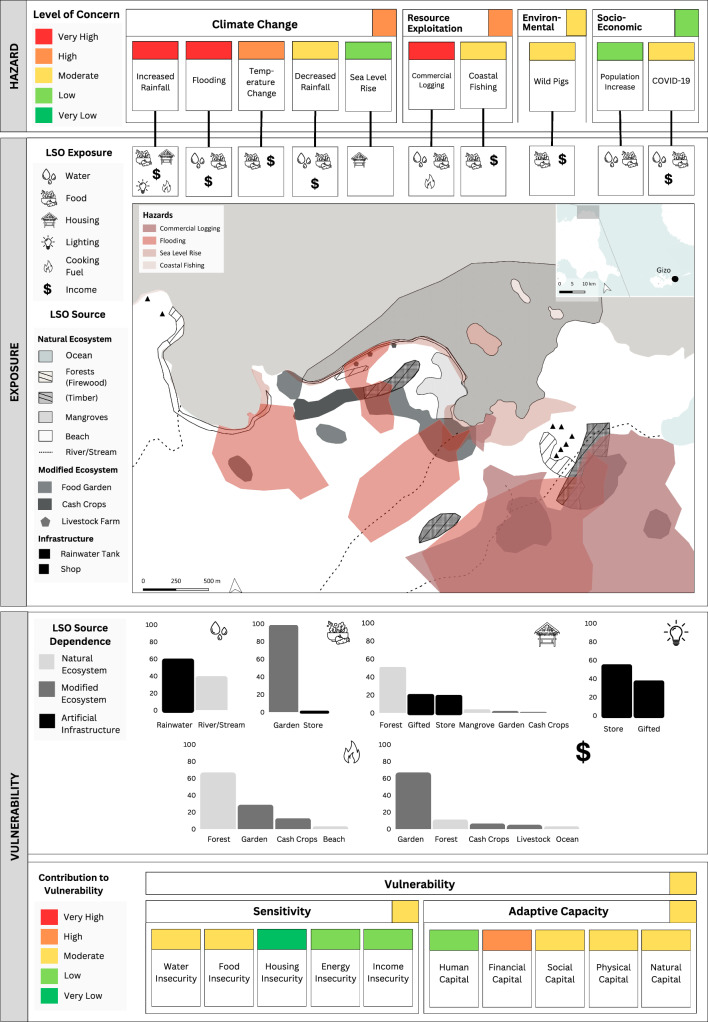
The livelihood risk profile (LRP) developed for Community 1 (C1) where livelihoods centre around food gardening and selling produce at market

**Fig. 4:**
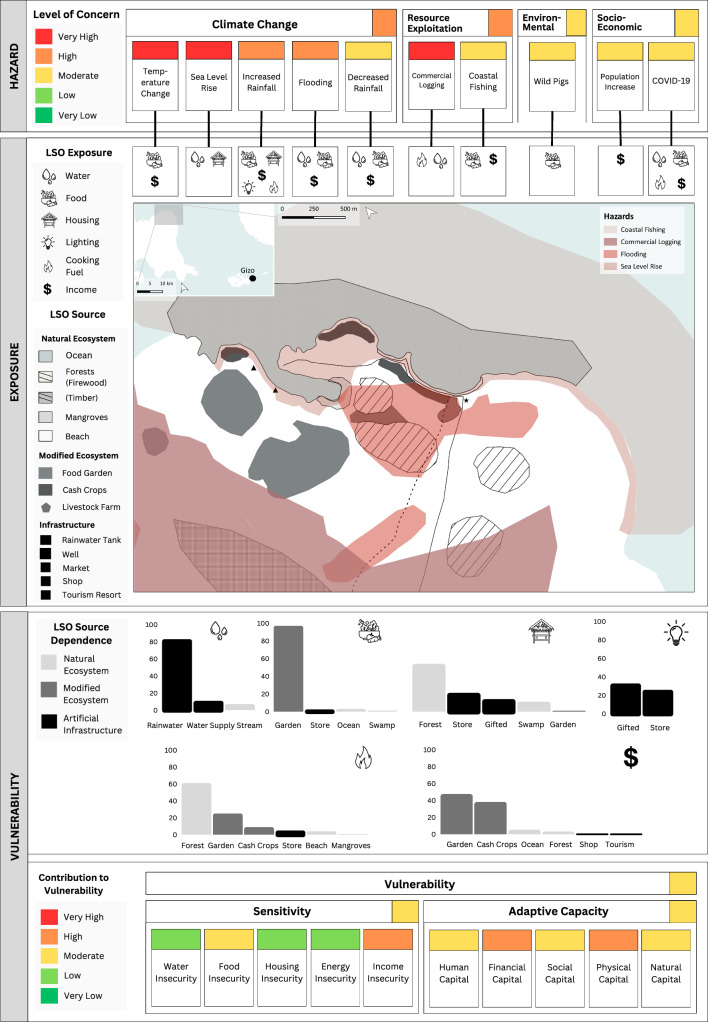
The livelihood risk profile (LRP) developed for Community 2 (C2) where livelihoods centre around food gardening and cash crop farming

**Fig. 5:**
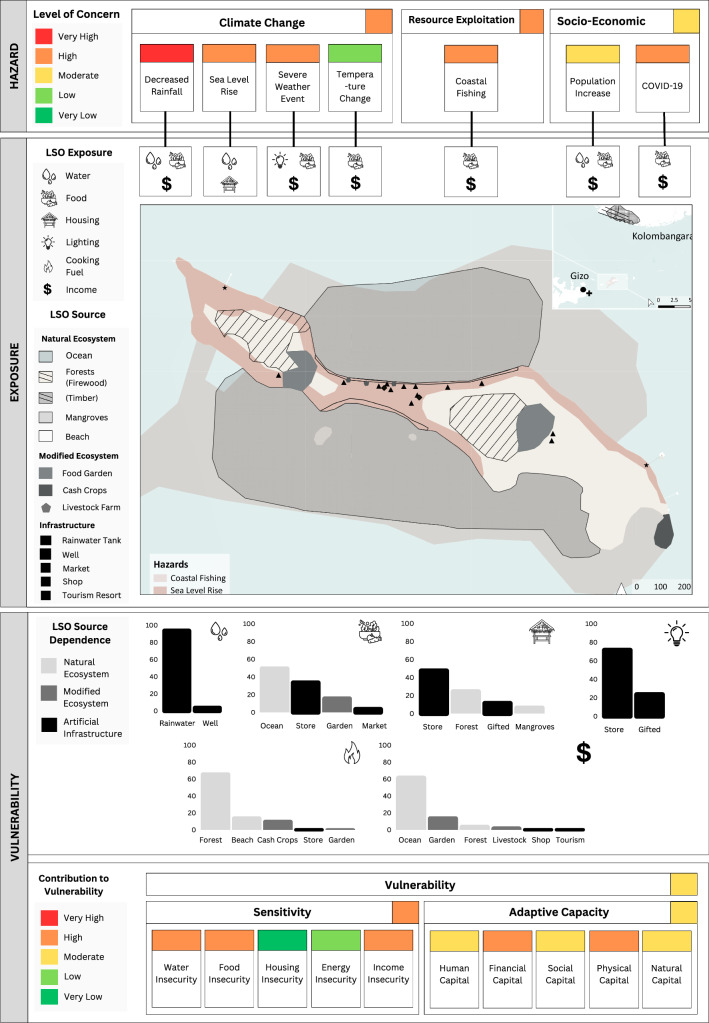
The livelihood risk profile (LRP) developed for Community 3 (C3) where livelihoods centre around coastal fishing

Our results identify a priority adaptation initiative for each community and outline the key risk factors that informed their selection. These initiatives were derived from data presented in the livelihood risk profiles (LRPs) (Figs. 3, 4, and 5), as well as the vulnerability index table and risk interaction diagrams. While all decision support tools contributed to the selection of adaptation priorities, only the LRPs are presented in this paper as they encapsulate the core outputs of the LRPF. We have provided the vulnerability index tables and risk interaction diagrams for reference in the supplementary information.

### Community 1: improved crop productivity in food gardens

Improved crop productivity in food gardens was identified as the priority adaptation initiative for C1. The C1 LRP (Fig. 3) revealed food sources to be exposed to a variety of hazards, including (i) climate-based stressors such as temperature and rainfall variability, (ii) resource exploitation including commercial logging and coastal fishing, (iii) environmental issues such as wild pigs (considered pest species), and iv) socio-economic issues including population increase and COVID-19. Exposure to climate change played a primary role in reducing the productivity of kitchen garden crops, which comprised the community’s primary source of food and income. This was further exacerbated by indirect commercial logging impacts, with habitat loss driving the translocation of wild pigs into garden areas in search of alternative food sources. Additionally, in areas where food gardens were situated adjacent to logged forest, the impacts of flooding were increased.

Widespread interactions between logging, flooding, and food garden areas were observed in hazard exposure maps. Despite reductions in garden productivity, there had been increased local dependence on this food source. This shift was driven by exposure to population increase, which intensified fishing activities and decreased wild fish availability. Additionally, market fluctuations induced by COVID-19 reduced incomes and limited local ability to purchase store-bought food. Together, these hazards impacted food insecurity by reducing food sufficiency and access to nutritionally diverse food sources. The presence of moderate levels of food insecurity, combined with a high dependence on gardens as a food source, indicates a high risk of impact in C1 if hazard-induced reductions in kitchen garden productivity persist. Furthermore, limited access to financial resources may worsen the situation, restricting C1’s ability to procure food from alternative sources such as markets or shops.

### Community 2: improved access to income generating opportunities

Improved access to income generating opportunities was identified as the priority adaptation initiative for C2. The C2 LRP (Fig. 4) showed that income sources were exposed to various hazards, including climate change, resource exploitation (i.e. coastal fishing), and socio-economic issues (i.e. population increase and COVID-19). Among these hazards, climate-based challenges were deemed to have the greatest overall impact on livelihoods. Specifically, exposure to climate change resulted in income loss from food gardens due to reduced crop productivity. This was further exacerbated by exposure to coastal fishing which impacted income by decreasing wild fish availability. In response, many households had shifted to cash crop farming as their primary income source. However, income from cash crops, such as copra and cocoa, had recently declined due to market fluctuations induced by COVID-19. Collectively, these factors contributed to high rates of income insecurity in C2. The community’s ability to access alternative income generating activities was largely restricted by their limited access to physical assets, including transport and equipment for conducting livelihood activities.

### Community 3: improved access to safe drinking water

Improved access to safe drinking water was identified as the priority adaptation initiative for C3. The C3 LRP (Fig. 5) showed that decreased rainfall was perceived as the greatest threat to livelihoods. This hazard had impacted the ability of household’s to access drinking water, which had been further exacerbated by population increase and sea-level rise. Hazard exposure maps revealed spatial interactions between the locations of drinking water sources, such as groundwater wells, and rising sea levels driving the salinisation of water. As a result, community members had shifted from using groundwater wells to sharing rainwater tanks. High rates of water insecurity were already evident within the community, with underlying indicators revealing low access to drinking water, as well as issues with sufficiency and quality. The community also faced challenges due to low rates of financial capital, which may impede their ability to invest in alternative water sources, such as purchasing additional rainwater tanks. Compounding these difficulties, household income, primarily derived from coastal fishing, had suffered due to population growth which had driven increased fishing pressure in local waters.

## Discussion

We have introduced the LRPF and implemented it in three rural Solomon Island communities to develop risk-based decision support tools. These tools were used to identify priority initiatives for livelihoods adaptation. In the discussion, we explore how expansion of the IPCC AR5 framework (IPCC 2014) contributed to a more robust understanding of risk in the LRPF. Subsequently, we discuss the LRPF’s value for guiding more effective and sustainable adaptation planning outcomes. Finally, we outline key future steps and additional analytical capabilities of the LRPF that extend beyond the scope of this paper but could further enhance its contributions.

### Evaluating expansion of the AR5 framework

The LRPF expanded the AR5 Framework to obtain a more holistic understanding of risks to rural community livelihoods. To reiterate, this included: (i) incorporating non-climatic hazards, (ii) expanding socio-economic vulnerability indicators, and (iii) introducing source dependence. In the following subsections, we evaluate how these expansions shaped our understanding of risk.

#### Incorporation of non-climatic hazards

By combining quantitative, spatial, and qualitative insights, the LRPF provided a more comprehensive understanding of multi-hazard exposure compared to the IPCC’s AR5 framework (Smith and Diedrich 2024). The LRPF decision support tools emphasised the impact of non-climatic hazards on rural livelihoods in Solomon Islands (Gillet and Tauti 2018; Filho et al. 2020; Van der Ploeg et al. 2020a). The prevalence of commercial logging as a ‘high concern’ hazard confirmed the need for a broader perspective on hazard exposure that goes beyond climate change (McDowell et al. 2016). Additionally, there were clear interactions and cumulative impacts occurring between climatic and non-climatic hazards. These complexities are widely evidenced in Solomon Islands (Minter and Van der Ploeg 2023), yet seldom addressed in adaptation projects which tend to give sole focus to climate change (Van der Ploeg et al. 2020a). This narrow focus has been evidenced to drive ineffective adaptation. For example, coral reef restoration initiatives in response to climate change have been hindered by sedimentation in the marine environment caused by nearby logging activities that were not considered in the planning process (Van der Ploeg et al. 2020b). This underscores the need for holistic approaches, like the LRPF, that can proactively identify the full spectrum of hazards faced by communities.

#### Expansion of socio-economic vulnerability indicators

The LRPF incorporated an expanded set of socio-economic indicators to provide a more holistic assessment of the factors influencing vulnerability. The framing of adaptive capacity indicators around capital asset categories (DFID 1999) made critical factors like social and human capital more explicit within the LRPF and its outputs. This included indicators like trust and collective action, which are commonly excluded from AR4-based studies (Smith and Diedrich 2024), yet have proven crucial for adaptation success (Diedrich et al. 2019; Ha’apio et al. 2019; Malherbe et al. 2020). This was evidenced in C3, where a previous project to enhance drinking water access through communal rainwater tanks faced long-term challenges due to emerging social tensions. Residents cited limited knowledge about fair and sustainable water use and low levels of trust as the cause of disputes over unequal water distribution. These unanticipated consequences eroded social capital, increasing the community’s vulnerability. By including these indicators within the LRPF, the potential for such issues is more likely to be recognised and discussed, allowing for proactive consideration of their potential impacts on adaptation efforts.

We recognise that the LRPF could be enhanced by the inclusion of other important measures such as the psychological processes that influence adaptive capacity, agency, and knowledge, and their application in addressing risk (Cinner et al. 2018; Torre-Castro et al. 2022). However, the comprehensiveness of the components and measures included in the LRPF necessitated, for the sake of pragmatism, the omission of certain factors typically explored in studies dedicated solely to one risk component, such as adaptive capacity. Shifting from the use of capital assets to more novel framings of adaptive capacity, such as the five domains proposed by Cinner et al. (2018) may offer an alternative pathway to integrate these factor that could be explored in future iterations of the LRPF.

Across communities, a lack of financial and physical capital emerged as key drivers of vulnerability, posing a significant barrier to adaptation. In Solomon Islands, this has led to a reliance on external stakeholders to fund community-based adaptation efforts (Van der Ploeg et al. 2020a). Participatory approaches like the LRPF can help ensure that externally funded responses align with local needs by providing evidence of key risk drivers and empowering communities to effectively communicate their adaptation priorities (Barrowman and Butler 2020).

Framing sensitivity around LSOs enabled the identification of critical community needs often overlooked in CBVAs (Smith and Diedrich 2024). Failing to address these factors can result in missed vulnerabilities and the misallocation of resources for adaptation (Ford et al. 2018). For instance, housing security is frequently disregarded within CBVAs (Smith and Diedrich 2024), yet it is critical for enabling individuals to engage in livelihood activities (Haddad et al. 2022). By categorising sensitivity into LSOs, the LRPF can more effectively establish adaptation priorities that address the unique challenges faced by each community. For example, while water and food security are critical issues across many rural Solomon Island communities (Basel et al. 2020), the urgency and nature of these challenges can vary significantly from one community to another. This variation underscores the importance of adopting a case-by-case approach to adaptation planning, a process that can be effectively facilitated by frameworks like the LRPF (Barrowman and Butler 2020).

We recognise that despite its expansion, the LRPF’s sensitivity analysis remains a basic assessment of the factors influencing a community’s ability to meet its basic needs (Frankenberger and McCaston 1998). This reflects the pragmatism necessary for gathering comprehensive data on all risk components, particularly considering factors such as participant fatigue during household surveys (Ambler et al. 2021). The integration of qualitative data was instrumental in bridging these gaps, providing deeper insights into factors contributing to risk that were overlooked in quantitative data collection methods.

Qualitative data provided valuable insights into connections across risk components, revealing critical interactions that shaped how a community experiences risk. This marks a significant advancement of the LRPF over traditional CBVA approaches, which often undervalue qualitative insights (Smith and Diedrich 2024). By incorporating qualitative data, the LRPF can offer a more nuanced understanding of risk that better reflects local contexts, establishes complex interdependencies among factors, and captures temporal dynamics (McDowell et al. 2016; Thiault et al. 2021). This included the incorporation of traditional knowledge, encompassing factors like cultural capital, which were not explicitly included within quantitative data collection methods. Such information is invaluable, given the critical role these factors play in enhancing rural livelihoods resilience in Solomon Islands (Basel et al. 2020).

#### Introduction of source dependence

Source dependence established a direct link between vulnerability and hazard exposure (Marshall 2011), providing insights into hazard specific impacts on rural livelihoods. While absent from most CBVA studies (Smith and Diedrich 2024), this information is vital in guiding adaptation planning. When paired with hazard exposure maps, source dependence allowed for the identification of high impact hotspots and helped prioritise adaptation measures that directly addressed these impacts in relation to the exposed source types (Marshall et al. 2013). This is especially important for communities that depend on a single source type to meet their LSOs. Such dependence was observed across LRPF communities, particularly concerning access to food and water. In Solomon Islands, single source dependence has become increasingly common due to the degradation of traditional source types as a result of resource exploitation activities like commercial logging (Anthonj et al. 2020; Basel et al. 2020).

Qualitative data offered valuable insights into the factors driving long-term shifts in source dependence, further helping to establish priority adaptation initiatives. In many cases, these initiatives involve the introduction of novel source types, which may necessitate investments in infrastructure (e.g. rainwater tanks), or inputs (e.g. novel crop varieties) (Basel et al. 2020). By combining insights on source dependence with vulnerability index outputs, we can refine adaptation strategies by identifying potential barriers to their implementation. As illustrated in this paper, for many communities, such barriers are tied to limited access to physical and financial capital (Van der Ploeg et al. 2020a). Identifying hazard exposure impacts that require urgent attention, and linking them to socio-economic vulnerability, demonstrates the critical role of source dependence in understanding and addressing rural livelihoods risk (Marshall 2011).

### Using LRPF decision support tools to support adaptation planning

By expanding the AR5 to address CBVA limitations in rural livelihood contexts, this paper facilitates a transition from vulnerability to risk assessment, providing a deeper understanding of the factors impacting rural livelihoods to foster more robust adaptation outcomes (Van Aalst et al. 2008). Given the continued popularity of CBVA approaches (Smith and Diedrich 2024), researchers and practitioners should pay closer attention to the potential benefits of risk-based approaches like the LPRF, as discussed in this paper (Marin-Puig et al. 2022). From an institutional perspective, transitioning to risk can also align research outputs with policy, where risk reduction has become a central focus (IPCC 2014; Hugel and Davies 2020). This alignment enhances the practical relevance of research outputs, facilitating their direct application in decision-making contexts (Dubois et al. 2019).

Together, the LRPF decision support tools guide priority adaptation initiatives that address complex interactions among risk drivers. While beneficial, the complexity of these tools may limit their direct use for community-based adaptation. To enhance their value, integrating LRPF tools into adaptation planning methods can make them more accessible and actionable for communities (Barrowman and Butler 2020). This integration can also strengthen adaptation planning by providing a mechanism to explicitly identify, understand, and address key risk drivers within adaptation responses. Many existing planning methods lack this insight, which can limit their capacity to effectively increase livelihoods resilience (Werners et al. 2021). LRPF decision support tools provide an opportunity to enhance adaptation planning methods by incorporating evidence-based insights on local risk drivers. This integration can ensure that adaptation efforts respond to the complex range of factors influencing rural livelihoods risk.

Integrating LRPF outputs into adaptation planning methods also provides an opportunity to account for differences in how adaptive capacity is expressed at the household level, versus how it is mobilised at the community scale. This allows adaptation strategies to be tailored to the unique strengths or needs of households, while also addressing collective requirements, such as coordinated action and resource sharing (Mortreux and Barnett 2017). Communicating LRPF outputs can facilitate this process by fostering active participation and informed decision-making regarding adaptation (Ensor et al. 2018). By encouraging collective recognition of key risk drivers, the LRPF can facilitate meaningful discussions about adaptation priorities, promoting a sense of collective ownership and responsibility for adaptation efforts (McNamara et al. 2020). Such engagement has been shown to significantly enhance the effectiveness and sustainability of locally led adaptation initiatives (Colloff et al. 2024). This highlights the need for CBRA approaches that go beyond simply assessing risk to effectively communicating findings, empowering communities to take proactive steps towards strengthening livelihoods resilience (Ensor et al. 2018).

The LRPF decision support tools can also serve as evidence-based resources to align externally funded adaptation efforts with local priorities. Although this alignment is intended, it often falls short in practice, as funders frequently adopt a ‘one-size-fits-all’ approach that emphasises their organisational goals, which may not necessarily align with local needs (Barrowman and Butler 2020). In Solomon Islands, this disconnect has resulted in numerous cases of misaligned and ineffective adaptation, undermining the potential benefits of external support for adaptation (Harohau et al. 2020; Roscher et al. 2021). Utilising LRPF outputs as a roadmap for designing interventions can help bridge this gap.

Stakeholders often rely on quantitative data to assess adaptation needs because it provides a standardised interpretation of risk that can be easily compared across communities (Nguyen et al. 2016). However, this approach overlooks the context-specific local factors that critically influence risk (Ford et al. 2018). In this context, the mixed-methods approach of the LRPF is especially valuable, as its qualitative data highlights the unique factors and complex interactions among risk factors that exist within a community.

### Future research directions

Our paper outlines the process of developing and implementing the LRPF to support rural livelihoods adaptation. Consequently, several analytical capabilities of the framework were deemed beyond the scope of this research. For instance, by collecting household-level data, the LRPF can be used to analyse intra-community vulnerability, enabling a better understanding of the specific factors driving livelihood impacts across different demographic groups or livelihood activities (Bolt and Bird 2003). This information is valuable in guiding adaptation efforts, ensuring that initiatives are beneficial and effective for all community members (Ford et al. 2018).

Additionally, the LRPF can explicitly inform temporal and spatial components of planning. For example, data on current risks could be used to envision how livelihoods may respond to future hazard predictions through the use of scenario planning methods (Colloff et al. 2024; Bennett et al. 2016). While hazard exposure maps could guide spatially explicit adaptation efforts that focus on reducing place-based risks across interconnected landscapes (Thiault et al. 2018; Diedrich et al. 2022). Finally, while the LRPF could be implemented in contexts beyond Solomon Islands, it would require adapting vulnerability indicators to accurately reflect the unique characteristics of rural livelihoods across different locations (Ford et al. 2018).

## Conclusions

This paper introduced and implemented the Livelihoods-Based Risk Profiling Framework (LRPF) as a holistic approach to support rural livelihoods adaptation in Solomon Islands. By addressing the limitations of CBVAs, the LRPF provided a deeper understanding of the factors driving rural livelihood risks. This required considering climatic and non-climatic hazard events, a broader range of socio-economic vulnerability indicators, and the role of source dependence. Translating LRPF outputs into decision support tools demonstrated the complex multi-hazard risk landscape faced by rural communities and the diverse socio-economic factors contributing to their vulnerability. By employing a mixed-methods approach, the LRPF achieved a nuanced understanding of risk, identifying key drivers and their interactions to inform targeted priority adaptation initiatives for rural livelihoods. Such information is vital in fostering more effective and sustainable adaptation outcomes in rural areas. At the community scale, LRPF outputs aimed to empower proactive decision making, supporting community members to take ownership over their adaptation responses. While at broader scales, LRPF outputs aimed to guide policymakers and external stakeholders in designing context-specific adaptation responses that align with local needs and realities. Ultimately, this paper demonstrates the importance of transitioning towards holistic risk assessment approaches that address the limitations of CBVAs, supporting more robust adaptation strategies to enhance rural livelihoods resilience.

## Supplementary Information

Below is the link to the electronic supplementary material.

Supplementary file1 (PDF 2099 KB)
