# Supplementary material for: Navigating risk: A holistic framework for supporting rural livelihoods adaptation in Solomon Islands
Source: Ambio. 2025 Mar 28;54(9):1496–513. doi: 10.1007/s13280-025-02162-4 (PMC12307829; doi:10.1007/s13280-025-02162-4)
Supplement: Supplementary file 1 — Supplementary file1 (PDF 2099 KB) [file 13280_2025_2162_MOESM1_ESM.pdf]

Ambio

Supplementary Information

This supplementary information has not been peer reviewed.

Title: **Risk-Based Livelihood Profiling to Support Community Adaptation Planning**

Risk-Based Livelihood Profiling to Support Community Adaptation Planning

Supplementary Information 1: LRPF Vulnerability Indicators (Page 1)

Methodological information for the development of Livelihoods-Based Risk Profiling Framework adaptive capacity and sensitivity indicators.

Supplementary Information 2: Household Survey (Page 38)

Household survey questionnaire used to collect information on vulnerability (i.e., adaptive capacity, sensitivity, and LSO source dependence) and household level hazard exposure for use within the Livelihoods-Based Risk Profiling Framework (LRPF)

Supplementary Information 3: Risk Interaction Diagrams (Page 51)

Risk interaction diagrams representing interactions between hazard exposure, LSO source dependence, sensitivity, and adaptive capacity indicators and their influence on priority livelihood initiatives within case study communities C1 (a), C2 (b), and C3 (c) in Solomon Islands.

Supplementary Information 4: Vulnerability Indicator Scores (Page 52)

The contribution of composite indicators and indicators of adaptive capacity and sensitivity to the overall vulnerability of C1, 2 and 3. Where: red = very high, orange = high, yellow = moderate, light green = low, and dark green = very low contribution to vulnerability.

Supplementary Information 1: LRPF Vulnerability Indicators

Human Capital

H1: Dependency Ratio

Indicator Summary	
**Indicator:** The ratio of dependent (i.e., non-working) to non-dependent household members (i.e., working).	
**Indicator Description:** The number of household members that do not contribute to livelihood activities compared to the number of household members that contribute to livelihood activities.	
**Measuring Unit:** Ratio of dependent to non-dependent household members.	
**Contribution to Adaptive Capacity**	
**Summary:** A household with a higher dependency ratio will have a reduced capacity to adapt to hazard exposure.	
**Justification:** The dependency ratio highlights the potential responsibility that will be placed on economically active members of a household by dependents (i.e., children, the elderly and additional non-working household members). In the context of this approach, a higher dependency ratio represents lower adaptive capacity reflecting instances where economically active household members face a greater responsibility to support and provide for children and older or non-working household members (United Nations 2007). Households with a high dependency ratio are considered to be more vulnerable to hazards given that the young, elderly and non-working are more at risk of impact from exposure, and that a smaller proportion of the household is responsible in acquiring the necessary resources to provide for livelihood requirements (Mulyana et al. 2013).	
**Indicator Method**	
**Indicator Origin & Adaptations**	
**Origins:** This indicator calculates the dependency ratio within a household (non-working individuals/working individuals*100) rather than the more widely applied technique which calculates dependency ratios across populations (e.g., UN 2007). The household ratio method was applied to enable differences in adaptive capacity to be observed from within sub-groups of a community. By applying the household ratio method, this indicator can identify specific households that have a limited number of working adults to support dependents. Household level dependency ratios have been utilised in numerous livelihoods-based studies (Fang 2022; Hadley et al. 2011). These indicators typically calculate the number of working age (i.e., aged 15-64) to non-working age household members (i.e., children and the elderly).	
**Adaptations:** The method applied in this approach considered instances where working age adults did not contribute to a household, or non-working aged adults did. This information was obtained via data on the main livelihood activities conducted by each household member, with individuals conducting 'no activities' considered as dependents. By calculating dependency ratio based on contribution to livelihood activities, a more accurate representation for each household was obtained. This enabled factors such as disability, physical and mental health issues, engagement in education, etc., to be represented as a component of the dependency ratio. If a household did not contain any non-dependent members (i.e., no individuals conducted livelihood activities), then a high dependency ratio was assigned (e.g., 4:1 or 400%) and the household was assumed to have very low adaptive capacity in relation to this indicator. This assumed (validated during participant observation in Solomon Islands) that non-working households rely on family or community members for support.	
**Data Collection Instrument**	
**Household Survey Question(s):** What is the main livelihood activity conducted by each household member?	
**Processing Steps**	
**Data Type**	Integer/Continuous

1. Classify responses into dependents (1) those who are not contributing to livelihood activities, irrespective of age and non-dependents (2) those who are contributing to livelihood activities irrespective of age.
2. Calculate the dependency ratio using the formula (number of non-working household members/number of working household members)*100).
3. Assign dependency ratio data ranks using below categories (based on equal interval binning from dependency ratio data range).

Indicator Rankings			
**Adaptive Capacity Rank**		**Definition**	**Description**
1	Very Low	321-400%	Very High Dependency Ratio
2	Low	241-320%	High Dependency Ratio
3	Medium	161-240%	Mid-Level Dependency Ratio
4	High	81-160%	Low Dependency Ratio
5	Very High	0-80%	Very Low Dependency Ratio

Key References

1) Fang, D. 2022. The effect of household dependency ratio on the mental health of the workforce. Evidence from China. *Frontiers in Public Health.* 10, pp.1-11.
2) Hadley, C., Belachew, T., Lindstrom, D. & Tessema, F. 2011. The shape of things to come. Household dependency ratio and adolescent nutritional status in rural and urban Ethiopia. *American Journal of Physical Anthropology.* 114, Pp.643-652.
3) Mulyana, W., Dodman, D., Zhang, S. & Schensul, D. 2013. *Climate vulnerability and adaptation in the Semarang Metropolitan Area: A spatial and demographic analysis.* Technical Briefing. IIED. London. Pp.1-4.
4) United Nations. 2007. *Dependency Ratio Indicator:* Available at: https://www.un.org/esa/sustdev/natlinfo/indicators/methodology_sheets/demographics/dependency_ratio.pdf

H2: Health Condition

Indicator Summary

Indicator: The number of times in the last year household members missed their livelihood responsibilities due to being sick, ill or injured.

Indicator Description: The amount of time household members were not able to go to work, attend the garden, collect food (e.g., fish or forage), go to school, etc., due to being sick, ill or injured. This proxy for health condition was measured on a scale reflecting where (1) livelihood activities have never been missed, to where (5) livelihood activities are missed all the time. A proxy measure of health condition was utilised given data limitations in household survey questions that attempted to obtain a more detailed understanding of the physical and mental health status of household members. Where such questions were asked (i.e., 'how many members of your household suffer from a long-term health condition or disability', and 'would you mind sharing these conditions or disabilities') data response bias was observed. This is likely attributed to the overly intimate nature of health associated questions in the rural communities where the LRP was operationalised.

Measuring Unit: Number of time livelihood activities were missed in the last year (5-point Likert Scale: (1) never missed – (5) always missed).

Contribution to Adaptive Capacity

Summary: As the duration of time household members miss their livelihood responsibilities increases, their capacity to adapt to hazard exposure decreases.

Justification: The use of 'missed school or work' (i.e., livelihood responsibilities) as a proxy measure for health condition is an indicator that has been widely applied in prior vulnerability assessments (Smith & Diedrich 2024). Mitchell & Bates (2011) and Marmot et al. (1995) evidenced this indicator to deliver an accurate representation of health status, finding a direct relationship between ill health and productivity loss (i.e., reduced amount and quality of labour). The significant negative relationship between poor health condition and an individual's ability to contribute to livelihood responsibilities highlights the critical importance of this indicator as a component of adaptive capacity (Bleakley 2010). In the context of hazard exposure, households with high levels of ill health are assumed to be less capable of contributing to and participating in activities that may support households in adapting to or overcoming impacts from hazard exposure (Smith 2008).

Indicator Method

Indicator Origin & Adaptations

Origins: Measuring health security using the amount a household has missed 'work or school' due to ill health has been used as an indicator in many prior community based vulnerability assessments (e.g., Oo et al. 2018; Owusu et al, 2021; Panthi et al. 2016). In these assessments, this indicator is worded as 'the amount of times household members missed school or work due to sickness, illness or injury'.

Adaptations: A minimal change in question wording was applied to cover the range of livelihood responsibilities that are conducted by rural households. By rephrasing 'school or work' as 'livelihood

responsibilities' and providing a range of examples (e.g., not able to go to work, attend the garden, collect food (e.g., fish and forage), go to school, etc.,), the LRP approach aims to reduce confusion by survey respondents in relation to what constitutes 'work'.

Data Collection Instrument

Household Survey Question: Were there any times in the past year that members of your household missed their livelihood responsibilities due to being sick, ill or injured? (5) no times; (4) a few times; (3) several times; (2) many times; (1) all the time. (e.g., not able to go to work, attend the garden, collect food (e.g., fish and forage), go to school, etc.,).

Data Type Ordinal

1. Input survey data based on the 1-5 scale whereby high adaptive capacity = (5 - household members missed their livelihood responsibilities no times), and low adaptive capacity = (1 - household members missed their livelihood responsibilities all the time).

Indicator Rankings

**Adaptive Capacity Rank**		**Definition**	**Description**
1	Very Low	All the time	Household missed their responsibilities all the time due to ill health.
2	Low	Many times	Household missed their responsibilities many times due to ill health.
3	Medium	Several times	Household missed their responsibilities several times due to ill health.
4	High	A few times	Household missed their responsibilities a few times due to ill health.
5	Very High	No times	Household never missed their responsibilities due to ill health.

Key References

1) Bleakley, H. 2010. Health, human capital, and development. *Annual Review of Economics.* 2. Pp.283-310.
2) Marmot, M., Feeney, A., Shipley, M., North, F. & Syme, L.S. 1995. Sickness absence as a measure of health status and functioning: from the UK Whitehall II study. *Journal of Epidemiological Community Health.* 49(2), Pp.124-130.
3) Mitchell, J.R. & Bates, P. 2011. Measuring health-related productivity loss. *Population Health Management.* 14(2), Pp.93-98.
4) Oo, A.T., Van Huylenbroeck, G. and Speelman, S., 2018. Assessment of climate change vulnerability of farm households in Pyapon District, a delta region in Myanmar. *International Journal of Disaster Risk Reduction*, *28*, pp.10-21.
5) Owusu, V., Ma, W., Emuah, D. and Renwick, A., 2021. Perceptions and vulnerability of farming households to climate change in three agro-ecological zones of Ghana. *Journal of Cleaner Production*, *293*, p.126154.
6) Panthi, J., Aryal, S., Dahal, P., Bhandari, P., Krakauer, N.Y. and Pandey, V.P., 2016. Livelihood vulnerability approach to assessing climate change impacts on mixed agro-livestock smallholders around the Gandaki River Basin in Nepal. *Regional Environmental Change*, *16*(4), pp.1121-1132.
7) Smith, R.K. 2008. Climate change and health: Mitigating, adapting and suffering: How much of each? *Annual Review of Public Health.* 29, Pp.23-39.
8) Smith, B. and Diedrich, A., 2024. A systematic review of current progress in community based vulnerability assessments. *Regional Environmental Change*, *24*(1), pp.1-17.

H3: Access to Healthcare

Indicator Summary

Indicator: The amount of time a household can access healthcare in times of need.

Indicator Description: The amount of time a household is capable of accessing healthcare in times of need ranging from (5) never; (4) rarely; (3) occasionally; (2) frequently and (1) all the time.

Measuring Unit: Access to healthcare (amount of time) (5-Point Likert Scale: 1 (all the time) to 5 (never)).

Contribution to Adaptive Capacity

Summary: Households that struggle to access healthcare in times of need will have less capacity to adapt to hazard exposure.

Justification: Access to healthcare is essential to build the resilience of a population and protect communities from a wide range of health issues. However, limited access to healthcare can reduce the ability of a household to detect, respond and reduce impact from health issues (Peters et al. 2008). Without healthcare access, treatable issues can become life threatening, and result in higher levels of morbidity within a community (Nugent 2008). Limited access to healthcare can additionally increase hazard exposure impacts, especially when a hazard has direct health implications. Where healthcare access is limited, hazard induced health issues can fail to receive essential and timely medical attention, treatment and rehabilitation

(Ebi & Bowen 2016). Failure to receive preventative healthcare (e.g., vaccinations) can also increase the risk of transmissible diseases within a community (Kein 2008).
**Indicator Development**
**Indicator Origin & Adaptations**
**Origins:** This indicator was developed in alignment with the World Health Organisation and United Nations access to essential health services (UN 2020a). The specific indicator utilised for this assessment comprised the amount of time a household lacks access to healthcare. This indicator has been utilised in numerous CBVAs as a metric of health security (e.g., Ahmad & Afzel 2022; Amevenku 2019; Phan 2019).
**Adaptations:** A 5-point Likert scale was developed to represent the amount of time healthcare was not accessible for a household. This question type was selected for simplicity and ease of use, and to help prevent respondent fatigue given the number of indicators included in the LRP approach.
**Sampling Method**
Household Survey Question: In the past year, were there ever times when you or members of your household couldn't access healthcare? (5) never; (4) rarely; (3) occasionally; (2) frequently; (1) all the time.

**Data Type**	Ordinal

1. Input household survey data ranging from (1) household never has issues accessing healthcare (very low sensitivity) to (5) always has issues accessing healthcare (very high sensitivity) into standardised indicator rankings.

Indicator Rankings

**Adaptive Capacity Rank**		**Definition**	**Description**
1	Very Low	All the Time	Household can never access healthcare.
2	Low	Frequently	Household frequently cannot access healthcare.
3	Medium	Occasionally	Household occasionally cannot access healthcare.
4	High	Rarely	Household rarely cannot access healthcare.
5	Very High	Never	Household always has access to healthcare.

Key References

1) Ahmad, D. & Afzal, M. 2022. Flood hazards and livelihood vulnerability of flood prone farm dependent Bait households in Pubjab, Pakistan. *Environmental Science & Policy Research.* 29. Pp.11533-11547.
2) Amevenku, F.K., Kuworno, J.K., Seini, A.W., Osei-Asare, Y.B. & Anim-Soumah, H. 2019. Livelihood vulnerabilities and diversification of fishing households in Ghana. *Development in Practice.* 29(7), Pp.867-881.
3) Ebi, L.K. & Bowen, K. 2016. Extreme events as sources of health vulnerability: Drought as an example. *Weather and C Climate Extremes.* 11, Pp.95-102.
4) Kein, E.M. 2008. Building human resilience: The role of public health preparedness and response as an adaptation to climate change. *American Journal of Preventative Medicine.* 35(5). Pp.508-516.
5) Nugent, R. 2008. Chronic diseases in developing countries. *Annals of the New York Academy of Sciences.* 1136(1). P Pp.70-79.
6) Peters, H.D., Garg, A., Bloom, G., Walker, D.G., Breiger, R.W. & Rahman, H.M. 2008. Poverty and access to health care in developing countries. *Annals of the New York Academy of Sciences.* 1136(1). Pp.161-171.
7) Phan, L.T., Jou, S.C. & Lin, J.H. 2019. Gender inequality and adaptive capacity: The role of social capital on the impacts of climate change in Vietnam. *Sustainability.* 11(5). Pp.1257-1263.
8) Smith, B. and Diedrich, A., 2024. A systematic review of current progress in community based vulnerability assessments. *Regional Environmental Change*, *24*(1), pp.1-17.
9) United Nations. 2020a. *SDG Indicator Metadata. Goal 2.8.1. Coverage of essential health services.* United Nations. Geneva, Switzerland. Pp.1-12.

H4: Livelihood Diversity Index

**Indicator Summary**
**Indicator:** The number of unique livelihood activities that members of a household are engaged in.
**Indicator Description:** The number of unique livelihood activities (e.g., fishing, agriculture, tourism) that members of a household participate in.
**Measuring Unit:** Number of livelihood activities.
**Contribution to Adaptive Capacity**
**Summary:** The greater the number of livelihood activities a household engages in, the more capacity they have to adapt to hazard exposure.
**Justification:** Livelihood diversification is defined as the 'process by which rural families construct a diverse portfolio of activities and social support capabilities in order to survive and improve their standard of living'

(Ellis 1998). A widespread number of studies report that livelihood diversification is beneficial for reducing livelihood risks and vulnerability (Liao & Fei 2017; Zhang et al. 2019), improving livelihood resilience and sustainability (Ifejika et al. 2014) relieving pressure on the environment (Hao et al. 2015), and alleviating poverty (Igwe 2013). Zhang et al. (2019) found a limited livelihood portfolio to be the most important driver of vulnerability in relation to climate change, highlighting the importance of this variable as a metric for use in vulnerability index.

Indicator Method

Indicator Origin & Adaptations

Origins: The index method used in this approach is representative of 'household occupational diversity index', which assesses the variety of unique occupations or activities pursued by household members throughout a given year. The method was replicated from Hahn et al. (2009), who use 'number of livelihood activities reported by a household' to measure agricultural livelihood diversity. In this method, the diversification index is represented as an inverse number, reflecting that an increase in livelihood activities decreases vulnerability.

Adaptations: The Hahn et al. (2009) indicator measures agricultural livelihood diversity, while the LRP addresses livelihood activities as any 'action, task or endeavour that a household engages in to secure their means of living and sustain their livelihood' (DFID 1999). The use of an inverse measure (as seen in Hahn et al. 2009) was not applied during the calculation of this indicator. This is due to the classification of livelihood diversity as a component of adaptive capacity. In this context, an increase in the number of activities conducted by a household will result in a direct increase in adaptive capacity, voiding the requirement for an inverse calculation.

Data Collection Instrument

Household Survey Question(s): What is the primary livelihood activity conducted by each household member? What other livelihood activities does each household member (including yourself) participate in?

**Data Type**	Integer

1. List the types of primary livelihood activities and additional livelihood activities conducted within a household.
2. Sum the number of UNIQUE livelihood activities conducted by household members, to calculate the final livelihood diversity score.
3. Assign ranking scores based on the data range.

Indicator Rankings

**Rank**		**Definition**	**Description**
1	Very Low	0-1 Activities	Household participates in 0-1 livelihood activities
2	Low	2-3 Activities	Household participates in 2-3 livelihood activities.
3	Medium	4-5 Activities	Household participates in 4-5 livelihood activities.
4	High	6-7 Activities	Household participates in 6-7 livelihood activities.
5	Very High	8-9 Activities	Household participates in 8-9 or more activities.

Key References

1) DFID. *Sustainable Livelihoods Guidance Sheets.* Department for International Development. London, United Kingdom. Pp.1-150.
2) Ellis, F. 1998. Rural livelihood diversification in sub-Saharan Africa. A literature review. *Journal of Development Studies.* 51, pp.1125-1138.
3) Hahn, B.M., Riederer, M.A. & Foster, O.S. 2009. The Livelihood Vulnerability Index. A pragmatic approach to assessing risks from climate variability and change – A case study in Mozambique. *Global Environmental Change.* 19(1), Pp.74-88.
4) Hao, H., Zhang, J., Li, X., Zhang, H. & Zhang, Q. 2015. Impact of livelihood diversification of rural households on their ecological footprint in agro-pastoral regions of northern China. *Journal of Arid Land.* 7, pp.653-664.
5) Ifejika. S., Wiesmann, U. & Rist, S. 2014. An indicator framework for assessing livelihood resilience in the context of social-ecological dynamics. *Global Environmental Change.* 28(1), pp.109-119.
6) Igwe, P.A. 2013. Rural non-farm livelihood diversification and poverty reduction in Nigeria. PhD Thesis. University of Plymouth.
7) Liao, C. & Fei, D. 2017. Sedentarization as constrained adaptation: Evidence from pastoral regions in far north-western China. *Human Ecology*. 45, pp.23-35.
8) Zhang, Q., Zao, X. & Tanga, H. 2019. Vulnerability of communities to climate change: Application of the livelihood vulnerability index to an environmentally sensitive region of China. *Climate & Development.* 11, pp.525-542.

H5: Extent of Coping Strategies

Indicator Summary

Indicator: The extent to which a household perceives they can cope with locally relevant hazard exposure.

Indicator Description: This indicator determines the extent to which a household believes they are capable of coping with exposure to a range of locally relevant shock and stressor events (i.e., commercial logging, reductions in wild fish availability, sea level rise, flooding, increased rainfall, reduced rainfall, and temperature increase). Coping strategies were measured on a 5-point Likert scale ranging from (1) strongly disagree (i.e., a household believes they have no strategies to cope with hazard exposure), to (5) strongly agree (i.e., a household believes they have a lot of strategies to cope with hazard exposure). The relative importance of a given shock or stressor to community members was used to weight, and combine the overall extent of coping strategies in a household. For example, coping strategies related to sea level rise were given a higher weighting in communities experiencing significant exposure to this stressor compared to other events (e.g., commercial logging). By incorporating these weightings inter-community variation in hazard exposure can be recognised, reflected in the relative importance assigned to coping strategies. Weighting scores for each community were obtained during focus group workshops where participants ranked the relative importance of locally relevant shock and stressor events.

Measuring Unit: Extent of coping strategies (five-point Likert Scale: (1) none to (5) a lot of coping strategies)

Contribution to Adaptive Capacity

Summary: A household perceived to have a greater amount of coping strategies to locally relevant shock and stressor events will have greater capacity to adapt to hazard exposure in the long-term.

Justification: Short-term coping capacity has become increasingly important as a measure to protect communities from hazard exposure (Tanner et al. 2015). Defined by the IPCC (2014) as 'the ability of a community or individual to address, manage, and overcome adverse conditions in the short to medium term', coping capacity represents the strategies available to meet immediate needs and manage the impacts of hazard exposure. The short-term nature of coping is differentiated from longer-term adaptive capacity, which focuses on the ability of a community or individual to adjust, learn and evolve in the face of hazard exposure (Whitney et al. 2017). Despite these differences, there is correlation between the two terms, with strong coping capacity shown to positively contribute to adaptive capacity (Smit & Wandel 2006). This correlation is due to shared attributes that support both coping and adaption to hazard events (Yohe & Tol 2002). As such, households with a greater number of coping strategies will be more likely to both recover quickly and adapt to hazard events in the short-term and long-term (Smit & Wandel 2006).

Indicator Method

Indicator Origin & Adaptations

Origins: Coping strategies are included in a minimal number of CBVAs, and commonly relate to a pre-dictated list of strategies related to a single shock or stressor event (e.g., Maikhuri et al. 2017). However, Likert scale questions have also been utilised to measure individual perceptions of coping capacity (e.g., Perlaviciute 2021). The range of clear responses in a Likert scale question, commonly ranging from 'bad' to 'good' enables participants to reflect upon and indicate the extent of their coping strategies in a manner that can be compared across households (Batterton & Hale 2017).

Adaptations: Given the range of shock and stressor events addressed in the LRP, coping strategies were determined using a perception based measure. To do so, a Likert scale question was developed that enabled easy reflection on the extent of coping strategies held within a household based on a 5-point response.

Data Collection Instrument

Household Survey Question: Does your household have ways of coping with these problems?
(1) strongly disagree; (2) disagree; (3) neither agree nor disagree; (4) agree; (5) strongly agree.

**Data Type**	Ordinal

1. Determine focus group outputs ranking hazards based on perceived impacts to local livelihoods.
2. Input survey responses onto a 1-4 scale (from (0) strongly disagree (i.e., household has no coping strategies), to (4) strongly agree (i.e., household has a lot of knowledge of coping strategies)), to obtain an overall understanding of the extent of household coping strategies for each type of hazard.
3. Multiply coping strategy scores by the rank of a given hazard.
4. Add coping strategy types together to calculate household scores, transformed into a % of the maximum possible score of combined ranks.
5. Rank household coping strategies using the following ranking categories.

Indicator Rankings			
**Adaptive Capacity Rank**		**Definition**	**Description**
1	Very Low	0-20%	Household has no strategies to cope with hazard exposure.
2	Low	21-40%	Household has very few strategies to cope with hazard exposure.
3	Medium	41-60%	Household has some strategies to cope with hazard exposure.
4	High	61-80%	Household has many strategies to cope with hazard exposure.
5	Very High	81-100%	Household has a lot of strategies to cope with hazard exposure.

Key References

1) Batterton, A.K. & Hale, N.K. 2017. The Likert Scale, what is it and when to use it. *Phalanx.* 50(2), Pp.32-39
2) IPCC. 2014. *Emergent risks and key vulnerability.* In: AR5 Climate Change 2014: Impacts, Adaptation and Vulnerability. Intergovernmental Panel on Climate Change. Geneva, Switzerland.
3) Maikhuri, K.R., Nautiyal, A., Jha, K.N., Rawat, L.S., Maletha, A., Phondani, P.C., Bahugana, M.Y. & Bhatt, C.G. 2017. Socio-ecological vulnerability: Assessment and coping strategy to environmental disaster in Kedarnath valley, Uttarakhand, Indian Himalayan Region. *International Journal of Disaster Risk Reduction.* 25. Pp.111-124.
4) Perlaviciute, G.S.L. 2021. Development and validation of a climate change perceptions scale. *Journal of Environmental Psychology.* 76, Pp.101-152.
5) Tanner, T., Lewis, D., Wrathall, D., Bronen, R., Cradock-Henry, N., Huq, S., Lawless, C., Nawrotzki, R., Prasad, V., Rahman, M.D., Alaniz, R., King, K., McNamara, K., Nadiruzzaman, M.D., Henley-Shephard, S. & Thomalla, F. 2015. Livelihood resilience in the face of climatic change. *Nature Climate Change.* 1, pp.23-26.
6) Whitney, C.K., Bennett, N.J., Ban, N.C., Allison, E.H., Armitage, D., Blythe, J.L., Burt, J.M., Cheung, W., Finkbeiner, E.M., Kaplan-Hallman, M., Perry, I., Turner, N.J. & Yumagulova, L.L. 2017. Adaptive capacity: from assessment to action in coastal social-ecological systems. *Ecology and Society.* 22, pp.22-34.
7) Yohe, G. & Tol, J.S.R. 2002. Indicators for social and economic coping capacity – moving toward a working definition of adaptive capacity. *Global Environmental Change.* 12(1), Pp.25-40.

Financial Capital

F1: Household Savings

Indicator Summary

Indicator: The total amount of savings accumulated by a household.

Indicator Description: The total amount of savings in Solomon Islands Dollar (SI$) that has been accumulated by a household based on the following categories (1) none; (2) less than $250; (3) $250-500; (4) $500-1000; (5) more than $1000.

Measuring Unit: Solomon Island Dollars (five-point Likert Scale: None – More than SI$1000).

Contribution to Adaptive Capacity

Summary: The greater the amount of savings accumulated by a household, the greater their capacity to adapt to hazard exposure.

Justification: Low amounts of household savings can negatively impact adaptive capacity by reducing the availability of a financial cushion to absorb shocks and stressor events (Daramola et al. 2016). By holding savings, household are provided with a safety net that can be used to proactively adapt to challenges that exist within the local environment (Susilo et al. 2021). Low savings have also been shown to interact with limited resource access in relation to healthcare, education and other basic services, and can perpetuate a cycle of limited adaptive capacity across generations in situations where access to these resources is monetary dependent (Jones et al. 2022). The link between low savings and adaptive capacity has been empirically justified by Daramola et al. (2016) who found households without personal savings experience a 13.87x greater impact from hazard exposure than households with savings.

Indicator Method

Indicator Origin & Adaptations

Origins: Amount of household savings is a standardised indicator used to understand a households' accumulated funds and is applied by many agencies including the WorldBank (2020) and United Nations (2005) in addition to many published CBVAs (Smith & Diedrich 2024).

Adaptations: The savings categories (and associated values) for this indicator were developed in coordination with local experts and represent suitable income levels for rural communities in Solomon Islands. All savings-related questions were asked in the local currency of Solomon Island Dollars.

Data Collection Instrument

Household Survey Question: Approximately how much money does your household hold in savings? (1) none; (2) less than $250; (3) $250-500; (4) $500-1000; (5) more than 1000; (0) don't know; (00) refuse to answer.

**Data Type**	Ordinal

1. Input household survey data relating to household savings.
2. Identify instances where households responded with 'don't know' or 'refuse to answer' and use average data imputation to replace this response. Average data imputation was identified as an appropriate mechanism to replace missing data given significant comparability across households in a community in relation to savings and the low rate of missing data (i.e., <5%) across the sample.
3. Rank household savings on a scale reflecting adaptive capacity (see below) ranging from very low (no savings) to very high (more than SI$1000 savings).

Indicator Rankings

Adaptive Capacity Rank		Definition	Description
1	Very Low	None	Household has no savings.
2	Low	Less than $250	Household has savings valued at less than SI$250.
3	Medium	$251-500	Household has savings valued between SI$251-500.
4	High	$501-1000	Household has savings valued between SI$501-1000.
5	Very High	More than $1000	Household has savings valued at more than SI$1000.

Key References

1) Daramola, Y.A., Oni, T.O., Ogundele, O. & Adesanya, A. 2016. Adaptive capacity and coping response strategies to natural disasters. A study in Nigeria. *International Journal of Disaster Risk Reduction.* 15, Pp.132-147.
2) Jones, L.H.B., Unsworth, F.K.R., Nordlund, M.L., Elkof, S.J., Ambo-Rappe, R., Carly, F., Jiddawi, S.N., Nafie, A.Y., Udagedara, S. & Cullen-Unsworth. 2022. Dependence on seagrass fisheries governed by household income and adaptive capacity. *Ocean & Coastal Management.* 225, Pp.106-126.
3) Susilo, E., Purwanti, P., Fattah, M., Qurrata, A.V. & Namaditya, S.B. 2021. Adaptive coping strategies towards seasonal change impacts: Indonesian small-scale fisherman household. *Heliyon.* 7, Pp.681-692.
4) Smith, B. and Diedrich, A., 2024. A systematic review of current progress in community based vulnerability assessments. *Regional Environmental Change*, *24*(1), pp.1-17.
5) United Nations. 2005. *Household sample surveys in developing and transition countries.* Department of Economic and Social Affairs, Statistics Division. United Nations, New York. Pp.1-655.
6) WorldBank. 2012. *Living standards measurement survey.* WorldBank, Washington DC. Pp.1-17.

F2: Household Income

Indicator Summary

Indicator: The amount of income a household acquires in a one-month period.

Indicator Description: The amount of income (in Solomon Island Dollars) a household makes from livelihood activities in a one-month period based on the following income categories 1) less than $250; 2); $250-500 3); $500-1000 4) $1-2000; 5) more than $2000.

Measuring Unit: Solomon Island Dollars (five-point Likert Scale: None – More than SI$2000).

Contribution to Adaptive Capacity

Summary: The greater a household's total monthly income, the greater their capacity to adapt to hazard exposure.

Justification: Low levels of income can negatively impact a household's ability to adapt to hazard exposure (Jin et al. 2016). This occurs through a number of mechanisms, including i) limited resource access; ii) high dependence on existing local resources; and iii) restricted access to infrastructure and services (Jones et al. 2022; Susilo et al. 2021). For example, households with low-income will have fewer available finances to invest in adaptive measures (i.e., resources) that can reduce potential impact from hazard exposure (Jones et al. 2022). This may also occur in instances where local natural resources (that households depend on for basic needs) are eroded (Susilo et al. 2021). In such instances, low-income households may not be capable of investing in alternate monetary-based resources that are available within or outside of the community (Jones et al. 2022). Finally, low-income households often have lower access to healthcare, education, sanitation, electricity etc., that contribute to adaptive capacity (Susilo et al. 2021).

Indicator Method

Indicator Origin & Adaptations

Origins: Average monthly income is a standardised indicator used to understand household income rates. This indicator is utilised by many agencies including the WorldBank (2020) and United Nations (2005) and within prior CBVA studies (Smith & Diedrich 2024).

Adaptations: The income categories (and associated values) for this indicator were developed in coordination with local experts and represent suitable income levels for rural communities in Solomon Islands. All income-related questions were asked in the local currency of Solomon Island Dollars.

Data Collection Instrument

Household Survey Question: How much money does your household make on average in one month? (1) less than $250; (2); $250-500 (3) $500-1000; (4) $1-2000; (5) more than $2000; (0) Don't know; (00) Refused to answer.

Data Type	Ordinal

1. Input household survey data relating to monthly income rates.
2. Identify instances where households responded with 'don't know' or 'refuse to answer' and use average data imputation to replace this response. Average data imputation was identified as an appropriate mechanism to replace missing data given significant comparability across households in relation to income level, and the low rate of missing data (i.e., <5%) across the sample.
3. Rank monthly income on a scale reflecting adaptive capacity (see below) ranging from very low (less then SI$250) to very high (more than SI$2000).

Indicator Rankings

Adaptive Capacity Rank		Definition	Description
1	Very Low	Less than $250	Household has a monthly income less than SI$250
2	Low	$251-500	Household has a monthly income between SI$251-500
3	Medium	$501-1000	Household has a monthly income between SI$501-1000
4	High	$1001-2000	Household has a monthly income between SI$1001-2000
5	Very High	More than $2000	Household has a monthly income of more than SI$2000

Key References

1) Jin, J., Wang, W. & Wang, X. 2016. Adapting agriculture to the drought hazard in rural China: household strategies and determinants. *Natural Hazards.* 82, Pp.1609-1619.
2) Jones, L.H.B., Unsworth, F.K.R., Nordlund, M.L., Elkof, S.J., Ambo-Rappe, R., Carly, F., Jiddawi, S.N., Nafie, A.Y., Udagedara, S. & Cullen-Unsworth. 2022. Dependence on seagrass fisheries governed by household income and adaptive capacity. *Ocean & Coastal Management.* 225, Pp.106-126.
3) Smith, B. and Diedrich, A., 2024. A systematic review of current progress in community based vulnerability assessments. *Regional Environmental Change*, *24*(1), pp.1-17.
4) Susilo, E., Purwanti, P., Fattah, M., Qurrata, A.V. & Namaditya, S.B. 2021. Adaptive coping strategies towards seasonal change impacts: Indonesian small-scale fisherman household. *Heliyon.* 7, Pp.681-692.
5) United Nations. 2005. *Household sample surveys in developing and transition countries.* Department of Economic and Social Affairs, Statistics Division. United Nations, New York. Pp.1-655.
6) WorldBank. 2012. *Living standards measurement survey.* WorldBank, Washington DC. Pp.1-17.

F3: Household Expenditure

Indicator Summary

Indicator: Household monthly expenditure on basic needs (e.g., food, water, housing, energy and healthcare).

Indicator Description: The approximate amount a household spends per month on key basic needs including food, water, housing, energy and healthcare. Expenditure is based on the following categories: (1) none; (2) less than $250; (3) $250-500; (4) $500-1000; (5) more than $1000.

Measuring Unit: Solomon Island Dollars (5-point Likert Scale: None – More than SI$1000).

Contribution to Adaptive Capacity

Summary: The greater a household's monthly expenditure, the greater their capacity to adapt to hazard exposure.

Justification: Expenditure has been used as a proxy indicator for wealth by Cinner et al. (2015) when evaluating adaptive capacity. In this context, higher expenditure reflects greater overall wealth, and thus greater adaptive capacity. This correlation has been empirically validated in numerous other studies; where increased expenditure was attributed to greater capacity for involvement in the formal economy, and

increased ability to purchase resources required to achieve livelihood security (e.g., food, water, energy and healthcare) (Defiesta & Rapera 2014; Thathsarani & Gunaratne 2018).

Indicator Method

Indicator Origin & Adaptations

Origins: Household expenditure is a commonly utilised indicator applied in prior CBVA studies (Smith & Diedrich 2024), and household expenditure per annum is the official indicator adopted by the Solomon Islands National Statistics Office within national surveys (SINS 2012). Expenditure-based questions commonly revolve around a household's total fortnightly (e.g., Cinner et al. 2015), or monthly, expenditure (e.g., Sekhampu & Niyimbanira 2013) across all livelihood outcome categories (e.g., food, water, housing, energy, healthcare).

Adaptations: Monthly expenditure was selected as an indicator for this approach to best capture potential fluctuations, trends, and variation in expenses through time. The expenditure categories (and associated values) for this indicator were developed in coordination with local experts and represent suitable income levels for rural communities in Solomon Islands. All expenditure-related questions were asked in the local currency of Solomon Island Dollars.

Data Collection Instrument

Household Survey Question: Approximately how much money does your household spend in one month? (1) no expenditure; (2); less than $250 (3) $250-500; (4) $500-1000; (5) more than $1000; (0) Don't know; (00) Refused to answer.

Data Type Ordinal

1. Input household survey data relating to monthly expenditure.
2. Identify instances where households responded with 'don't know' or 'refuse to answer' and use average data imputation to replace this response. Average data imputation was identified as an appropriate mechanism to replace missing data given significant comparability across households in relation to expenditure, and the low rate of missing data (i.e., <5%) across the sample.
3. Rank monthly expenditure on a scale reflecting adaptive capacity (see below) ranging from very low (no expenditure) to very high (monthly expenditure more than SI$1000).

Indicator Rankings

Adaptive Capacity Rank		Definition	Description
1	Very Low	No Expenditure	Household has no monthly expenditure
2	Low	Less than $250	Household's monthly expenditure is less than SI$250
3	Medium	$251-500	Household's monthly expenditure is between SI$251-500
4	High	$501-1000	Household's monthly expenditure is between SI$500-1000
5	Very High	More than $1000	Household's monthly expenditure is more than SI$1000

Key References

1) Cinner, E.J., Huchery, C., Hicks, C.C., Daw, M.T., Marshall, N., Wamukota, A. & Allison, H.E. 2015. Changes in adaptive capacity of Kenyan fishing communities. *Nature Climate Change.* 5, Pp.872-876.

2) Defiesta, G. & Rapera, L.C. 2014. Measuring adaptive capacity of farmers to climate change and vulnerability: Application of a composite index to an agricultural community in the Philippines. *Journal of Environmental Science and Management.* 17(2), Pp.48-62.

3) Sekhampu, J.T. & Niyimbanira, F. 2013. Analysis of the factors influencing household expenditure in a South African township. *International Business & Economics Research Journal.* 12(3), Pp.279-284.

4) Smith, B. and Diedrich, A., 2024. A systematic review of current progress in community based vulnerability assessments. *Regional Environmental Change*, *24*(1), pp.1-17.5) SINS. 2012. *Solomon Islands Household Income and Expenditure Survey National Analytical Report.* Solomon Islands National Statistics Office. Honiara, Solomon Islands. Pp.1-178.

6) Thathsarani, U.S. & Gunaratne, P.H.L. 2018. Constructing an index to measure the adaptive capacity to climate change in Sri Lanka. *Procedia Engineering.* 212, Pp.278-285.

F4: Income Satisfaction

Indicator Summary

Indicator: The extent to which a household is satisfied with their ability to purchase basic goods.

Indicator Description: Household income satisfaction level based on their ability to purchase basic goods required for survival (e.g., food, water, healthcare, energy and housing). Income satisfaction was measured

on a scale ranging from (1) very dissatisfied; (2) somewhat dissatisfied; (3) neither satisfied or dissatisfied; (4) somewhat satisfied; (5) very satisfied.

Measuring Unit: Level of satisfaction (5-point Likert Scale: 1) very dissatisfied – 5) very satisfied).

Contribution to Sensitivity

Summary: The higher a household's overall satisfaction with income, the greater their capacity to adapt to hazard exposure.

Justification: Objective income measures (e.g., FS2) assume a linear relationship between a greater level of income and a household's ability to purchase basic goods and satisfy essential needs (e.g., purchase food, water, healthcare etc.,) (Rojas 2008). Despite the importance of this metric, Camfield & Guillen-Royo (2009) have demonstrated that subjective income metrics (i.e., income satisfaction) can be of greater value in determining a household's overall sense of well-being and equality based on income. This is associated to variation in the amount of income required to acquire basic goods across different contexts (Ahmad et al. 2016; Fafchamps & Shilip 2008). Subjective income measures have therefore been argued to provide greater clarity in relation to evaluation the overall ability for household income to support access to basic goods (Camfield & Guillen-Royo 2009).

Indicator Method

Indicator Origin & Adaptations

Origins: Satisfaction with income is a key indicator applied in the Canterbury Wellbeing Index as a component of overall life satisfaction. The indicator is collected via a household survey question that asks respondents 'how well their household income supports them in meeting their everyday needs' (Canterbury Wellbeing Index 2023). Income satisfaction has been recognised as a key indicator of well-being in numerous studies and has become a commonly utilised indicator in relation to income security and life satisfaction (Kubiszewksi et al. 2018).

Adaptations: The wording of the household survey question reflecting income satisfaction was adapted during pilot testing to enhance clarity in the local context. Responses were separated into a 5-point Likert Scale ranging from 1) very dissatisfied to 5) very satisfied, reflecting the 5-point scale utilised in other financial capital LRP indicators. This also supported question simplicity, ease of use helped prevent respondent fatigue given the overall length of the household survey.

Data Collection Instrument

Household Survey Question: How satisfied are you with your ability to purchase the items your household needs to survive (e.g., food, water, healthcare, etc.,)?

**Data Type**	Ordinal

1. Input data reflecting household satisfaction within income ranging on a scale from (1) very dissatisfied (low adaptive capacity) to (5) very satisfied (high adaptive capacity).

Indicator Rankings

**Adaptive Capacity Rank**		**Definition**	**Description**
1	Very Low	Very dissatisfied	Household is very dissatisfied with their income.
2	Low	Somewhat dissatisfied	Household is somewhat dissatisfied with their income.
3	Medium	Neither satisfied nor dissatisfied	Household is neither satisfied nor dissatisfied with their income.
4	High	Somewhat satisfied	Household is somewhat satisfied with their income.
5	Very High	Very satisfied	Household is very satisfied with their income.

Key References

1) Ahmad, F.N., Mansor, M. & Paim, L. 2016. Income poverty and well-being among vulnerable households: A study in Malaysia. *Asian Journal of Social Sciences.* 12(2), Pp.1911-2023.
2) Camfield, L. & Guillen-Royo, M. 2001. Wants, needs and satisfaction: A comparative study in Thailand and Bangladesh. *Social Indicators Research.* 96. Pp.183-203.
3) Canterbury Wellbeing Index. 2023. *Satisfaction with Income.* Canterbury Wellbeing Index. New Zealand. Pp.1-7.
4) Fafchamps, M., & Shilpi, F. 2008. Subjective welfare, isolation, and relative consumption. *Journal of Development Economics.* 86, Pp. 43-60.
5) Kubiszewksi, I., Zakariyya, N. & Costanza, R. 2018. Objective and subjective indicators of life satisfaction in Australia: How well do people perceive what supports a good life? *Ecological Economics.* 154. Pp.361-372.
6) Rojas, M. 2008. Experienced Poverty and Income Poverty in Mexico: A Subjective Well-Being Approach. *World Development.* 36(6). Pp.1078-1093.
7) Susilo, E., Purwanti, P., Fattah, M., Qurrata, A.V. & Namaditya, S.B. 2021. Adaptive coping strategies towards seasonal change impacts: Indonesian small-scale fisherman household. *Heliyon.* 7, Pp.681-692.

F5: Access to Financial Services

**Indicator Summary**			
**Indicator:** Household access to key financial services including bank accounts, pensions and loans.			
**Indicator Description:** Household utilisation of financial services comprising (1) bank accounts (e.g., daily accounts, and savings accounts), (2) pensions (and associated accounts) and (3) loans.			
**Measuring Unit:** The number of financial services accessed by a household (e.g., how many of the following does a household presently utilise; 1) bank accounts; 2) pensions and 3) loans?).			
**Contribution to Adaptive Capacity**			
**Summary:** Household's with access to a range of financial services will have a greater capacity to adapt to hazard exposure.			
**Justification:** Financial exclusion (i.e., lack of access to financial services including bank accounts, credit, pensions or loans) has been evidenced to reduce adaptive capacity by aggravating poverty and driving financial isolation (Kamran & Uusitalo 2016). When a household experiences financial exclusion, their ability to respond to hazard exposure is significantly reduced through a number of mechanisms. These include: i) limited ability to build and maintain a financial safety net through savings, access to loans etc., which can be used to recover from shocks or stressors or in times emergency (e.g., healthcare costs or repairs) (Christian et al. 2019); ii) limited ability to invest in opportunities that require money including education and skills development that may increase adaptive capacity (Messy & Monticone 2016) and; iii) limited ability to invest in adaptive planning mechanisms that may reduce the potential impact of hazard exposure in the long-term (Kamran & Uusitalo 2016).			
**Indicator Method**			
**Indicator Origin & Adaptations**			
**Origins:** Access to financial services is applied as an indicator in many CBVA studies (Smith & Diedrich 2024). These studies measure access to a variety of services including bank accounts, pensions, loans, credit, insurance and extension services. Access is most commonly measured based on a household's present utilisation of these services (e.g., Aguilar 2021; Ho 2021).			
**Adaptations:** The types of financial services assessed in this approach were selected based on discussions with local experts (i.e., in-country civil society organisations). This led to the identification of i) bank accounts; ii) pensions and iii) loans as the most important types of services in the local context. To create a consolidated indicator for access to financial services, a 4-point scale was developed whereby no access = very low adaptive capacity, and access to all three service types = very high adaptive capacity.			
**Data Collection Instrument**			
**Household Survey Question:** Does your household currently use any of the following services: 1) bank accounts; ii) pensions (and associated accounts) or; iii) loans?			
**Data Type**	Integer		
1. Input household access to financial service types (e.g., 1) bank accounts; 2) pensions; 3) loans).<br>2. Rank access to financial services based on the following scores, whereby no access = (1) very low adaptive capacity and full access = (4) very high adaptive capacity.			
**Indicator Rankings**			
**Adaptive Capacity Rank**		**Definition**	**Description**
1	Very Low	No Access	Household does not have access to any financial services
2	Low	Single Access	Household has access to a single financial service.
3	Medium	Dual Access	Household has access to two financial services.
4	High	Full Access	Household has access to all three financial services.
**Key References**			
1) Aguilar, M.H.C., Altoveros, N.C., Borromeo, H.T., Dayo, F.H.M. & Koohafkan, P. 2021. Traditional rice-based agroecosystem in Kiangan, Ifugao, Philippines: drivers of change, resilience and potential trajectories. *Agroecology and Sustainable Food Systems.* 45, 2. Pp.296-316.<br>2) Christian, P., Kandpal, E., Palaniswamy, N. & Rao, V. 2019. Safety nets and natural disaster mitigation: evidence from cyclone Phailin in Odisha. *Climatic Change.* 153. Pp.141-164.<br>3) Ho, T.D., Kuwornu, J.K. & Tsusaka, T.W. 2021. Factors influencing smallholder rice farmers vulnerability to climate change and variability in the Mekong Delta region of Vietnam. *The European Journal of Development Research.* Pp.1-31.			

4) Kamran, S. & Uusitalo, O. 2016. Vulnerability of the unbanked: evidence from a developing country. *International Journal of Consumer Studies.* 40. Pp.400-409.
5) Messy, A.F. & Monticone, C. 2016. Financial education policies in Asia and the Pacific. *OECD Working Paper on Finance, Insurance and Private Pensions.* No. 40. OECD Publishing. Pp.1-66.
6) Smith, B. and Diedrich, A., 2024. A systematic review of current progress in community based vulnerability assessments. *Regional Environmental Change*, *24*(1), pp.1-17.

Social Capital

S1: Social Networks

Indicator Summary

Indicator: The number of social relationships a household can turn to for support in times of need.

Indicator Description: The number of individuals that a household can turn to for support if they need access to basic livelihood outcomes such as food, water, shelter, energy or healthcare.

Measuring Unit: Number of individuals within a households social network.

Contribution to Adaptive Capacity

Summary: The greater the number of individuals a household can turn to for support in times of need, the greater their capacity to adapt to hazard exposure.

Justification: The informal and formal social relationships (i.e., social networks) held by a household are a key component of adaptive capacity (Cinner et al. 2018). Well-connected households are capable of accessing their social relationships to obtain information, resources, support and opportunities to enhance their adaptive potential (Barnes et al. 2017). For example, information and knowledge related to hazard exposure is commonly shared across social networks, increasing the ability for a household to make appropriate adaptive decisions (Fazey et al. 2014). Social networks are also known to facilitate resource sharing and support in times of need, with individuals with strong social relationships more likely to share food, physical assets, money etc., (Islam & Nguyen 2018). Communities with strong social networks are also more likely to engage in collaborative adaptation actions, which often act as the best adaptive method when shocks and stressors are homogenous across the local environment (Nunan et al. 2018). Involvement in community-based actions can additionally enhance wellbeing by increasing a household's sense of belonging and cohesion with neighbours (Lai et al. 2021), whilst providing greater access to community-based opportunities such as livelihood activities and market access (Nunan et al. 2018).

Indicator Method

Indicator Origin & Adaptations

Origins: Information on the number of social relationships a household relies on for support has been evidenced to provide basic empirical insight into social network responses, and correspondingly adaptive capacity (Misra et al. 2017). Counting the number of social relationships has been used as an indicator in many CBVA studies (Smith & Diedrich 2024). Evidence suggests that most households have a social network of close friends and family numbering between 5-12 individuals (Dunbar & Spoors 1995). As such, this was deemed an appropriate question metric for the LRP social network indicator.

Adaptations: n/a

Data Collection Instrument

Household Survey Question: If you suddenly needed access to water, food, shelter, energy or healthcare, how many people that are not members of your household could you turn to for help? 1) no one; 2) 1-2 people; 3) 3-4 people; 4) >5 people; 5) >10 people.

**Data Type**	Ordinal

1. Input responses from the household survey reflecting the number of social relationships held by a household.
2. Rank the number of social relationships according to the following ranking categories, whereby 1) no social relationships = (1) very low adaptive capacity and 5) >10 social relationships = (5) very high adaptive capacity.

Indicator Rankings

**Adaptive Capacity Rank**		**Definition**	**Description**
1	Very Low	No One	A household has no one to turn to in times of need.
2	Low	1-2 People	A household has 1-2 people to turn to in times of need.

3	Medium	3-4 People	A household has 3-4 people to turn to in times of need.
4	High	>5 People	A household has >5 people to turn to in times of need.
5	Very High	>10 People	A household has >10 people to turn to in times of need.

Key References

1) Barnes, L.M., Wang, P., Cinner, E.J., Graham, A.J.N., Guerrero, M.A., Jasny, L., Lau, J., Sutcliffe, R.S. & Zamborain-Masno, J. 2020. Social determinants of adaptive and transformative responses to climate change. *Nature Climate Change.* 10, Pp.823-828.
2) Cinner, E.J. & Barnes, L.M. 2019. Social dimensions of resilience in social-ecological systems. *One Earth.* 1, Pp.51-56.
3) Dunbar, M.I.R. & Spoors, M. 1995. Social networks, support cliques and kinship. *Human Nature.* 6. Pp.273-290.
4) Fazey, J., Bunse, L., Msika, J., Pinke, M., Preedy, K., Evely, C.A., Lambert, E., Hastings, E., Morris, S. & Reed, S.M. 2014. Evaluating knowledge exchange in interdisciplinary and multi-stakeholder research. *Global Environmental Change.* 25, Pp.204-220.
5) Islam, A. & Nguyen, C. 2018. Do networks matter after a natural disaster? A study of resource sharing within an informal network after Cyclone Aila. *Journal of Environmental Economics and Management.* 90. Pp.249-268.
6) Lai, S., Zhou, Y. & Yuan, Y. 2021. Associations between community cohesion and subjective wellbeing of the elderly in Guangzhou China – A cross sectional study based on the structural equation model. *International Journal of Environmental Research and Public Health.* 18, Pp.953-960.
7) Misra, S., Goswami, R., Mondal, T. & Jana, R. 2017. Social networks in the context of community response to disaster: Study of a cyclone affected community in Coastal West Bengal, India. *International Journal of Disaster Risk Reduction.* 22, Pp.28-296.
8) Nunan, F., Cepic, D., Mbilingi, B., Odongkara, K., Yongo, E., Owili, M., Salehe, M., Mlahagaw, E. & Onyango, P. 2018. Community cohesion: Social and economic ties in the personal networks of fisherfolk. *Society & Natural Resources.* 31(3). Pp.306-319.
9) Smith, B. and Diedrich, A., 2024. A systematic review of current progress in community based vulnerability assessments. *Regional Environmental Change*, *24*(1), pp.1-17.

S2: Inclusion in Decision Making

Indicator Summary

Indicator: The extent of household satisfaction with its inclusion in community level decision making.

Indicator Description: The extent to which a household is satisfied with their role in community level decision making, varying from i) strongly disagree; ii) disagree; iii) neither agree nor disagree; iv) agree; v) strongly agree.

Measuring Unit: Extent of satisfaction with decision making capacity. (5-point Likert Scale: Strongly Disagree – Strongly Agree).

Contribution to Adaptive Capacity

Summary: Households with higher levels of satisfaction with their inclusion in community decision making will have a greater capacity to adapt to hazard exposure.

Justification: Household ability to be involved in local scale decision making has been shown to have an overall positive influence on social capital and adaptive capacity (Ensor et al. 2009). This relationship occurs through a number of mechanisms, whereby involvement in decision making causes individuals with limited capacity to implement adaptive actions, or make decisions for themselves, to experience increased opportunity to benefit from applied management actions when involved in decision making activities (Smit & Wandel 2006). Involvement in decision making also supports the development of ‘fair adaptation’ that provides equal benefit to community members with different socioeconomic characteristics (Paavola & Adger 2006); and increases the likelihood these adaptation strategies will be adopted, given their increased reflection of local desires, wants, and aspirations (Patterson et al. 2009). Finally, inclusion in decision making has been shown to drive an increase in trust of community members and leaders, further contributing to social capital and adaptive capacity (Ensor et al. 2015).

Indicator Method

Indicator Origin & Adaptations

Origins: Decision making capacity is a commonly utilised indicator in studies applying the Sustainable Livelihoods Framework (SLF) (Smith & Diedrich 2024), measured via Likert scale questions that obtain information on a household’s perceived level of involvement (e.g., Arthur et al. 2020; Bisseleua et al. 2018; Freduah et al. 2018; Lin et al. 2019).

Adaptations: n/a

Data Collection Instrument

Household Survey Question: Are you happy with your level of involvement with decisions that are made in your community? 1) strongly disagree; 2) disagree; 3) neither agree nor disagree; 4) agree; 5) strongly agree.			
**Data Type**	Ordinal		
1. Input responses from household survey regarding perceived levels of satisfaction with involvement in community decision making where 1) strongly disagree = (1) a household is very dissatisfied with their level of involvement in decision making equates to very low adaptive capacity and, to 5) strongly agree = (5) household is very satisfied with their level of involvement in decision making equates to very high adaptive capacity.			
**Indicator Rankings**			
**Adaptive Capacity Rank**		**Definition**	**Description**
1	Very Low	Very Dissatisfied	Household is very dissatisfied with their perceived level of involvement in community decision making.
2	Low	Somewhat Dissatisfied	Household is somewhat dissatisfied with their perceived level of involvement in community decision making.
3	Medium	Neither Satisfied Nor Dissatisfied	Household is neither satisfied nor dissatisfied with their perceived level of involvement in community decision making.
4	High	Somewhat Satisfied	Household is somewhat satisfied with their perceived level of involvement in community decision making.
5	Very High	Very Satisfied	Household is very satisfied with their perceived level of involvement in community decision making.

Key References

1) Arthur, L.J., Murray, G., Rollins, R., Dearden, R. & Stahl, A. 2020. Differential impacts of dam construction on livelihoods in Ghana. *African Geographical Review.* 39(3). Pp.267-281.
2) Bisseleua, B.H.D., Idrissou, L., Olurotimi, P., Ogunniyi, A., Mignouna, D. & Bamire, A.S. Multi-stakeholder process strengthens agricultural innovations and sustainable livelihoods of farmers in Southern Nigeria. *The Journal of Agricultural Education and Extension.* 24(1). Pp.29-49.
3) Ensor, E.J., Park, S.E., Hoddy, E.T. & Ratner, B.D. 2015. A rights-based perspective on adaptive capacity. *Global Environmental Change.* 31(1), pp.38-49.
4) Freduah, G., Fidelman, P. & Smith, F.T. 2018. Adaptive capacity of small-scale coastal fishers to climate and non-climate stressors in the Western region of Ghana. *The Geographical Journal.* 185. Pp.96-110.
5) Lin, T., Htun, T.K., Gritten, D. & Martin, R.A. 2019. The contribution of community forestry to climate change adaptive capacity in tropical dry forests: lessons from Myanmar. *International Forestry Review.* 21(3). Pp.324-342.
6) Paavola, J. & Adger, W.N. 2006. Fair adaptation to climate change. *Ecological Economics.* 1, Pp.594-609.
7) Patterson, O., Weil, F. & Patel, K. 2010. The role of community in disaster response: Conceptual models. *Population Research and Policy Review.* 29, Pp.127-141.
8) Smit, B. & Wandel, J. 2006. Adaptation, adaptive capacity and vulnerability. *Global Environmental Change – Human and Policy Dimensions.* 16(3), Pp.282-292.
9) Smith, B. and Diedrich, A., 2024. A systematic review of current progress in community based vulnerability assessments. *Regional Environmental Change*, *24*(1), pp.1-17.

S3: Local Institutional Membership

Indicator Summary

Indicator: Household membership in local institutions (e.g., groups, organisations and associations).

Indicator Description: The number of local institutional (e.g., groups, organisations and associations) memberships held by a household. During household surveys, community members were asked to list their institutional memberships. These were later cross-checked against a complete list of community-based institutions obtained during key informant interviews with community leaders. A full list of institutional types was developed for each community, and the number of memberships per household summed. This number was then used to reflect adaptive capacity, whereby 0 memberships = low adaptive capacity and >5 memberships = high adaptive capacity.

Measuring Unit: The number of local groups, organisations and associations a household is a member of.

Contribution to Adaptive Capacity

Summary: The greater the number of institutions a household is a member of, the greater their capacity to adapt to hazard exposure.

Justification: In the context of the Sustainable Livelihoods Framework institutional access refers to the organisations, associations and groups that exist to govern local processes, and reflect systems of rules, regulations and norms (DFID 1999). Yaro et al. (2014) provide evidence to support the positive association between institutional membership and adaptive capacity. This highlights the role institutions play in influencing social interactions between members (through the legitimisation of rules, norms and practices), whilst also enhancing members ability to access shared resources to enhance adaptive capacity, including natural resources and knowledge (Agrawal et al 2008; Yaro et al. 2014). Positive interactions have also been seen between institutional membership and a household's ability to contribute to local scale decision making, assessed as an additional component of social capital in this approach (Malakar 2013).

Indicator Method

Indicator Origin & Adaptations

Origins: Membership to local organisations, associations and groups is a common indicator applied within CBVAs to assess household involvement in local-scale institutions (Smith & Diedrich 2024). Within these studies, membership is normally measured by i) obtaining an understanding of the institutions a household is member of based on an open-ended response question; or ii) listing all available local institutions and asking a household to check off those they are member to (e.g., Gai 2018; Kuuwill et al. 2022; Mallick 2020; Pour 2018). For this indicator the first approach was utilised, with households asked to free list their institutional memberships.

Adaptations: The ranking categories for this indicator were developed following data collection. This was based on significant contextual variation in the number of institutions households are a member of. In the context of this approach, no household was member to more than 4 groups, organisations or associations, as such, a 0-4 range was dictated for the adaptive capacity ranking scale.

Data Collection Instrument

Household Survey Question: Please list any community-based (i.e., local) groups, organisations, or associations that your household belongs to.

Data Type	Text

1. List the total number of local institutions available within a community based on qualitative information obtained from key informant interviews, cross-checked with a full list of institutions obtained during household surveys.
2. Sum the number of institutions a household is a member of within a community (and cross-check this list against the full list of available institutions developed in step 1).
3. Rank household membership to local institutions where very low membership (and adaptive capacity) = 0 institutions, and very high membership (and adaptive capacity) = >5 institutions.

Indicator Rankings

Adaptive Capacity Rank		Definition	Description
1	Very Low	0 Memberships	Household is not member to local institutions.
2	Low	1 Membership	Household is member to a single local institutions.
3	Medium	2 Memberships	Household is member to several local institutions.
4	High	3 Memberships	Household is member to a few local institutions.
5	Very High	4 Memberships	Household is member to many local institutions.

Key References

1) Agrawal, A., Chhatre, A. & Hardin, R. 2008. Changing governance of the world's forests. *Science.* 320, Pp.1460-1472.
2) DFID. 1999. *Sustainable Livelihood Guidance Sheets.* Department for International Development. London, United Kingdom. Pp.1-150.
3) Gai, A.M., Soewarni, I. & Sir, M.M. 2018. *The concept of community poverty reduction in coastal area of Surabaya based on sustainable livelihood approach*. In. IOP Conference series: Earth and Environmental Science. 137(1). Pp.120-129.
4) Kuuwill, A., Kimensgi, N.J. & Campion, B.B. 2022. Pandemic induced shocks and shifts in forest based livelihoods strategies: learning from the Covid-19 in the Bia West district of Ghana. *Environmental Research Letters.* 34. Pp.640-633.
5) Malakar, Y. 2014. Increasing adaptive capacity: What is the role of local institutions? *Risk, Hazards & Crisis in Public Policy.* 60, Pp.1944-1968.
6) Mallick, B., Sultana, Z. & Bennett, C.M. 2020. How do sustainable livelihoods influence environmental (non-) migration aspirations? *Applied Geography.* 124. Pp.102-132.
7) Ostrom, E. 2000. Private and common property rights. *Centre for the Study of Institutions, Population and Environmental Change.* Indiana University.

8) Pour, M.D., Barati, A.A., Azadi, H. & Scheffran, J. 2018. Revealing the role of livelihood assets in livelihood strategies: Towards enhancing conservation and livelihood development in the Hara Biosphere Reserve. *Ecological Indicators.* 94. Pp.336-347.
9) Smith, B. and Diedrich, A., 2024. A systematic review of current progress in community based vulnerability assessments. *Regional Environmental Change*, *24*(1), pp.1-17.
10) Yaro, A.J., Teye, J. & Bawakyillenuo, S. 2015. Local institutions and adaptive capacity to climate change/variability in the northern savannah of Ghana. *Climate and Development.* 7(3), Pp.235-245.

S4: Satisfaction with Leadership

Indicator Summary

Indicator: The extent to which a household is satisfied with the leadership in their community.

Indicator Description: The extent to which household members are satisfied with the leadership in their community, ranging from (1) strongly disagree; (2) disagree; (3) neither agree nor disagree; (4) agree; (5) strongly agree.

Measuring Unit: Satisfaction with leadership. (5-point Likert Scale: Strongly Disagree – Strongly Agree).

Contribution to Adaptive Capacity

Summary: The greater a household's satisfaction with community leadership, the greater their capacity to adapt to hazard exposure.

Justification: Diedrich et al. (2019) found satisfaction with leadership to be the most influential aspect of social capital in relation to small scale fishing communities in Papua New Guinea. This was attributed to the role leadership plays in achieving positive success in natural resource management (Diedrich et al. 2017). Leadership satisfaction has also been found to have a direct correlation with the equitable distribution of economic benefits linked to natural resource use, and general access to resources (Banks 2008). In association to adaptive capacity, higher income levels and access to natural resources increase a household's ability to respond to hazard exposure (Jin et al. 2016). When community members are more satisfied with leadership, they have also been found to increase their support of community-level adaptation (Guitierrez et al. 2011).

Indicator Method

Indicator Origin & Adaptations

Origins: Satisfaction with leadership has been applied in many CBVAs as a component of social capital (e.g., Diedrich et al. 2019; Islam et al. 2019; Rampengan et al. 2016). Studies generally utilise a basic household survey question comprising 'are you satisfied with the leadership in your village' to measure this indicator.

Adaptations: The household survey question used the word 'happy' to assess satisfaction with leadership. This decision was based on local expert knowledge and survey pilot testing, with 'happy' deemed more interpretable in the local context.

Data Collection Instrument

Household Survey Question: How much do you agree with the following statement? I am happy with the leadership in my community. (1) Strongly disagree; (2) Disagree; (3) Neither Agree nor Disagree; (4) Agree; (5) Strongly Agree.

Data Type: Ordinal

1. Input responses from household survey reflecting if a household is satisfied with the leadership in their community varying from (1) strongly disagree (very low adaptive capacity) to (5) strongly agree (very high adaptive capacity).

Indicator Rankings

Adaptive Capacity Rank		Definition	Description
1	Very Low	Very Dissatisfied	Household is very dissatisfied with community leadership.
2	Low	Somewhat Dissatisfied	Household is somewhat dissatisfied with community leadership.
3	Medium	Neither Satisfied nor Dissatisfied	Household is neither satisfied nor dissatisfied with community leadership.
4	High	Somewhat Satisfied	Household is somewhat satisfied with community leadership.
5	Very High	Very Satisfied	Household is very satisfied with community leadership.

Key References

1) Banks, G. 2008. Understanding 'resource' conflicts in Papua New Guinea. *Asia-Pacific Viewpoint.* 49. Pp.23-34.
2) Diedrich, A., Stoeckl, N., Gurney, G., Esparon, M. & Pollnac, R. 2017. Social capital as a key determinant of perceived benefits of community-based marine protected areas. *Conservation Biology.* 31. Pp.311-321.

3) Diedrich, A., Benham, C., Pandihau, L. & Sheaves, M. 2019. Social capital plays a central role in transitions to sportfishing tourism in small-scale fishing communities in Papua New Guinea. *Ambio.* 48. Pp.385-396.
4) Guiterrez, N.L., Hilborn, R. & Defero, O. 2011. Leadership, social capital and incentives promote successful fisheries. *Nature.* 470. Pp..386-389.
5) Islam, K., Nath, K.T., Jashumuddin, M. & Rahman, F. 2019. Forest dependency, co-management and improvement of people's livelihood capital: Evidence from Chunati Wildlife Sanctuary, Bangladesh. *Environmental Development.* 32. Pp.100-123.
6) Jin, J., Wang, W. & Wang, X. 2016. Adapting agriculture to the drought hazard in rural China: household strategies and determinants. *Natural Hazards.* 82, Pp.1609-1619.
7) Rampengan, M.F.M., Law, L., Gaillard, C.J., Boedhihartono, K.A. & Sayer, J. 2016. Engaging communities in managing multiple hazards: Reflections from small islands in North Sulawesi, Indonesia. *Singapore Journal of Tropical Geography.* 37. Pp.249-267.

S5: Trust

Indicator Summary

Indicator: The level of trust a household has in community members.

Indicator Description: The extent to which household members trust the people in their community, ranging from (1) strongly disagree; (2) disagree; (3) neither agree nor disagree; (4) agree; (5) strongly agree.

Measuring Unit: Extent of trust (5-point Likert Scale: Strongly Disagree – Strongly Agree).

Contribution to Adaptive Capacity

Summary: The greater a household's level of trust towards fellow community members, the greater their capacity to adapt to hazard exposure.

Justification: Trust in community members is an important component of adaptive capacity (Folke et al. 2005). High levels of trust are evidenced to increase resource exchange between community members, stimulate innovative thinking, and increase the overall success of community-based governance activities (Pelling & Hugh 2005). These benefits are attributed to the role trust plays in encouraging community members to work together to achieve shared objectives and feel accountability in relation to the management of a community and its resources (Agrawal & Chhatre 2006).

Indicator Method

Indicator Origin & Adaptations

Origins: Trust is a complex concept, which if assessed comprehensively encompasses a broad range of indicators spanning trust in individuals, place, leadership etc., (Di Napoli et al. 2019). In the context of this approach, a comprehensive assessment of trust was unfeasible given potential respondent fatigue and time-availability in relation to household survey questions. As such, a common indicator to assess trust applied within CBVAs was selected, which primarily related to household trust in community members (Smith, B. & Diedrich, A. 2023). Using a statement-based question to assess trust is a common household survey technique. In this context, respondents are asked to respond to a statement such as 'I trust the members of my community' using a Likert-scale response system ranging from strongly agree to strongly disagree (Yamagishi & Yamagishi 1994). This technique was used to assess trust in the LRP index approach.

Adaptations: n/a

Data Collection Instrument

Household Survey Question: How much do you agree with the following statement? I trust the people in my community. (1) strongly disagree; (2) disagree; (3) neither agree nor disagree; (4) agree; (5) strongly agree.

**Data Type**	Ordinal

1. Input responses from household survey reflecting if a household trusts the people in their community varying from (1) strongly disagree (very low adaptive capacity) to (5) strongly agree (very high adaptive capacity).

Indicator Rankings

**Adaptive Capacity Rank**		**Definition**	**Description**
1	Very Low	Strongly Disagree	Household strongly disagrees with the statement 'I trust the people in my community'.
2	Low	Disagree	Household disagrees with the statement 'I trust the people in my community'.
3	Medium	Neither Agree nor Disagree	Household neither agrees nor disagrees with the statement 'I trust the people in my community'.

4	High	Agree	Household agrees with the statement 'I trust the people in my community'.
5	Very High	Strongly Agrees	Household strongly agrees with the statement 'I trust the people in my community'.

Key References

1) Agrawal, A. & Chhatre, A. 2006. Explaining success on the commons: Community Forest governance in the Indian Himalaya. *World Development.* 34(1). Pp.149-166.
2) Di Napoli, I., Dolce, P. & Arcidiacono, C. 2019. Community Trust: A social indicator related to community engagement. *Social Indicators Research.* 145(1). Pp.551-579.
3) Folke, C., Hahn, T., Olssen, P. & Norberg, J. 2005. Adaptive governance of social-ecological systems. *Annual Review of Environmental Resources.* 30. Pp.441-473.
4) Smith, B. and Diedrich, A., 2024. A systematic review of current progress in community based vulnerability assessments. *Regional Environmental Change*, *24*(1), pp.1-17.
5) Pelling, M. & Hugh, C. 2005. Understanding adaptation: what can social capital offer assessments of adaptive capacity? *Global Environmental Change.* 15(4). Pp.308-319.
6) Yamagishi, T. & Yamagishi, M. 1994. Trust and commitment in the United States and Japan. *Motivation and Emotion.* 18. Pp.129-166.

S6: Collective Action

Indicator Summary

Indicator: The extent of household participation in community-based activities.

Indicator Description: The extent to which a household participates in community-based activities ranging from; 1) never; 2) rarely; 3) sometimes; 4) frequently and 5) all the time.

Measuring Unit: The extent (e.g., rated amount of time) a household participates in community activities. (5-point Likert Scale: (1) Never – (5) All the time).

Contribution to Adaptive Capacity

Summary: Households that exhibit greater involvement in community-based activities will have a greater capacity to adapt to hazard exposure.

Justification: Collective action refers to the steps taken by a group of people (e.g., a community) that have chosen to engage in a common action or activity in pursuit of a shared goal or interest (Matta & Alavalpati 2006). Collective action has been shown to positively contribute to adaptive capacity by i) supporting the establishment and strengthening of social networks, ii) improving access to local financial resources, iii) supporting individual and collective empowerment and, iv) offering a forum for community members to communicate and resolve problems (Adger 2003; Ireland & Thomella 2011).

Indicator Method

Indicator Origin & Adaptations

Origins: At the household level, collective action is commonly represented by the extent of a household's involvement in community-based activities (Meinzen-Dick et al. 2004). The use of Likert-based measures for this indicator provides households with a structured self-assessment of their level of participation in community-based activities, in a manner that is straightforward and easy to understand. Measurement of household participation using a Likert Scale has been used to assess collective action in many previous vulnerability assessments (e.g., Brown 2018; Kaskoya 2017; Phan 2019).

Adaptations: n/a

Data Collection Instrument

Household Survey Question: In the past year, did anyone in your household participate in any community activities? (1) never; (2) rarely; (3) sometimes; (4) frequently; (5) all the time.

**Data Type**	Ordinal

1. Input responses from household survey question onto the standardised indicator ranking scale whereby (1) never = very low adaptive capacity and (5) all the time = very high adaptive capacity.

Indicator Rankings

**Adaptive Capacity Rank**		**Definition**	**Description**
1	Very Low	Never	Household members never participate in community activities.
2	Low	Rarely	Household members rarely participate in community activities.
3	Medium	Sometimes	Household members sometimes participate in community activities.
4	High	Frequently	Household members frequently participate in community activities.

5	Very High	All the Time	Household members always participate in community activities.

Key References

1) Adger, N. 2003. Social capital, collective action, and adaptation to climate change. *Economic Geography.* 74(4), Pp.387-404.
2) Brown, P.R., Tuan, V.V., Nhan, D.K., Dung, L.C. & Ward, J. 2018. Influence of livelihoods on climate change adaptation for smallholder farmers in the Mekong Delta, Vietnam. *International Journal of Agricultural Sustainability.* 16(3). Pp.255-271.
3) Ireland, P. & Thomalla, F. 2011. The role of collective action in enhancing communities' adaptive capacity to environmental risk: An exploration of two case studies from Asia. *PLoS Currents.* 26(3), Pp.1-16.
4) Kaskoya, H., Mohammed, A. & Inoue, M. 2017. Impact of community forest program in protection forest on livelihood outcomes: A case study of Lampung Province, Indonesia. *Journal of Sustainable Forestry.* 36(3). Pp.250-263.
5) Matta, J.R. & Alavalapati, R.R.J. 2006. Perceptions of collective action and its success in community based natural resource management: An empirical analysis. *Forest Policy and Economics.* 9, Pp.274-284.
6) Meinzen-Dick, R., DiGregorio, M. & McCarthy, N. 2004. Methods for studying collective action in rural development. *Agricultural Systems.* 82(3), Pp.197-214.
7) Phan, L.T., Jou, S.C. & Lin, J.H. 2019. Gender inequality and adaptive capacity: The role of social capital on the impacts of climate change in Vietnam. *Sustainability.* 11(5), Pp.125-137.

S7: Fair Access to Livelihood Opportunities

Indicator Summary

Indicator: Household perception of fair access to livelihood opportunities within their community.

Indicator Description: The extent to which a household believes there is fair access to new economic opportunities within their community, ranging from (1) strongly disagree; (2) disagree; (3) neither agree nor disagree; (4) agree and (5) strongly agree.

Measuring Unit: Perception of fair access (5-point Likert Scale: Strongly Disagree – Strongly Agree).

Contribution to Adaptive Capacity

Summary: The more a household believes there is fair access to new economic opportunities within their community, the greater their capacity to adapt to hazard exposure.

Justification: Fair access to resources has been deemed an important component of adaptive capacity, supporting a household's ability to build preparedness and develop adaptation strategies to overcome hazard exposure (Cafer et al. 2019). Fairness-based indicators provide an understanding of marginalisation within a community, where access to resources (such as income) may be limited (Williamson et al. 2012). Research has found perceptions of fairness to be representative of resource access, with low perceptions (i.e., limited fair access) reflective of corruption, marginalisation, and poor community relationships (Bennett et al. 2014). Unfair access to income can reduce household adaptive capacity by i) limiting resource access, ii) increasing dependence on existing resources that may be degraded, and iii) restricting access to infrastructure and services required to achieve livelihood outcomes (e.g., food, water, housing, energy, income and healthcare security) (Jones et al. 2022; Susilo et al. 2021). Whilst there is no one measure of fairness, access to income making opportunities (e.g., livelihood activities) was selected for this approach to reflect the importance of alternate livelihood activities as a component of adaptive capacity. This stems from the major adaptive opportunity access to alternative livelihoods has played in many rural environments, supporting local development and reducing impacts from hazard exposure (e.g., Cinner et al. 2018; Thulstrup 2015).

Indicator Method

Indicator Origin & Adaptations

Origins: Fairness based indicators are not a common component of CBVAs (Smith & Diedrich 2024). However, there is significant evidence to support their value for inclusion as a component of adaptive capacity (Cafer et al. 2019; Williamson et al. 2012). Where measures of fairness are included in household surveys, they are commonly related to perceived levels of resource access (e.g., Braun et al. 2008; Howley et al. 2008; Sharkey et al. 2010). This justified the use of a perception-based metric to quantify fairness within the LRP index.

Adaptations: This indicator was developed based on previous household survey questions that measure fairness based on Likert scale responses (e.g., Braun et al. 2008; Howley et al. 2008; Sharkey et al. 2010). Adaptations were made to suit the specific information required for this indicator, with the phrase 'access to new economic opportunities' selected following discussions with local experts.

Data Collection Instrument

Household Survey Question: How much do you agree with the following statement? Access to new economic opportunities within my community is fair. (1) strongly disagree; (2) disagree; (3) neither agree nor disagree; (4) agree; (5) strongly agree.			
**Data Type**		Ordinal	
1. Input responses from household survey whereby (1) strongly disagree that access to new economic opportunities is fair = very low adaptive capacity, to (5) strongly agree that access to new economic opportunities is fair = very high adaptive capacity.			
**Indicator Rankings**			
**Adaptive Capacity Rank**		**Definition**	**Description**
1	Very Low	Strongly Disagree	Household strongly disagrees that resource access is fair.
2	Low	Disagree	Household disagrees that resource access is fair.
3	Medium	Neither Agree nor Disagree	Household neither agrees nor disagrees that resource access is fair.
4	High	Agree	Household agrees that resource access is fair.
5	Very High	Strongly Agree	Household strongly agrees that resource access is fair.

Key References

1. Bennett, J.N., Dearden, P., Murray, G. & Kadfak, A. 2014 The capacity to adapt? Communities in a changing climate, environment, and economy on the northern Andaman coast of Thailand. *Ecology & Society.* 19(2), Pp.1-21.
2. Braun, M., Lewin-Epstein, N., Steir, H. & Baumgartner, K.M. 2008. Perceived equity in the gendered division of household labour. *Journal of Marriage and Family.* 70(5). Pp.1145-1156.
3. Cafer, A., Green, J. & Goreham, G. 2019. A community resilience framework for community development practitioners building equity and adaptive capacity. *Community Development.* 50, Pp.201-216.
4. Cinner. E.J., Adger, W.N., Allison, H.E., Barnes, L.M., Brown, K., Cohen, J.P., Gelcich, S., Hicks, C.C., Hughes, P.T., Lau, J., Marhsall, A.N. & Morrison, H.T. 2018. Building adaptive capacity to climate change in tropical coastal communities. *Nature Climate Change.* 8. Pp.117-123.
5. Howley. P., Scott, M. & Redmond, D. 2008. Sustainability versus liveability: an investigation of neighbourhood satisfaction. *Journal of Environmental Planning and Management.* 52(6). Pp.847-864.
6. Jones, L.H.B., Unsworth, F.K.R., Nordlund, M.L., Elkof, S.J., Ambo-Rappe, R., Carly, F., Jiddawi, S.N., Nafie, A.Y., Udagedara, S. & Cullen-Unsworth. 2022. Dependence on seagrass fisheries governed by household income and adaptive capacity. *Ocean & Coastal Management.* 225, Pp.106-126.
7. Susilo, E., Purwanti, P., Fattah, M., Qurrata, A.V. & Namaditya, S.B. 2021. Adaptive coping strategies towards seasonal change impacts: Indonesian small-scale fisherman household. *Heliyon.* 7, Pp.681-692.
8. Sharkey, R.J., Johnson, M.C. & Dean, R.W. 2010. Food access and perceptions of the community and household food environment as correlates of fruit and vegetable intake among rural seniors. *BMC Geriatrics.* 32(10). Pp.32-41.
9. Smith, B. and Diedrich, A., 2024. A systematic review of current progress in community based vulnerability assessments. *Regional Environmental Change*, *24*(1), pp.1-17.
10. Thulstrup, W.A., 2015. Livelihood resilience and adaptive capacity: Tracing changes in household access to capital in Central Vietnam. *World Development.* 74. Pp.352-362.
11. Williamson, T., Hesseln. H. & Johnston, M. 2012. Adaptive capacity deficits and adaptive capacity of economic systems in climate change vulnerability assessment. *Forest Policy and Economics.* 1, Pp,160-166.

Physical Capital

P1: Access to Livelihood Assets

**Indicator Summary**
**Indicator:** Household access to key assets required to support livelihoods.
**Indicator Description:** Household access to key goods and infrastructural assets that are required to support local livelihoods. The asset types included in this indicator were developed in coordination with a local civil society organisation in Solomon Islands and comprise: i) generator; ii) fridge or freezer; iii) boat, canoe, OBM, or other forms of transport; iv) communication devices including mobile phones or radios, v) the internet; vi) land for gardening and vii) access to equipment types required for agriculture/gardening, fishing and livestock rearing. This indicator reflects the percentage access a household has to different assets based on their relative importance to livelihoods. For example, access to assets of higher relative importance will give households a higher overall indicator score, compared to households that only have access to assets deemed of relatively low importance.
**Measuring Unit:** Number of assets owned by a household.

Contribution to Adaptive Capacity

Summary: Household's with greater access to livelihoods-based assets will have greater capacity to adapt to hazard exposure.

Justification: Access to livelihoods-based goods and infrastructure plays a key role in influencing adaptive capacity, directly influencing a household's ability to conduct livelihood activities and respond to shock and stressor events. For example, a household that lacks access to transport may be incapable of accessing resources and services such as healthcare, food markets, etc., (Daramola et al. 2016). A similar interaction occurs in relation to household access to communication devices, which can support resource and service access, whilst additionally acting as an early warning system to advise households of upcoming hazard events (Thatsarani & Gunaratne 2018). Access to generators, fridges and gardening land have a direct role in a household's ability to produce and store food in the short and long-term (Daramola et al. 2016). Access to livelihoods-based equipment ensure a steady supply of resources, whilst also enabling households to adapt to cope with periods of environmental/resource instability (Blakie & Wisner 2014). Access to livelihood equipment can also support income stability, by reducing dependence on externally sourced equipment (Li et al. 2020).

Indicator Method

Indicator Origin & Adaptations

Origins: Access to goods and infrastructure required to support livelihoods is a widely applied indicator in many CBVAs (Smith & Diedrich 2024). These studies generally assess if a household has access (based on a yes or no response) to infrastructure (e.g., transport, communication devices etc., (e.g., Becker 2017; Fischer 2021; Rampengan 2016)) and goods (e.g., agricultural (e.g., Azami & Shanazi 2020) and fisheries (e.g., Brown 2018) based equipment).

Adaptations: The LRP assessed household access to key infrastructure and goods deemed important to livelihoods in the local context of Solomon Islands. This was based on expert knowledge from a local civil society organisation. For livelihoods-based equipment, activity types followed the major activities occurring within partner communities (i.e., farming, kitchen gardening, fishing and livestock production). The LRP index used an open-ended question format to enable households to free list the equipment and tools owned, ensuring the full range of items were captured within responses.

Data Collection Instrument

Household Survey Question: Does your household have access to any of the following items? i) generator; ii) fridge or freezer; iii) boat, canoe, OBM or other forms of transport; iv) communication devices including mobile phones or radios, v) the internet; vi) land for gardening, vii) fishing equipment (please specify); viii) farming equipment (please specify); ix) livestock (please specify).

**Data Type**	Integer

1. Input access to asset types (Y/N), for livelihoods equipment, including the full list of available equipment types. (This process led to the identification of n=21 asset types important to support livelihoods in the local context; (1) generator; (2) fridge/freezer; (3) boat/canoe; (4) OBM; (5) other transport types; (6) communication devices; (7) internet; (8) land for gardening; (9) fishing line; (10) fishing hook; (11) fishing bolt; (12) diving gun; (13) diving spear; (14) net; (15) knife; (16) hoe; (17) axe; (18) bow; (19) spade; (20) pitchfork; (21) pig; (22) chicken).
2. Conduct Principal Component Analysis (PCA) using R package 'prcomp' to assign relative importance weightings to each asset type. Calculate rotation loadings for each PCA component and use % variance explanations (75.00%) to calculate the weighting of each livelihood-based asset. Sum asset types using the following equation where: *Resource Access* = $(\text{Resource}_1 * \text{Weight}_1) + (\text{Resource}_2 * \text{Weight}_2)$ etc., to obtain a final output for resource access. Normalise final indicator outputs onto a 0-1 scale to allow for comparability across the sample.
3. Categorise into indicator rankings where low access = very low adaptive capacity, and high access = very high adaptive capacity.

Indicator Rankings

**Adaptive Capacity Rank**		**Definition**	**Description**
1	Very Low	0-20% Access	Household has access to 0-20% of assets deemed important for livelihoods based on their relative importance.
2	Low	21-40% Access	Household has access to 21-40% of assets deemed important for livelihoods based on their relative importance.

3	Medium	41-60% Access	Household has access to 41-60% of assets deemed important for livelihoods based on their relative importance.
4	High	61-80% Access	Household has access to 61-80% of assets deemed important for livelihoods based on their relative importance.
5	Very High	81-100% Access	Household has access to 81-100% of assets deemed important for livelihoods based on their relative importance.

Key References

1. Aazami, M. & Shanazi, K. 2020. Tourism wetlands and rural sustainable livelihood: The case from Iran. *Journal of Outdoor Recreation and Tourism.* 30(1). Pp.100-113.
2. Becker, P. 2017. Dark side of development: Modernity, disaster risk and sustainable livelihoods in two coastal communities in Fiji. *Sustainability.* 9(12). Pp.215-231.
3. Blakie, P., Cannon, T., Davis, I., Wisner, B. *At Risk: Natural Hazards, People's Vulnerability and Disasters.* Routledge.
4. Brown, R.P., Tuan, V.V., Nhan, K.D., Dung, C.L. & Ward, J. 2018. Influence of livelihoods on climate change adaptation for smallholder farmers in the Mekong Delta Vietnam. *International Journal of Agricultural Sustainability.* 16(3). Pp.255-271.
5. Daramola, A.Y., Oni, O.T., Ogundele, O. & Adesanya, A. 2016. Adaptive capacity and coping response strategies to natural disasters: A study in Nigeria. *International Journal of Disaster Risk Reduction.* 15. Pp.132-147.
6. Fischer, C., Trono, B.R. & Salinas, F.A.R. 2021. Addressing gaps in socio-economic and natural assets to halt marine turtle egg poaching: A livelihood feasibility study in the Turtle Islands Wildlife Sanctuary, Philippines. *Global Ecology and Conservation.* 19. Pp.12-40.
7. Li, X., Xu, S. & Hu, Y. 2020. Understanding the rural livelihood stability system: The eco-migration in Huanjiang county, China. *Sustainability.* 12(6). Pp.634-657.
8. Rampengan, M.M., Law, L., Gaillard, J.C., Boedhihartono, A.K. & Sayer, J. 2016. Engaging communities in managing multiple hazards: Reflections from small islands in North Sulawesi, Indonesia. *Singapore Journal of Tropical Geography.* 37(2). Pp.249-267.
9. Thatsarani, U.S. & Gunaratne, L.H. 2018. Constructing and index to measure the adaptive capacity to climate change in Sri Lanka. *Procedia Engineering.* 1(212). Pp.278-285.

Natural Capital

N1: Fair Access to Natural Resources

Indicator Summary

Indicator: Household perception of fair access to natural resources within their community.

Indicator Description: The extent to which a household believes there is fair access to natural resources within their community, ranging from (1) strongly disagree; (2) disagree; (3) neither agree nor disagree; (4) agree and (5) strongly agree.

Measuring Unit: Perception of equitable access (5-point Likert Scale: Strongly Disagree – Strongly Agree).

Contribution to Adaptive Capacity

Summary: The more a household believes there is fair access to natural resources within their community, the greater their capacity to adapt to hazard exposure.

Justification: Fair access to resources has been deemed an important component of adaptive capacity, supporting a household's ability to build preparedness and develop adaptation strategies to overcome hazard exposure (Cafer et al. 2019). Fairness based indicators provide an understanding of marginalisation within a community, where access to natural resources may be limited (Williamson et al. 2012). Research has found perceptions of fairness are representative of resource access, with low perceptions (i.e., limited equitable access) reflective of corruption, marginalisation, and poor community relationships (Bennett et al. 2014). Unfair access to natural resources can reduce household adaptive capacity by i) limiting access to food, water, housing, energy or income; and ii) increasing dependence on degrading or depleting resource types (Jones et al. 2022; Susilo et al. 2021). Fair access to natural resources is essential in rural communities that heavily depend on the environment to access basic goods (Jones et al. 2022). Fair access to natural resources additionally enables households to access services that support adaptation, including land for gardening, access to food stocks, and protection from hazard exposure through natural buffers such as mangroves and coastal wetlands (DFID 1999).

Indicator Method

Indicator Origin & Adaptations

Origins: Natural capital indicators are not a common within CBVAs (Smith & Diedrich 2024). However, there is significant evidence to support their value for inclusion as a component of adaptive capacity (Cafer et al. 2019; Williamson et al. 2012). Where natural capital has been assessed studies commonly use perceptions of resource access, rather than a direct measurement of the availability of natural resource stocks (Chepkoech et al. 2020; Nawrotzki et al. 2012). This finding justified the use of a perception-based metric to quantify fair access to natural resources within the LRP vulnerability index.

Adaptations: n/a

Data Collection Instrument

Household Survey Question: How much do you agree with the following statement? Access to natural resources in my community is fair: (1) strongly disagree; (2) disagree; (3) neither disagree nor agree; (4) agree; (5) strongly agree.

Data Type: Ordinal

1. Input responses from household survey whereby (1) strongly disagree that access to new economic opportunities is fair = very low adaptive capacity, to (5) strongly agree that access to new economic opportunities is fair = very high adaptive capacity.

Indicator Rankings

Adaptive Capacity Rank		Definition	Description
1	Very Low	Strongly Disagree	Household strongly disagrees that resource access is fair.
2	Low	Disagree	Household disagrees that resource access is fair.
3	Medium	Neither Agree nor Disagree	Household neither agrees nor disagrees that resource access is fair.
4	High	Agree	Household agrees that resource access is fair.
5	Very High	Strongly Agree	Household strongly agrees that resource access is fair.

Key References

1. Bennett, J.N., Dearden, P., Murray, G. & Kadfak, A. 2014 The capacity to adapt? Communities in a changing climate, environment, and economy on the northern Andaman coast of Thailand. *Ecology & Society.* 19(2), Pp.1-21.
2. Cafer, A., Green, J. & Goreham, G. 2019. A community resilience framework for community development practitioners building equity and adaptive capacity. *Community Development.* 50, Pp.201-216.
3. Chepkoech, W., Mungai, W.N., Stober, S. & Lotze-Campen, H. 2020. Understanding adaptive capacity of smallholder African indigenous vegetable farmers to climate change in Kenya. *Climate Risk Management.* 27. Pp.100-123.
4. Jones, L.H.B., Unsworth, F.K.R., Nordlund, M.L., Elkof, S.J., Ambo-Rappe, R., Carly, F., Jiddawi, S.N., Nafie, A.Y., Udagedara, S. & Cullen-Unsworth. 2022. Dependence on seagrass fisheries governed by household income and adaptive capacity. *Ocean & Coastal Management.* 225, Pp.106-126.
5. Nawrotzki, J.R., Hunter, M.L. & Dickinson, W.T. 2014. Rural livelihoods and access to natural capital: Differences between migrants and non-migrants in Madagascar. *Demographic Research.* 26(10). Pp.123-129.
6. Susilo, E., Purwanti, P., Fattah, M., Qurrata, A.V. & Namaditya, S.B. 2021. Adaptive coping strategies towards seasonal change impacts: Indonesian small-scale fisherman household. *Heliyon.* 7, Pp.681-692.
7. Smith, B. and Diedrich, A., 2024. A systematic review of current progress in community based vulnerability assessments. *Regional Environmental Change*, *24*(1), pp.1-17.
8. Williamson, T., Hesseln. H. & Johnston, M. 2012. Adaptive capacity deficits and adaptive capacity of economic systems in climate change vulnerability assessment. *Forest Policy and Economics.* 1, Pp,160-166.

Water Insecurity

W1: Access to Drinking Water

Indicator Summary

Indicator: Household has access to improved sources of drinking water within a 30-minute return distance.

Indicator Description: Household has access to an improved source of drinking water as defined by the World Health Organisation (i.e., piped water, borehole, tube well, protected dug well, protected spring, packaged water, and rainwater) within a 30-minute return distance from the household. Whereby:

1. Very low sensitivity = drinking water is obtained from an improved source within the home.
2. Low sensitivity = drinking water is obtained from an improved source within 30 minutes from home.
3. Moderate sensitivity = drinking water is obtained from an improved source >30 minutes from home.
4. Drinking water is obtained from an unimproved water source (e.g., dug well or unprotected stream).
5. Drinking water is obtained from an unimproved water source (e.g., surface water comprising rivers, dams, lakes, ponds, steams, canals or irrigation channels).

Measuring Unit: i) Drinking water source and ii) distance of source from household.

Contribution to Sensitivity

Summary: Households with access to an improved source of drinking water close to home are considered to be less sensitive to water insecurity following hazard exposure.

Justification: Access to unimproved water sources is linked to the transmission of diseases such as cholera, diarrhoea, dysentery, hepatitis A, typhoid, and polio and has been shown to be a leading cause of morbidity and mortality, especially among children (Fowad et al 2021). Households with access to unimproved water sources are more likely to be impacted by these health conditions, reducing their overall livelihood security and their ability to respond to hazard exposure. Consumption of contaminated water also has economic implications associated to medical costs. In relation to distance from household, when water sources are located further from the home they take more time and physical effort to access, reducing time spent on productive activities, and limiting accessibility for less abled individuals (WHO 2022).

Indicator Method

Indicator Origin & Adaptations

Origins: This indicator was developed by the World Health Organisation and UNICEFs joint water monitoring programme (JMP) to support accurate information on drinking-water access. The indicator utilises household water source as a proxy for whether a household has access to improved (i.e., safe) drinking water (WHO 2006). The indicator requires information on the primary source of drinking water utilised by a household and separates this into i) improved and ii) unimproved sources. Where improved drinking water sources include piped water, boreholes, tube well, protected dug well, protected spring, packaged water and rainwater; and unimproved sources include unprotected dug wells, springs and surface water (WHO 2006). Water source is then paired with the total number of minutes it takes to obtain drinking water. The JMP transformed this information into a 5-point drinking water ladder, reflecting household access to safely managed water sources. This scale was directly utilised to generate indicator rankings.

Adaptations: n/a

Data Collection Instrument

Household Survey Question(s): What source(s) of water do you use for drinking in your household? Which of these sources is most important? Approximately how long does it take you to obtain drinking water from these sources (return time)?

**Data Type**	Text, Integer

1. Identify the most important drinking water source used by a household and categorise response into (1) improved sources of drinking water (e.g., piped water, boreholes, tube well, protected dug wells, protected springs, packaged water, rainwater), (2) unimproved water sources (e.g., unprotected dug well, spring) and (3) surface water (e.g., river, dam, lake, pond, stream, canal, irrigation channel) as designated by the World Health Organisation.
2. Categorise distance responses into (1) 0 minutes – in/around shelter; (2) <30 minutes; (3) <30 minutes.
3. Rank responses using the following ranking categories based on the JMP ladder classifications for household drinking water services.

Indicator Rankings			
**Sensitivity Rank**		**Definition**	**Description**
1	Very Low	Improved Access	Drinking water obtained from an improved drinking water source located within/next to a homestead.
2	Low	Basic Access	Drinking water obtained from an improved water source with a collection time of less than 30 minutes.
3	Medium	Limited Access	Drinking water obtained from an improved water source where collection time exceeds 30 minutes.
4	High	Unimproved Access	Drinking water obtained from an unprotected dug well or unprotected stream.
5	Very High	Surface Water	Drinking water collected from surface water (e.g., river, dam, lake, pond, stream, canal, irrigation channel).

Key References

1. Fowad, M., Mahvish, M., Tajammal, M. & Javaria, A. 2021. Water and sanitation risk exposure in children under-five in Pakistan. *Journal of Family and Community Medicine.* 28(2), pp. 103-109.
2. WHO. 2006. *Core Questions on Drinking-Water and Sanitation for Household Surveys.* World Health Organisation and UNICEF. Geneva, Switzerland. *Pp.1-24.*
3. WHO. 2022. *Guidelines for drinking-water quality: Fourth edition, incorporating the first and second addenda.* World Health Organisation.
4. JMP. 2023. Drinking Water. World Health Organisation. Available from: https://washdata.org/monitoring/ drinking water.

W2: Water Sufficiency

Indicator Summary

Indicator: Household has access to sufficient quantities of water to meet basic needs.

Indicator Description: The amount of time a household lacks access to the quantity of water required to meet basic needs ranging from 1) never; 2) rarely; 3) occasionally; 4) frequently; 5) all the time.

Measuring Unit: Amount of time (5-point Likert scale: 1) never to 5) all the time).

Contribution to Sensitivity

Summary: The more time a household lacks access to sufficient volumes of water, the more sensitive they are to water insecurity following hazard exposure.

Justification: Water sufficiency evaluates the ability of a household to obtain sufficient quantities of water to meet basic needs. The indicator has been shown to be particularly important in rural contexts, where water sufficiency is impacted by seasonal and topographical changes, and accessibility can be hampered based on transport availability (Calow et al. 2010; Dickson et al. 2016). In relation to sensitivity, households with limited water sufficiency will be more likely to be impacted by external shock and stressor events that influence water access (e.g., droughts) (Khastagir & Jayasuriya 2010). This is particularly prevalent in relation to climate change and natural resource extraction activities such as logging which can result in water source contamination associated to chemical use and sedimentation (Kumar et al. 2020; Minter & Van der Ploeg 2023).

Indicator Method

Indicator Origin & Adaptations

Origins: Drinking water sufficiency is utilised as an indicator within the SIIVA as a component of water security (SPC 2016). The LRP index attempts to closely align sensitivity indicators with the SIVA approach where households are asked if their ‘water supply meets household needs’, with Likert-scale response categories comprising 1) never; 2) rarely; 3) sometimes and 4) all the time.

Adaptations: Household survey responses were extended to cover a 5-point Likert scale ranging from 1) never to 5) all the time. This adaptation occurred to allow for comparability with other water-security based indicators that are represented on a 5-point scale, whilst also supporting further differentiation between instances of water insufficiency.

Data Collection Instrument

Household Survey Question(s): Over the past year, how often have you NOT been able to access a sufficient volume of water to meet household requirements, on a scale from (1) never to (5) all the time.

**Data Type**	Ordinal

1. Input responses from survey question whereby: low sensitivity (1) = household never lacks access to sufficient quantities of water, and high sensitivity (5) = household lacks access to sufficient quantities of water all the time.			
**Indicator Rankings**			
**Sensitivity Rank**		**Definition**	**Description**
1	Very Low	Never	Household never lacks access to sufficient volumes of water.
2	Low	Rarely	Household rarely lacks access to sufficient volumes of water.
3	Medium	Occasionally	Household occasionally lacks access to sufficient volumes of water.
4	High	Frequently	Household frequently lacks access to sufficient volumes of water.
5	Very High	All the Time	Household lacks access to sufficient volumes of water all the time.

Key References

1. Calow, R.C., MacDonald, A.M., Nicol, A.L. & Robins, N.S. 2010. Ground water security and drought in Africa: linking availability, access, and demand. *Ground Water.* 48(2), Pp.246-256.
2. Dickson, E.S., Schuster-Wallace, J.C. & Newton, J.J. 2016. Water security assessment indicators: The rural context. *Water Resources Management.* 30, Pp.1567-1604.
3. Khastagir, A. & Jayasuriya, N. 2010. Optimal sizing of rainwater tanks for domestic water conservation. *Journal of Hydrology.* 381, Pp.3-14.
4. Kumar, L., Jayasinghe, S., Gopalakrishnan, T. & Nunn, P.D. *Climate Change and the Pacific Islands.* In: Knowledge Management for Development.
5. Minter, T. & Van Der Ploeg. 2023. 'Our happy hour became a hungry hour': Logging, subsistence and social relations in Solomon Islands. *International Forestry Review.* 25(1), Pp.113-125.
6. SPC. 2016. *Integrated Vulnerability Assessment Framework for Atoll Islands. A collaborative approach.* Pacific Community, Secretariat of the Pacific Regional Environmental Programme and Deutsche Gellschaft fur Internationale Zusammenarbeit. Pp.1-74.

W3: Water Quality

Indicator Summary

Indicator: The extent to which a household is satisfied with the quality of their drinking water.

Indicator Description: The extent to which a household is satisfied with their drinking water quality ranging on a scale from 1) very satisfied; 2) somewhat satisfied; 3) neither satisfied nor unsatisfied; 4) somewhat unsatisfied and 5) very unsatisfied.

Measuring Unit: Household satisfaction (5-point Likert Scale: 1) very satisfied to 5) very unsatisfied).

Contribution to Sensitivity

Summary: Household's with higher levels of water quality dissatisfaction will be more sensitive to water insecurity following hazard exposure.

Justification: Poor water quality makes water unsafe for use by households, thereby reducing water availability and imposing multiple health implications and environmental impacts (Mishra et al. 2021). Water quality is heavily influenced by global change pressures including climatological factors such as drought, in addition to pollution via the intensification of human populations and activities (Lese et al. 2021). Results from several studies have shown that satisfaction with water quality can be linked to the presence of contaminants etc., within a water source (Delpa et al. 2020; Lee & Lee 2015).

Indicator Method

Indicator Origin & Adaptations

Origins: Drinking water quality is utilised as an indicator within the SIIVA as a component of water security (SPC 2016). The LRP index attempts to closely align sensitivity indicators with this approach given its application in Solomon Islands. Within the SIIVA water quality is measured by asking a household if they believe their drinking water supply is safe for consumption, with responses varying from 1) almost all of the time to 4) never.

Adaptations: Water quality was measured by extent of satisfaction rather than the amount of time a household believes their water supply is safe (SPC 2016). This adaptation was selected due to the ability for satisfaction to capture not only the objective aspects of water quality (i.e., chemical and biological composition), but also the subjective experience of the individuals consuming the water. This considers the needs and preferences of households and their willingness to consume different types of water sources (Denantes & Donoso 2021).

Data Collection Instrument

Household Survey Question: How happy are you with the quality of your drinking water? 1) very satisfied; 2) somewhat satisfied; 3) neither satisfied nor unsatisfied; 4) somewhat unsatisfied; 5) very unsatisfied.			
**Data Type**	Ordinal		
1. Input household survey responses whereby 1) very satisfied (very low sensitivity) and 5) very unsatisfied (very high sensitivity).			
**Indicator Rankings**			
**Sensitivity Rank**		**Definition**	**Description**
1	Very Low	Very Satisfied	Household is very satisfied with their drinking water quality.
2	Low	Somewhat Satisfied	Household is somewhat satisfied with their drinking water quality.
3	Medium	Neither Satisfied nor Unsatisfied	Household is neither satisfied nor unsatisfied with their drinking water quality.
4	High	Somewhat Unsatisfied	Household is somewhat unsatisfied with their drinking water quality.
5	Very High	Very Unsatisfied	Household is very unsatisfied with their drinking water quality.

Key References

1. Delpa, I., Legay, C., Proulx, F. & Rodriguez, J.M. 2020. Perception of tap water quality: Assessment of the factors modifying the links between satisfaction and water consumption behaviour. *Science of the Total Environment.* 722. Pp.136-145.
2. Denantes, J. & Donoso, G. 2021. Factors influencing customer satisfaction with water service quality in Chile. *Utilities Policy.* 73. Pp.101-123.
3. Lee, H.L. & Lee, D.Y. 2015. The impact of water quality on the visual and olfactory satisfaction of tourists. *Ocean and Coastal Management.* 105, Pp.92-99.
4. Mishra, K.B., Kumar, P., Saraswat, C., Chakraborty, S. & Gautman, A. 2021. Water security in a changing environment: Concept, Challenges and Solutions. *Water.* 2021. 13(4), pp. 490-499.
5. SPC. 2016. *Integrated Vulnerability Assessment Framework for Atoll Islands. A collaborative approach.* Pacific Community, Secretariat of the Pacific Regional Environmental Programme and Deutsche Gellschaft fur Internationale Zusammenarbeit. Pp.1-74.

W4: Access to Sanitation

Indicator Summary

Indicator: Household has access to an improved form of sanitation.

Indicator Description: Household has access to an improved form of sanitation (i.e., household does not practice open defecation), that is utilised by a single family (i.e., private).

Measuring Unit: i) Type of sanitation (e.g., most improved (slab toilet with flush), improved (slab toilet with pour) and unimproved (open defecation) and ii) number of households utilising sanitation type (e.g., private or shared).

Contribution to Adaptive Capacity

Summary: Household's that lack access to a private and improved form of sanitation will be more sensitive to water insecurity following hazard exposure.

Justification: Access to improved sanitation is a key contributor to health, wellbeing and water security. Unimproved sanitation practices (e.g., open defecation) have a range of impacts, including public health issues, child mortality and reductions in ecosystem quality (Giribabu et al. 2019). Open defecation has been implicated in high rates of infectious disease transmission and is a major driver of ill health in the areas where it is practiced (Freeman et al. 2017). Open defecation is of particular concern in areas that utilise groundwater and natural water sources for drinking, with consumption associated to high rates of waterborne diseases (Giribabu et al. 2019). To enhance water security, slab toilets have been introduced in rural areas, comprising slab pours (e.g., a toilet flushed with a bucket of water) or flush pours (e.g., a toilet with a mechanical flushing device). Of the two types, slab flush toilets are generally considered better in terms of healthcare. This is attributed to reduced contact with human waste and pathogens, and the effective removal of waste from mechanical flushing which further reduces the spread of waterborne diseases (Meili et al. 2021).

Indicator Method

Indicator Origin & Adaptations

Origins: Access to sanitation is an indicator that has been developed by the World Health Organisation, whereby households are asked the type of toilet facility that they utilise. The World Health organisation

classifies unimproved sanitation types as i) open defecation, and ii) slab toilets with pour, and improved sanitation as i) slab toilets with flush (WHO 2006). Additional sanitation practices are listed by the World Health Organisation however these were not relevant in the context of Solomon Islands where the LRP was applied.
**Adaptations:** This indicator differentiates slab toilets with pour as an 'improved' sanitation type, elevating slab toilet with flush to 'most improved'. This variation from the WHO indicator has taken place given significant differences in incidences of mortality and disease transmission between these type of sanitation practices (Giribabu et al. 2019). Differences in disease transmission have also been observed between households utilising shared versus private toilets, with shared toilets more likely to harbour pathogens (Meili et al. 2021). This has led to the development of shared versus private toilet type as an additional sanitation-based indicator within a number of studies (e.g., Evans et al. 2017; Meili et al. 2021), and was thus integrated as a component within the LRP indicator.
**Data Collection Instrument**
Household Survey Question: What is the main type of toilet facility used by your household? Do you share this facility with any other households?

**Data Type**	Ordinal

1. Input household survey data categorising type of toilet facility responses into 1) most improved (e.g., slab toilet with flush); 2) improved (e.g., slab toilet with pour) and 3) unimproved (e.g., open defecation).
2. Input household survey data categorising the number of households using a sanitation type into 1) shared or 2) private.
3. Combine type of toilet facility and number of households using a toilet facility and classify into the following ranking categories.

Indicator Rankings

**Sensitivity Rank**		**Definition**	**Description**
1	Very Low	Most improved	Household uses a highly improved toilet (e.g., slab toilet with flush).
2	Low	Improved (private)	Household uses a private and improved toilet type (e.g., slab toilet with pour).
3	Medium	Improved (shared)	Household uses a shared but improved toilet type (e.g., slab toilet with pour).
4	High	Unimproved (private)	Household uses an unimproved but private toilet type (e.g., open defecation).
5	Very High	Unimproved (shared)	Household uses an unimproved and shared toilet type (e.g., open defecation).

Key References

1. Evans, B., Hueso, A., Johnston, R., Norman, G., Perez, E., Slaymaker, T. & Tremolet, S. 2017. Limited services? The role of shared sanitation in the 2040 agenda for sustainable development. *Water, Sanitation & Hygiene for Development.* 7(3). Pp.349-351.
2. Freeman, C.M., Garn, V.J., Sclar, D.G., Boisson, S., Medlicott, K., Alexander, T.K., Penakalapati, G., Anderson, D., Mahtani, G.A., Grimes, E.T.J., Rehfuess, A.E. & Clasen, F.T. 2017. The impact of sanitation on infectious disease and nutritional status: A systematic review and meta-analysis. *International Journal of Hygiene and Environmental Health.* 220(6), Pp.928-949.
3. Giribabu, D., Bharadwaj, P., Sitiraju, R., Burra, M., Rao, P.P. & Reddy, S.C. 2019. Combating open defecation through community-led sanitation. *Al Habib Medical Journal.* 1(3), Pp.45-51.
4. Meili, D., Schelbert, V., Alam, M., Antwi-Agyei, P., Simiyu, S., Amaning, K., Dwumfour-Asare, B., Rahman, M., Luthi, C. & Gunther, I. 2021. Indicators for sanitation quality in low-income urban settlements: Evidence from Kenya, Ghana, and Bangladesh. *Social Indicators Research.* 162. Pp.683-720.
5. WHO. 2006. *Core Questions on Drinking-Water and Sanitation for Household Surveys.* World Health Organisation and UNICEF. Geneva, Switzerland. Pp.1-24.

Food Insecurity

F1: Food Sufficiency

Indicator Summary

Indicator: Household has uninterrupted access to sufficient volumes of food to meet household requirements.

Indicator Description: Household has access to food at all times at a rate that meets household requirements.

Measuring Unit: Extent of food sufficiency (5-point Likert scale: 1 – never lack access to sufficient volumes of food to meet household requirements; to 5 – always lack access to sufficient volumes of food to meet household requirements).

Contribution to Sensitivity

Summary: A household with uninterrupted access to sufficient volumes of food to meet requirements will be less sensitive to food insecurity following hazard exposure.

Justification: Food sufficiency refers to instances where a household has enough food to meet their basic dietary needs (Coates et al. 2007). If a household has enough food, it is considered food sufficient. Food sufficiency is a fundamental component of food security. When a household cannot access enough food, they may begin to suffer from undernutrition, malnutrition and micronutrient deficiencies (Siddiqui et al. 2020). In time, this lack of food can lead to long term health issues including stunted growth, impaired cognitive development and increased risk of disease (Siefert et al. 2004). Food insufficiency can also drive economic and social impacts within a household. This is commonly observed when households attempt to combat food insufficiency by lending or selling assets, increasing a household's risk of poverty (Siddiqui et al. 2020). Food insufficiency can be further compounded by hazard exposure (Wheeler & Von Braun 2013). This is of particular relevance in rural environments that depend on self-production (i.e., gardening and fishing) for their food intake (Schwarz et al. 2013). Significant evidence has documented the negative impacts of natural hazards (such as climate change), and human activities (such as land use change), on the productivity of home-grown and caught food products, with flow on implications for food sufficiency (Thanichanon et al. 2018; Wheeler & Von Braun 2013).

Indicator Method

Indicator Origin & Adaptations

Origins: The Household Food Insecurity Access Scale (HFIAS) developed by Coates et al. (2007) is a widely applied indictor representing food sufficiency. The indicator works by addressing 9 food occurrence related questions measuring access to sufficient volumes of food to meet household requirements. Households are asked to reflect on instances where they lacked sufficient food access in the previous four weeks, with responses recorded on a 4-point Likert scale comprising lack of access occurring 1) never; 2) rare, 3) sometimes and 4) often.

Adaptations: The HFIAS indicator was not applied in its entirety in the LRP. Instead, the most essential question related to food sufficiency was extracted from the HFIAS (i.e., 'how often did your household does not have enough food') and applied as a standalone metric of food sufficiency. This basic measure has been utilised within a number of previous vulnerability assessments (e.g., Busse 2017; Pham et al. 2020), and has been evidenced to provide a quick, accurate, direct and culturally sensitive metric of food sufficiency (Webb et al. 2022). This adaptation was made given the extensive nature of the LRP household survey, with a streamlined food sufficiency indicator helping prevent participant fatigue. Likert-scale responses were additionally extended to a 5-Point scale given the use of this range across the majority of indicators within the LRP index.

Data Collection Instrument

Household Survey Question(s): In the past four weeks, how many times has the following question statement applied: your household did not have enough food? Respond on a scale from (1) never; (2) rarely; (3) sometimes; (4) often; (5) always

Data Type: Ordinal

1. Input household survey responses into ranking categories whereby (1) very low sensitivity = household never lacked access to sufficient volumes of food, and (5) very high sensitivity = household lacked access to sufficient volumes of food all the time.

Indicator Rankings

Sensitivity Rank		Definition	Description
1	Very Low	Never	Household never lacked access to sufficient volumes of food.
2	Low	Rarely	Household rarely lacked access to sufficient volumes of food.
3	Medium	Sometimes	Household sometimes lacked access to sufficient volumes of food.
4	High	Often	Household often lacked access to sufficient volumes of food.
5	Very High	All the time	Household lacked access to sufficient volumes of food all the time.

Key References

1. Busse, A.H., Jogo, W., Fofanah, M., Tesfay, H., Hadush, M., Kiflom, E. & Schulz, S. 2017. Participatory assessment of factors influencing nutrition and livelihoods in rural Ethiopia: Implications for measuring impacts of multisector nutrition programmes. *Food and Nutrition Bulletin.* 38(4). Pp.468-484.
2. Coates, J., Swindale, A. & Bilinksy, P. 2007. *Household Food Insecurity Access Scale (HFIAS) for Measurement of Food Access: Indicator Guide (v3).* Food and Nutrition Technical Assistance Project, Academy for Educational Development. Washington, US. Pp.1-36.
3. Pham, T.T.N., Nong, D., Sathyan, R.A. & Garschagen, M. 2020. Vulnerability assessment of households to flash floods and landslides in the poor upland regions in Vietnam. *Climate Risk Management.* 28. Pp.121-135.
4. Schwarz, M.A., Bene, C., Bennett, G., Boso, D., Hilly, Z., Paul, C., Posala, R., Sibiti, S. & Andrew, N. 2011. Vulnerability and resilience of remote rural communities to shocks and global changes: Empirical analysis from Solomon Islands. *Global Environmental Change.* 21(3), Pp.1128-1140.
5. Siddiqui, F., Salam, A.R., Lassi, Z.S. & Das, K.J. 2020. The intertwined relationship between malnutrition and poverty. *Frontiers in Public Health.* 8. Pp.453-464.
6. Siefert, K., Heflin, M.C. & Williams, R.D. 2004. Food insufficiency and physical and mental health in a longitudinal survey of welfare recipients. *Journal of Health and Social Behaviour.* 45(2). Pp.204-218.
7. Thanichanon, P., Schmidt-Vogt, D., Epprecht, M., Henimann, A. & Wiesmann, U. 2018. Balancing cash and food: The impacts of agrarian change on rural land use and wellbeing in Northern Laos. *PLOS One.* 13(12). Pp.166-189.
8. Webb, P., Coates, J. & Houser, R. 2002. Allocative responses to scarcity: Self-reported assessments of hunger compared with conventional measures of poverty and malnutrition in Bangladesh. *Food Policy and Applied Nutrition.* 20(13), Pp.1-41.
9. Wheeler, T. & Von Braun, J. 2013. Climate change impacts on global food security. *Science.* 341(6415). Pp.508-513.

F2: Food Diversity

Indicator Summary

Indicator: The frequency of consumption of different food groups during the previous 7 days.

Indicator Description: Proxy measurement for dietary diversity representing the number of days a household has consumed different types of food groups in the previous week (i.e., 7 days), multiplied by a food groups nutritional value. Food groups include i) main staples (e.g., rice, flour, roots and tubers), ii) pulses (e.g., grains and nuts); iii) vegetables; iv) fruits; v) meat; vi) fish; vii) dairy; viii) sugar and ix) oil.

Measuring Unit: Number of days a household has consumed different food groups.

Contribution to Sensitivity

Summary: The larger a household's food consumption score (i.e., the greater the amount of nutritionally rich food groups a household consumed in the previous 7-day period) the lower a household's sensitivity to food insecurity following hazard exposure.

Justification: Consuming a range of diverse food types that are rich in nutrients (e.g., minerals, proteins and vitamins) is a vital component of food security (World Food Programme 2015). A household that consumes a range of nutritionally rich food sources on a daily basis will be less at risk of malnutrition and associated health related issues. In contrast, a household that relies on a narrow range of nutritionally limited food sources may develop nutritional deficiencies (Verger et al. 2021). Households with limited dietary diversity may also be more at risk of impact from hazard exposure where hazards restrict access to nutritionally diverse food types. In these instances, household food security will be heavily compromised if alternate food sources are not available (Niles et al. 2021).

Indicator Method

Indicator Origin & Adaptations

Origins: The Food Consumption Score (FCS) was developed by the World Food Programme (2015). The FCS measures household dietary diversity by determining a 7-day day recall of the types of food groups a household has consumed and the frequency of consumption, which is then compared against each food groups nutritional value (Sileshi et al. 2012). The FCS indicator was selected to represent dietary diversity in the LRP over the Dietary Diversity Index Score developed by the Fanta II Project (Kennedy et al. 2011). This decision reflects the improved ability of the FCS to incorporate the nutritional value of food types as a component of dietary diversity. In this way, the FCS reflects not only the variety of food types a household has consumed, but also overall energy intake and nutritional value (Kennedy et al. 2010). The data collection and indicator calculation method for the FCS has been outlined below.

Adaptations: Indicator Rankings were developed based on the range of FCS scores across all case study communities, allowing for the development of a 5-point indicator scale.

Data Collection Instrument

Household Survey Question: How many times has your household eaten these different types of food in the past week (7 days)? i) main staples (e.g., rice, flour, roots and tubers), ii) pulses (e.g., grains and nuts); iii) vegetables; iv) fruits; v) meat; vi) fish; vii) dairy; viii) sugar and ix) oil.	
**Data Type**	Integer

1. List the number of days each food group was consumed by a household.
2. Multiply the number of days each food group was consumed by a household based on the nutritional value designated by WFP (2015). i.e., days consumed x nutritional weight = weighted value.

Food Group	1) Days Consumed	Nutritional Weight	2) Weighted Value
Main Staples	6	2	12
Pulses	1	3	3
Vegetables	6	1	6
Fruit	3	1	3
Meat & Fish	4	4	16
Dairy	1	4	4
Sugar	1	0.5	0.5
Oil	1	0.5	0.5

3. Sum the values of each food group to obtain a food consumption score.

$$FCS = 12 + 3 + 6 + 3 + 16 + 4 + 0.5 + 0.5$$

4. Use the indicator rankings listed below to designate a sensitivity rank for food consumption scores based on the total range of FCS across partner communities. Whereby very low sensitivity (1) = very high food consumption score (i.e., household has consumed a large amount of nutritional foods over the previous 7 days), and very high sensitivity (5) = very low food consumption score (i.e., household has consumed a small amount of nutritional foods over the previous 7 days).

Indicator Rankings

Sensitivity Rank		Definition	Description
1	Very Low	89.7 – 112.00	Household has a very high food consumption score.
2	Low	67.3 – 89.6	Household has a high food consumption score.
3	Medium	44.9 – 67.2	Household has a moderate food consumption score.
4	High	22.5 – 44.8	Household has a low food consumption score.
5	Very High	0.00 - 22.4	Household has a very low food consumption score.

Key References

1. Kennedy, G., Berardo, A., Papavero, C., Horjus, P., Ballard, T., Dop, M.C., Delbaere, J. & Brouwer, D.I. 2010. Proxy measures of household for food security assessment and surveillance: comparison of the household dietary diversity and food consumption scores. *Public Health Nutrition.* 13(12), Pp.2010-2018
2. Kennedy, G., Ballard, T. & Dop, M.C. 2011. *Guidelines for Measuring Household and Individual Dietary Diversity.* Fanta II Project, Food and Agricultural Organisation. Rome, Italy. Pp.1-60.
3. Niles, T.M., Emery, F.B., Wiltshire, S., Brown, E.M., Fisher, B. & Ricketts, H.T. 2021. Climate impacts associated with reduced dietary diversity in children across nineteen countries. *Environmental Research Letters.* 16. Pp.150-129.
4. Sileshi, M., Sieber, S., Lejissa, T. & Ndyetabula, W.D. 2022. Drivers of rural households' food insecurity in Ethiopia: A comprehensive approach of calorie intake and food consumption score. *Agricultural Economics Research, Policy and Practice in Southern Africa.* Pp.1-23.
5. Verger, E.O., Le Port, A., Borderon, A., Bourbon, G., Moursi, M., Savy, M., Mariotti, F. & Martin-Prevel, Y. 2021. Dietary diversity indicators and their associations with dietary adequacy and health outcomes: a systematic scoping review. *Advances in Nutrition.* 12(5). Pp.1659-1672.
6. World Food Programme. 2015. *Food Consumption Score Index.* Available at: https://www.wfp.org/publications/meta-data-food-consumption-score-fcs-indicator

Housing Insecurity

H1: Housing Condition

**Indicator Summary**
**Indicator:** The type of material used for the roof, flooring and walls of a household's shelter.

Indicator Description: The walls, floor and roof of a household's shelter are comprised of strong materials (e.g., iron, timber and concrete) that will be capable of withstanding shock and stressor events.

Measuring Unit: Type of material (i.e., solid or weak).

Contribution to Sensitivity

Summary: Household's with solid shelters (i.e., the walls, floor and roof are made of strong housing materials) will be less sensitive to housing insecurity following hazard exposure.

Justification: Poor housing condition (i.e., structures comprised of weak materials) increases the sensitivity of a household to impact from exposure to physical shock and stressor events such as flooding, cyclones, and storm surges (Ajibade & McBean 2014). Households with shelters comprised of weak materials are also more likely to be impacted by seasonal weather events such as monsoonal rainfall (Haddad et al. 2022). Poor housing condition can create a cycle of additional physical and psychological challenges, with negative impacts on health and wellbeing (Bonnefoy 2007). Costs of maintenance and repair can also increase a household's financial sensitivity and reduce adaptive capacity by forcing a household to spend their time and resources on improving housing condition (Barnett 2010).

Indicator Method

Indicator Origin & Adaptations

Origins: Housing condition is addressed in many CBVAs based on the types of material used for the roofs, walls, and flooring of a shelter (Smith & Diedrich 2024). Housing condition has also been utilised as an SDG indicator, with the structural quality and durability of housing materials a key component of 'adequate or inadequate' housing (UN 2021). In this indicator, strong housing materials comprise high strength, durability and resistance to environmental factors (e.g., iron, timber and concrete), contrasting to weak housing materials (e.g., bush materials such as Sago palm) which are more likely to degrade over time (UN 2021).

Adaptations: This indicator combined the strength of roof, wall, and flooring materials into a single measure of housing condition. Equal importance was given to each component given the UN requirement for component to be solid and in good condition (UN 2021).

Data Collection Instrument

Household Survey Question: What material is the i) roof; ii) floor; iii) walls of your shelter made from?

**Data Type**	Text

1. Input household survey responses documenting the type of material used for a household's i) roof; ii) floor and iii) walls.
2. Classify each type of material as strong (i.e., iron, timber and concrete) or weak (i.e., sago palm).
3. Sum the total number of strong housing materials within a shelter.
4. Classify housing condition using the following rankings where very low sensitivity = strong housing condition (e.g., the floor, roof and walls of a shelter are made from strong materials), and high sensitivity = weak housing condition (e.g., the floor, roof and walls of a shelter are made from weak materials).

Indicator Rankings

**Sensitivity Rank**		**Definition**	**Description**
1	Very Low	Excellent Housing Condition	The roof, floor, and walls of a shelter are comprised of strong housing materials.
2	Low	Good Housing Condition	Two components of a shelter (e.g., roof and floor) are comprised of strong housing materials.
3	High	Fair Housing Condition	One component of a shelter (e.g., roof) is comprised of strong housing materials.
4	Very High	Poor Housing Condition	No components of a shelter are comprised of strong housing materials.

Key References

1. Ajibade, I. & McBean, G. 2014. Climate extremes and housing rights: A political ecology of impacts, early warning and adaptation constraints in Lagos slum communities. *Geoforum.* 55, Pp.76-86.
2. Barnett, J. 2010. Human rights and vulnerability to climate change. In: Humphreys, S. (Ed.), Human Rights and Climate Change. University of Cambridge, UK. Pp. 257-271.
3. Bonnefoy, C. 2007. Inadequate housing and health: An overview. *International Journal of Environment and Pollution.* 30(3). Pp.411-429.
4. Haddad, S., Paolini, R., Synnefa, A., Torres, D.L., Prasad, D. & Santamouris, M. 2022. Integrated assessment of the extreme climatic conditions, thermal performance, vulnerability, and well-being in low-income housing in the subtropical climate of Australia. *Energy and Building.* 272, Pp.112-124.

5. United Nations. 2021. SDG Indicator Metadata: Proportion of urban population living in slums, informal settlements, or inadequate housing. Available from: www.https://unstats.un.org/sdgs/metadata/files /Metadata-11-01-01.pdf.
6. Smith, B. and Diedrich, A., 2024. A systematic review of current progress in community based vulnerability assessments. *Regional Environmental Change*, *24*(1), pp.1-17.

Energy Insecurity

E1: Cooking Fuel Sufficiency

Indicator Summary

Indicator: The amount of time a household has problems accessing cooking fuel.

Indicator Description: The amount of time a household reports that they have problems accessing cooking fuel. This is a self-reported measure of cooking fuel access based on a 5-point Likert Scale reflecting the amount of time a household lacks access, ranging from 1) never to 5) all the time.

Measuring Unit: Access to cooking fuel (5-point Likert scale: (1) never; (5) all the time).

Contribution to Sensitivity

Summary: The greater the amount of time a household struggles to access cooking fuel, the more sensitive they are to energy insecurity following hazard exposure.

Justification: Sufficient access to cooking fuel (in particular from modern fuel sources such as electricity) has been shown to drastically improve well-being and quality of life (Gonzalez-Eguino 2015). While lack of cooking fuel is associated to negative socio-economic impacts, particularly in low-income rural areas (Kanagawa & Nakata 2008). These impacts mainly relate to reductions in time availability and increased energy expenditure for household members that acquire fuel (e.g., firewood) (Guta 2014; Schat & Gilbert 2014). Additional negative health impacts have also been observed for households that use open fires, including respiratory and cardiovascular problems (Prunedo-Alvarez et al. 2012). Households that depend on natural resources for cooking (i.e., firewood, coconut husks etc.,), may also experience greater impact following hazard exposure (Rajesh et al. 2014). This is of particular prevalence in relation to human activities such as logging and mining that result in deforestation (Minter & Van der Ploeg 2023).

Indicator Method

Indicator Origin & Adaptations

Origins: The International Energy Agency classify 'household access to a minimum level of energy' as a key indicator of energy security (IEA 2007). Household access to energy has been utilised in previous CBVAs as an indicator of sensitivity, where the utilisation of self-reporting methods are common (Begg 2021). Self-reporting has been validated as an accurate measure of energy access by Warriner et al. (1984).

Adaptations: This indicator utilised a 5-Point Likert scale to reflect the amount of time a household lacks access to cooking fuel where (1) never; (2) rarely; (3) frequently; (4) sometimes; (5) all the time.

Data Collection Instrument

Household Survey Question: In the past year, were there times when you didn't have any access to cooking fuel? If yes, how many times did this take place? I) never; ii) rarely; iii) occasionally; iv) frequently; v) all the time.

Data Type: Ordinal

1. Input household survey data reflecting household access to cooking fuel whereby i) household never has problems accessing cooking fuel represents (1) low sensitivity, and v) household always has problems accessing cooking fuel represents (5) high sensitivity.

Indicator Rankings

Sensitivity Rank		Definition	Description
1	Very Low	Never	Household never has problems accessing cooking fuel.
2	Low	Rarely	Household rarely has problems accessing cooking fuel.
3	Medium	Occasionally	Household occasionally has problems accessing cooking fuel.
4	High	Frequently	Household frequently has problems accessing cooking fuel.
5	Very High	All the Time	Household has problems accessing cooking fuel all the time.

Key References

1. Begg, S.S., Ramon, A., Yeurt, N. & Iese, V. 2021. Integrated flood vulnerability assessment of villages in the Waimanu river catchment in the South Pacific: the case of Viti Levu, Fiji. *Regional Environmental Change.* 21(3). Pp.83-92.

2. Gonzalez-Eguino, M. 2015. Energy poverty: An overview. *Renewable and Sustainable Energy Reviews.* 47, Pp.377-385.
3. Guta, D.D. 2014. Effect of fuelwood scarcity and socio-economic factors on household bio-based energy use and energy substitution in rural Ethiopia. *Energy Policy.* 75, Pp.217-227.
4. IEA. 2020. *Defining energy access: 2020 methodology.* International Energy Agency. Available at: https://www.iea.org/articles/defining-energy-access-2020-methodology.
5. Kanagawa, M. & Nakata, T. 2008. Assessment of access to electricity and the socio-economic impacts in rural areas of developing countries. *Energy Policy.* 36(6), Pp.2016-2029.
6. Minter, T. & Van der Ploeg, J. Our happy hour became a hungry hour: logging, subsistence and social relations in Solomon Islands. *International Forestry Review.* 25. Pp.113-135.
7. Pruneda-Alvarez, L.G., Perez-Vazquez, F.J., Salgado-Bustamante, M., Pelallo-Martinez, A.N. & Perez-Maldonado, I.N. 2012. Exposure to indoor air pollutants (polycyclic aromatic hydrocarbons, toluene, benzene) in Mexican indigenous women. *Indoor Air.* 22, Pp.140-147.
8. Rajesh, S., Jain, S., Sharma, P. & Bhahuguna, R. 2014. Assessment of inherent vulnerability of rural communities to environmental hazards in Kimsar region of Uttarakhand India. *Environmental Development.* 12. Pp.16-36.
9. Schatz, E. & Gilbert, L. 2014. "My legs affect me a lot….I can no longer walk to the forest to fetch firewood": Challenges related to health and the performance of daily tasks for older women in a high HIV context. *Health Care for Women International.* 35, Pp.771-788.
10. Warriner, K.G., McDougall, H.G.G. & Claxton, D.J. 1984. Any data or none at all? Living with inaccuracies in self-reports of residential energy consumption. *Environment and Behaviour.* 16(4). Pp.12-23.

E2: Lighting Sufficiency

Indicator Summary

Indicator: The amount of time a household has problems accessing lighting.

Indicator Description: The amount of time a household reports that they have problems accessing lighting. This is a self-reported measure of lighting access based on a 5-point Likert Scale reflecting the amount of time a household lacks access, ranging from 1) never to 5) all the time.

Measuring Unit: Access to lighting (5-point Likert scale: (1) never; (5) all the time).

Contribution to Sensitivity

Summary: The greater the amount of time a household struggles to access lighting, the more sensitive they are to energy insecurity following hazard exposure.

Justification: Sufficient access to lighting (in particular from modern fuel sources such as electricity) has been shown to drastically improve well-being and quality of life (Gonzalez-Eguino 2015). This is attributed to the role lighting plays in enabling daily activities to be conducted, such as reading, cooking, studying and socialising (Sharma et al. 2019). Lighting also increases the ability for households to conduct livelihood-based activities during night-time (Kanagawa & Nakata 2008). Lighting has also been shown to increase safety within a community, reducing the risk of accidents and falls in unlit conditions, and increasing an individual's sense of security when moving around (Shahsavari & Akbari 2018). Households that have limited access to lighting and depend on natural resources (i.e., firewood) as their energy source, may additionally experience greater risk of energy insecurity following hazard exposure where these resources have been depleted, or can't be readily accessed (Rajesh et al. 2014).

Indicator Method

Indicator Origin & Adaptations

Origins: The International Energy Agency classify 'household access to a minimum level of energy' as a key indicator of energy security (IEA 2007). Household access to energy has been utilised in previous CBVAs as an indicator of sensitivity, where the utilisation of self-reporting methods are common (Begg 2021). Self-reporting has been validated as an accurate measure of energy access by Warriner et al. (1984).

Adaptations: This indicator utilised a 5-Point Likert scale to reflect the amount of time a household lacks access to lighting where (1) never; (2) rarely; (3) frequently; (4) sometimes; (5) all the time.

Data Collection Instrument

Household Survey Question: In the past year, were there times when you didn't have any access to lighting? If yes, how many times did this take place? I) never; ii) rarely; iii) occasionally; iv) frequently; v) all the time.

**Data Type**	Ordinal

1. Input household survey data reflecting household access to lighting whereby i) household never has problems accessing lighting represents (1) low sensitivity, and v) household always has problems accessing cooking fuel represents (5) high sensitivity.

Indicator Rankings			
**Sensitivity Rank**		**Definition**	**Description**
1	Very Low	Never	Household never has problems accessing lighting.
2	Low	Rarely	Household rarely has problems accessing lighting.
3	Medium	Occasionally	Household occasionally has problems accessing lighting.
4	High	Frequently	Household frequently has problems accessing lighting.
5	Very High	All the Time	Household has problems accessing lighting all the time.

Key References

1. Begg, S.S., Ramon, A., Yeurt, N. & Iese, V. 2021. Integrated flood vulnerability assessment of villages in the Waimanu river catchment in the South Pacific: the case of Viti Levu, Fiji. *Regional Environmental Change.* 21(3). Pp.83-92.
2. Gonzalez-Eguino, M. 2015. Energy poverty: An overview. *Renewable and Sustainable Energy Reviews.* 47, Pp.377-385.
3. IEA. 2020. *Defining energy access: 2020 methodology.* International Energy Agency. Available at: https://www.iea.org/articles/defining-energy-access-2020-methodology.
4. Kanagawa, M. & Nakata, T. 2008. Assessment of access to electricity and the socio-economic impacts in rural areas of developing countries. *Energy Policy.* 36(6), Pp.2016-2029.
5. Rajesh, S., Jain, S., Sharma, P. & Bhahuguna, R. 2014. Assessment of inherent vulnerability of rural communities to environmental hazards in Kimsar region of Uttarakhand India. *Environmental Development.* 12. Pp.16-36.
6. Shahsavari, A. & Akbari, M. 2018. Potential of solar energy in developing countries for reducing energy-related emissions. *Renewable and Sustainable Energy.* 90, Pp.275-291.
7. Sharma, R., Choudhary, D., Kumar, P., Venkateswaran, J. & Solanki, S.C. 2019. Do solar study lamps help children study at night? Evidence from rural India. *Energy for Sustainable Development.* 50, Pp.109-116.
8. Warriner, K.G., McDougall, H.G.G. & Claxton, D.J. 1984. Any data or none at all? Living with inaccuracies in self-reports of residential energy consumption. *Environment and Behaviour.* 16(4). Pp.12-23.

Income Insecurity

I1: Income Stability

Indicator Summary

Indicator: The extent to which household income has fluctuated over the previous 12 months.

Indicator Description: Household measure of changes in overall income rates from one year to the next reflecting an increase, decrease, or no change.

Measuring Unit: Perception of change in household income.

Contribution to Sensitivity

Summary: Household's with stable or increasing income will be less sensitive to income insecurity following hazard exposure than household's with declining income.

Justification: Income security is acknowledged as a key aspect of financial security, with financial instability reducing a household's ability to meet daily life expenses and reducing their capacity to respond to hazard exposure (Lee & Sabri 2017). Abubakar et al. (2018) and Mian et al. (2017) have empirically demonstrated this relationship, with a direct correlation between financial stability and household debt. As such, household's demonstrating less financial instability in relation to household income are more likely to exhibit long-term financial security (Lee & Sabri 2017).

Indicator Method

Indicator Origin & Adaptations

Origins: Perception of change in household income from one year compared to the previous year has been used as a metric of financial insecurity in a range of vulnerability assessments (e.g., Hoffmann & McNair 2019; Lee & Sabri 2017; Noerhidajatai et al. 2021;), including the IVA approach (SPC 2016). Likert-scales are a common method used to assess income changes from one year to the next.

Adaptations: n/a

Data Collection Instrument

Household Survey Question: Was your household's income this year higher, lower, or about the same as last year?

**Data Type**	Ordinal

1. Input household survey data reflecting income stability where households income i) remained the same; ii) was higher; iii) was lower than the previous year, where higher income = low sensitivity and lower income = high sensitivity.			
**Indicator Rankings**			
**Sensitivity Rank**		**Definition**	**Description**
1	Low	Higher income	Household income increased from the previous 12 month period.
2	Medium	Stable income	Household income remained stable from the previous 12 month period.
3	High	Lower income	Household income reduced from the previous 12 month period.
**Key References**			

1. Abubakar, A., Astuti, R. & Oktapiani, R. 2018. The analysis of risk profile and financial vulnerability of households in Indonesia. *Bulleting Economic.* 20(4). Pp.443-474.
2. Hoffmann, I.O.A. & McNair, J.S. 2019. How does consumers' financial vulnerability relate to positive and negative financial outcomes? The mediating role of individual psychological characteristics. *The Journal of Consumer Affairs.* 53(4). Pp.1630-1673.
3. Lee, P.M. & Sabri, F.M. 2017. Review of financial vulnerability studies. *Archies of Business Research.* 5(2). Pp.127-136.
4. Mian, A., Sufi, A., & Verner, E. 2017. How does credit supply expansion affect the real economy? The productive capacity and household demand channels. *American Economic Review.* 101. Pp.9-15.
5. Noerhidajatai, S., Purwoko, B.A., Werdaningtyas, H., Kamil, I.A. & Dartanto, T. 2021. Household financial vulnerability in Indonesia: Measurement and determinants. *Economic Modelling.* 96. Pp.433-444.
6. SPC. 2016. *Integrated Vulnerability Assessment Framework for Atoll Islands. A collaborative approach.* Pacific Community, Secretariat of the Pacific Regional Environmental Programme and Deutsche Gellschaft fur Internationale Zusammenarbeit. Pp.1-74.

Supplementary Information 2: Household Survey

**Survey Number**	
**Interviewer Name**	
**Date of Interview**	
**Time of Interview**	

**Have you marked the location of the household on the community map?**
Yes ☐
No ☐
If Not, why?

**Q1**	Sex of participant (Household head)
Male ☐	
Female ☐	

**Q2**	How old are you?

**Q3**	What are your main livelihood activities?
1	
2	
3	
4	
5	

**Q4**	Are you originally from this community?
Yes ☐ (**If YES,** go to Q8)	
No ☐	
**Q5**	**If NO,** where are you originally from?
**Q6**	**IF NO,** how did you come to live in this community?
**Q7**	**If NO,** how long have you lived within this community?

Q8	How many children live in your household?

Q9	Q10	Q11		Q12
What other adults live in your household?	How old is each household member?	What sex is each household member?		What is the **main** livelihood activity conducted by each household member?
		Male	Female	
		☐	☐	
		☐	☐	
		☐	☐	
		☐	☐	
		☐	☐	
		☐	☐	
		☐	☐	
		☐	☐	
		☐	☐	
		☐	☐	

Q13	What is the most important livelihood activity within your household?
**Q14**	What is second most important?
**Q15**	What is third most important?

Q16	How much of the food that your household catches & grows did you sell in the last year?			
Almost None	Some	About Half	Most	Almost All
1	2	3	4	5

Q17	How important is **access** to the following resources within your household?				
	Unimportant	Somewhat Unimportant	Neither Important nor Unimportant	Somewhat Important	Important
Drinking Water	1	2	3	4	5
Food	1	2	3	4	5
Lighting	1	2	3	4	5
Fuel for Cooking	1	2	3	4	5
Building Materials	1	2	3	4	5
Healthcare	1	2	3	4	5
Income	1	2	3	4	5

Q18	Q19	Q20
What sources of water do you use for drinking?	How long does it take you to get to this drinking water?	How do you get there?

**Q21**	Which water source is most important for your household?
**Q22**	Which is the second most important?
**Q23**	Which is the third most important?

**Q24**	Does it ever cost you money to access water?			
Never	Rarely	Sometimes	Frequently	All the Time
1	2	3	4	5
**Q25**	How often does your household pay for bottled water?			
Never	Rarely	Sometimes	Frequently	All the Time
1	2	3	4	5
**Q26**	**(If 2-5)** Approximately how much do you spend getting water in a month?			
SI$:				

**Q27**	Does the amount of water that you get meet the needs of your household?		
Yes ☐ **(If YES**, go to Q29)			
No ☐			
**Q28**	**IF NO, <u>how often</u>** does your household not have enough water?		
Rarely	Occasionally	Frequently	All the Time
☐	☐	☐	☐

**Q29**	How happy are you with the quality of your drinking water?			
Very Unsatisfied	Somewhat Unsatisfied	Neither Satisfied or Unsatisfied	Somewhat Satisfied	Very Satisfied
1	2	3	4	5
**Q30**	**(IF 1-4)** What is affecting the quality of your drinking water?			

Q31	In the past year has anything affected your ability to access water?
Yes ☐	
No ☐ (**If NO**, go to Q34)	

Q32		Q33				
**If YES,** what is the name of the problem?		**If YES,** how often have you experienced these problems?				
		Very Rarely	Rarely	Occasionally	Frequently	All the Time
A		1	2	3	4	5
B		1	2	3	4	5
C		1	2	3	4	5
D		1	2	3	4	5
E		1	2	3	4	5

Q34	What is the main type of toilet facility used by your household (e.g., mangroves, bush, slab toilet with pour, slab toilet with flush)?

Q35	Do you share this toilet facility with any other households?
Yes	
No	

Q36	How happy are you with sanitation within your household/community?			
Very Unsatisfied	Somewhat Unsatisfied	Neither Satisfied or Unsatisfied	Somewhat Satisfied	Very Satisfied
1	2	3	4	5

Q37	If 1-4, why aren't you satisfied with sanitation within your household/community?

Q38	Where does the food in your household come from?
1	
2	
3	

4	
5	

**Q39**	Which food source is most important?
**Q40**	Which is the second most important?
**Q41**	Which is the third most important?

**Q42**	How often does your household pay for food?			
Never	Rarely	Occasionally	Frequently	All the Time
1	**2**	**3**	4	5
**Q43**	Approximately how much do you spend on food in a month?			
SI$:				

**Q44**	In the past month, how many times were the following true?				
	Never	Rarely	Sometimes	Often	Always
You weren't able to eat the kinds of foods you prefer?	1	2	3	4	5
Your household did not have enough food?	1	2	3	4	5

**Q45**	How many times has your household eaten these food types in the past week?
	Number of Times Eaten in Past Week
Rice (and other grains)	
Flour	
Roots & Tubers (potato, taro etc.,)	
Nuts (cashew nuts, peanuts etc.,)	
Milk & Other Dairy Products (i.e., cheese)	
Canned Meat (Corned Beef, Luncheon Meat/Chicken, Duck Meat)	
Fresh Meat (Chicken wings, pork, beef etc)	
Canned Fish (Taiyo, Besta, Mariko etc)	
Marine Fish/Shellfish (Fish lo sea: Bonito, Reef Fish, Mamula, Snapper etc, Sela lo sea	
Freshwater Fish/Shellfish (Fish or sela lo River/Streams)	
Eggs	
Vegetables & Leaves (slippery cabbage, saladia, fern, kangkong, water crease, two leaf, wild fern, pepper, tomatoe etc)	
Fruits (pawpaw, ripe banana, melon, pineapple, pomolo etc)	
Oil, Fat, Butter	
Sugar or Sweets (lolly, ice cream, ice block, twisties, chewing gum, choco etc)	

**Q46**	In the past year has anything affected your ability to access food?
Yes ☐	
No ☐ (**If NO**, go to Q45)	
**Q47**	**Q48**
**If YES,** what is the name of the problem?	**If YES,** how often have you experienced these problems?

		Very Rarely	Rarely	Occasionally	Frequently	All the Time
A		1	2	3	4	5
B		1	2	3	4	5
C		1	2	3	4	5
D		1	2	3	4	5
E		1	2	3	4	5

**Q49**	What material is the roof of your house made from?
**Q50**	Where do you get this material?
**Q51**	What material are the walls of your house made from?

**Q52**	Where did you get this material from?
**Q53**	What material is the floor of your house made from?
**Q54**	Where did you get this material?

**Q55**	How often do you pay for building materials?			
Never	Rarely	Occasionally	Frequently	All the Time
1	2	3	4	5
**Q56**	Approximately how much do you spend on building materials in a year?			
SI$:				

**Q57**	In the past year have any problems affected your house?
Yes ☐	
No ☐ **(If NO**, go to Q56)	

**Q58**		**Q59**				
**If YES,** what is the name of the problem?		**If YES,** how often have you experienced these problems?				
		Very Rarely	Rarely	Occasionally	Frequently	All the Time
A		1	2	3	4	5
B		1	2	3	4	5

C		1	2	3	4	5
D		1	2	3	4	5

**Q60**	What do you use for lighting?	**Q61**	Where do you get this from?
1			
2			
3			

**Q62**	Which source of lighting is most important?
**Q63**	Which is second most important?
**Q64**	Which is third most important?

**Q65**	How often do you pay for lighting?			
Never	Rarely	Occasionally	Frequently	All the Time
1	2	3	4	5
**Q66**	Approximately how much do you spend on lighting in a month?			
SI$:				

**Q67**	In the past year, were there times when you didn't have access to lighting?		
Yes ☐			
No ☐ (**If NO**, go to Q65)			
**Q68**	**IF YES,** how often did this take place?		
Rarely	Occasionally	Frequently	All the Time
☐	☐	☐	☐

**Q69**	In the past year has anything affected your ability to access lighting?					
Yes ☐						
No ☐ (**If NO**, go to Q68)						
**Q70**		**Q71**				
**If YES,** what is the name of the problem?		**If YES,** how often have you experienced these problems?				
		Very Rarely	Rarely	Occasionally	Frequently	All the Time
A		1	2	3	4	5
B		1	2	3	4	5

C		1	2	3	4	5
D		1	2	3	4	5

**Q72**	What sources of fuel do you use for cooking?	**Q73** Where do you get this fuel from?
1		
2		
3		
4		
5		

**Q74**	Which is the most important source of cooking fuel?
**Q75**	Which is second most important?
**Q76**	Which is third most important?

**Q77** How often do you pay for fuel for cooking?				
Never	Rarely	Occasionally	Frequently	All the Time
1	2	3	4	5
**Q78** Approximately how much do you spend on fuel (gas, firewood etc) in a month?				
SI$:				

**Q79** In the past year, were there time when fuel for cooking was not available?			
Yes ☐			
No ☐ (**If NO**, go to Q75)			
**Q80** **IF YES,** how often did this take place?			
Rarely	Occasionally	Frequently	All the Time
☐	☐	☐	☐

**Q81**	In the past year has anything affected your ability to access fuel for cooking?
Yes ☐	
No ☐ (**If NO**, go to Q78)	

**Q82**	**Q83**				
**If YES,** what is the name of the problem?	**If YES,** how often have you experienced these problems?				
	Very Rarely	Rarely	Occasionally	Frequently	All the Time

A		1	2	3	4	5
B		1	2	3	4	5
C		1	2	3	4	5
D		1	2	3	4	5
E		1	2	3	4	5

**Q84**	How many members of your household suffer from a long term health condition or disability?

**Q85**	Who within your household is suffering from this?

**Q86**	Would you mind sharing what these health conditions or disabilities are? (i.e., physical, mental, sociophysical)

**Q87**	Were there any times in the past year that any member of your household missed their livelihood responsibilities due to being sick, ill or injured?			
None	Few	Some	Many	All the Time
1	2	3	4	5

**Q88**	How often do you pay for medicine or other health related items?			
Never	Rarely	Occasionally	Frequently	All the Time
1	2	3	4	5

**Q89**	Approximately how much do you spend in a year?
SI$:	

**Q90**	In the past year, were there ever times when you or members of your household couldn't access healthcare?
Yes ☐	
No ☐ (**If NO**, go to Q86)	

**Q91**	**IF YES,** how often did this take place?		
Rarely	Occasionally	Frequently	All the Time
☐	☐	☐	☐

**Q92**	**Q93**				
**If YES,** what is the name of the problem?	**If YES,** how often have you experienced these problems?				
	Very Rarely	Rarely	Occasionally	Frequently	All the Time

A		1	2	3	4	5
B		1	2	3	4	5
C		1	2	3	4	5
D		1	2	3	4	5
E		1	2	3	4	5

**Q94**	Does your household have access to any of the following items?	
**Household Asset**	**Items Accessed**	
	**Yes**	**No**
Generator	☐	☐
Fridge/Freezer	☐	☐
Boat/canoe	☐	☐
Boat with motor/OBM	☐	☐
Other transport (specify):	☐	☐
Communication device (i.e., mobile phone or radio)	☐	☐
Internet	☐	☐
Recreational (e.g., musical instruments)	☐	☐
Access to land for gardening	☐	☐
	**Yes**	**No**
Fishing equipment (specify):	☐	☐
Farming equipment (specify):	☐	☐
Livestock (specify):	☐	☐
Other equipment for accessing natural resources (specify):	☐	☐

**Q95**	If you suddenly needed access to food, water, fuel (for cooking & transportation) or healthcare how many people that aren't household members could you turn to who would help?			
No One	1-2 People	3-4 People	>5 People	>10 People
1	2	3	4	5

**Q96**	How much do you agree with the following?				
	Strongly Disagree	Disagree	Neutral	Agree	Strongly Agree
I trust the people in my community	1	2	3	4	5
I am happy with the leadership in my community	1	2	3	4	5
Access to natural resources in my community is fair	1	2	3	4	5
Access to new economic opportunities in my community is fair	1	2	3	4	5

**Q97**	Are you happy with your level of involvement with decisions that are made within your community?			
Strongly Disagree	Disagree	Neutral	Agree	Strongly Agree
1	2	3	4	5

Q98	In the past year did anyone in your household participate in community activities?			
Never	Rarely	Sometimes	Frequently	All the Time
1	3	4	5	5

Q99		Q100
Please list the groups, organisations or associations that your household belongs to?		Does any member of your household have a leadership role in these organisations?
1		
2		
3		

Q101	How much money does your household make on average in one month?			
Less than $250	$250-500	$500-1000	$1000-2000	More than $2000
☐	☐	☐	☐	☐
Q102	How satisfied are you with your ability to purchase the items your household needs to survive (e.g., food, water, healthcare etc.,)?			
Very Dissatisfied	Somewhat Dissatisfied	Neither Satisfied or Dissatisfied	Somewhat Satisfied	Very Satisfied
1	2	3	4	5

Q103	Was your household's income this year higher, lower or about the same as last year?
☐	Higher
☐	Lower
☐	About the same
Q104	**IF Higher or Lower,** why do you believe your income changed?

Q105	Approximately how much money does your household have in savings?				
None	Less than $250	$250-500	$500-1000	$1000-2000	More than $2000
☐	☐	☐	☐	☐	☐

Q106	Approximately how much money does your household spend in one month?				
None	Less than $250	$250-500	$500-1000	$1000-2000	More than $2000
☐	☐	☐	☐	☐	☐

Q107a			Q107b
Do you currently use any of the following ?			**If YES,** what type was this/where did you obtain this from?
Bank Accounts (BSP, ANZ, Bred Bank, POB, Womens Saving Club)	Yes ☐	No ☐	
Pension Accounts (NPF, You Save)	Yes ☐	No ☐	
Loans (BSP, ANZ, Bred Bank, POB, Womens Savings Club, National Finance etc.,)	Yes ☐	No ☐	

Q108	In the past year, has anything affected your access to income?

Yes ☐	
No ☐ (**If NO**, go to Q107)	

**Q109**		**Q110**				
**If YES,** what is the name of the problem?		**If YES,** how often have you experienced these problems?				
		Very Rarely	Rarely	Sometimes	Frequently	All the Time
A		1	2	3	4	5
B		1	2	3	4	5
C		1	2	3	4	5
D		1	2	3	4	5
E		1	2	3	4	5

**Q111**	Have these problems effected your household?				
	Strongly Disagree	Disagree	Neither Agree nor Disagree	Agree	Strongly Agree
Logging	1	2	3	4	5
Changes in Wild Fish Availability	1	2	3	4	5
Sea Level Rise	1	2	3	4	5
Flooding	1	2	3	4	5
Increased Rainfall	1	2	3	4	5
Reduced Rainfall	1	2	3	4	5
Increased Temperature	1	2	3	4	5
Covid-19	1	2	3	4	5
Other (specify below):					
	1	2	3	4	5
	1	2	3	4	5

**Q112**	Does your household have some knowledge of these problems?				
	Strongly Disagree	Disagree	Neither Agree nor Disagree	Agree	Strongly Agree
Logging	1	2	3	4	5
Changes in Wild Fish Availability	1	2	3	4	5
Sea Level Rise	1	2	3	4	5
Flooding	1	2	3	4	5
Increased Rainfall	1	2	3	4	5
Reduced Rainfall	1	2	3	4	5
Increased Temperature	1	2	3	4	5
Covid-19	1	2	3	4	5
Other (specify below):					
	1	2	3	4	5
	1	2	3	4	5

**Q113**	Does your household have ways of **coping** with these problems?

	Strongly Disagree	Disagree	Neither Agree nor Disagree	Agree	Strongly Agree
Logging	1	2	3	4	5
Changes in Wild Fish Availability	1	2	3	4	5
Sea Level Rise	1	2	3	4	5
Flooding	1	2	3	4	5
Increased Rainfall	1	2	3	4	5
Reduced Rainfall	1	2	3	4	5
Increased Temperature	1	2	3	4	5
Covid-19	1	2	3	4	5
Other (specify below):					
	1	2	3	4	5
	1	2	3	4	5

Supplementary Information 3: Risk Interaction Diagrams

Risk interaction diagrams, derived from qualitative data, illustrating interactions among risk components that aided in establishing priority adaptation initiatives for each community.

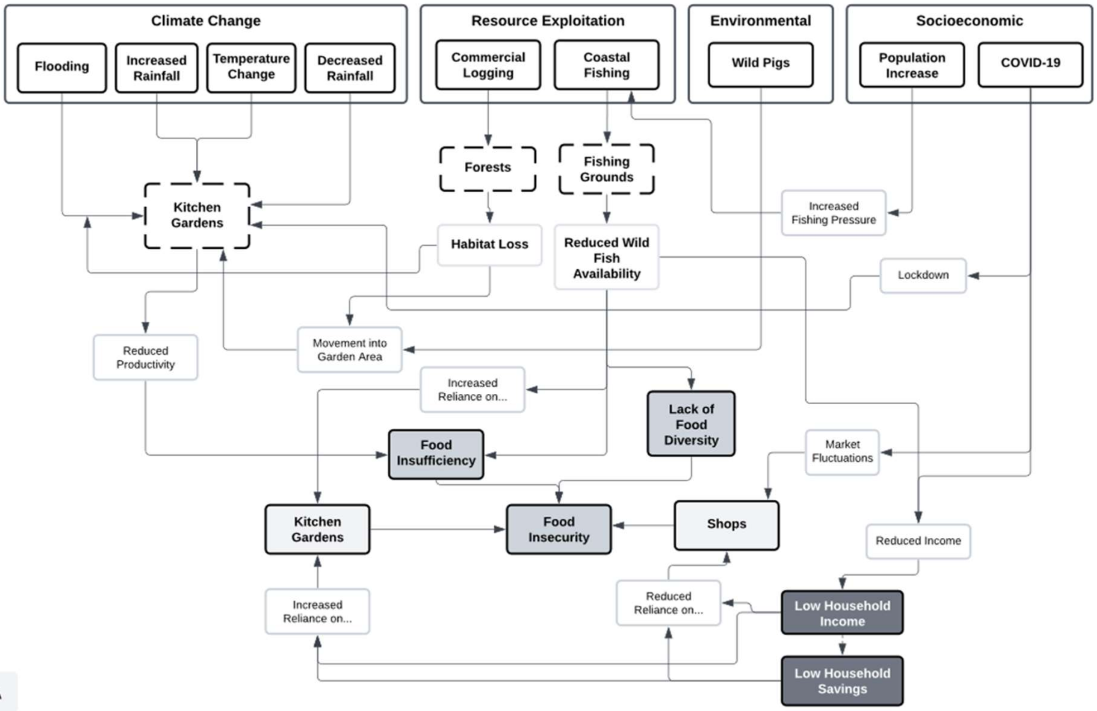

A

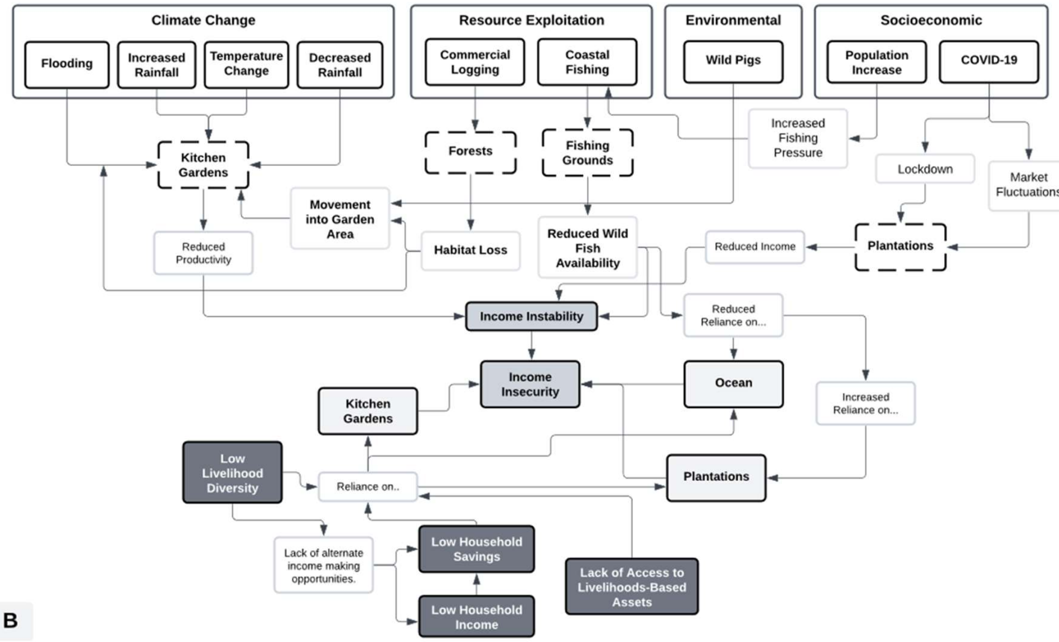

B

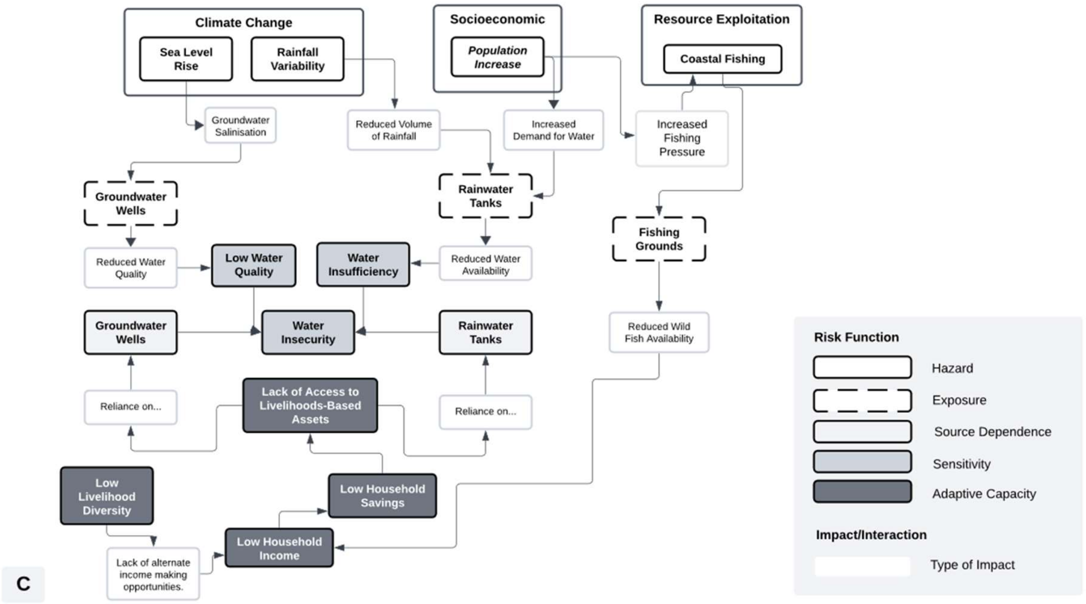

Climate Change
Sea Level Rise
Rainfall Variability
Socioeconomic
Population Increase
Resource Exploitation
Coastal Fishing
Groundwater Salinisation
Reduced Volume of Rainfall
Increased Demand for Water
Increased Fishing Pressure
Groundwater Wells
Rainwater Tanks
Fishing Grounds
Reduced Water Quality
Low Water Quality
Water Insufficiency
Reduced Water Availability
Groundwater Wells
Water Insecurity
Rainwater Tanks
Reduced Wild Fish Availability
Reliance on...
Lack of Access to Livelihoods-Based Assets
Reliance on...
Low Livelihood Diversity
Low Household Savings
Low Household Income
Lack of alternate income making opportunities.
C
Risk Function
Hazard
Exposure
Source Dependence
Sensitivity
Adaptive Capacity
Impact/Interaction
Type of Impact

Supplementary Information 3: Vulnerability Indicator Scores

Table 1: Indicator ranking scores representing composite indicators of adaptive capacity and sensitivity across case study communities. Colours mark the contribution of each composite indicator and component to vulnerability: dark green = very low (VL), green = low (L), yellow = moderate (M), orange = high (H), and red = very high (VH).

Indicator		C1	C2	C3
**Adaptive Capacity**				
**Human Capital**		**L**	**M**	**M**
HC1	Dependency Ratio	M	M	L
HC2	Health Condition	L	L	L
HC3	Access to Healthcare	M	M	M
HC4	Livelihood Diversity Index	M	M	H
HC5	Extent of Coping Strategies	L	M	L
**Financial Capital**		**H**	**H**	**H**
FC1	Household Savings	H	H	H
FC2	Household Income	H	H	M
FC3	Household Expenditure	M	M	M
FC4	Income Satisfaction	M	M	M
FC5	Access to Financial Services	VH	H	M
**Social Capital**		**M**	**M**	**M**
SC1	Social Networks	M	M	M
SC2	Inclusion in Decision Making	M	M	M
SC3	Local Institutional Membership	M	H	H
SC4	Satisfaction with Leadership	M	M	M
SC5	Trust	M	M	M
SC6	Collective Action	M	M	M
SC6	Fair Access to Livelihood Opportunities	M	M	M
**Physical Capital**		**M**	**H**	**H**
PC1	Access to Livelihoods-Based Assets	M	H	H
**Natural Capital**		**M**	**M**	**M**
NC1	Fair Access to Natural Resources	M	M	M
**Sensitivity**				
**Water Security**		**M**	**L**	**H**
WS1	Access to Drinking Water	M	L	VL
WS2	Water Sufficiency	M	L	H
WS3	Water Quality	M	M	H
WS4	Access to Sanitation	VH	H	H
**Food Security**		**M**	**M**	**H**
FS1	Food Sufficiency	M	M	H
FS2	Food Consumption Score	M	M	M
**Housing Security**		**VL**	**L**	**VL**
HS1	Housing Condition	VL	L	VL
**Energy Security**		**L**	**L**	**L**
ES1	Cooking Fuel Sufficiency	L	L	L
ES2	Lighting Sufficiency	M	M	M
**Income Security**		**L**	**H**	**H**
IS1	Income Stability	L	H	H

Contribution to Vulnerability: Very Low (VL) Low (L) Moderate (M) High (H) Very High (VH)

Table 2: Raw indicator scores representing composite indicators of adaptive capacity and sensitivity across case study communities. Colours mark the contribution of each composite indicator and component to vulnerability: dark green = very low, green = low, yellow = moderate, orange = high, and red = very high.

**Vulnerability Component**	**Community**		
	**C1**	**C2**	**C3**
**Adaptive Capacity**	**47.40**	**41.25**	**42.38**
Human Capital	61.19	55.45	53.43
Financial Capital	29.74	23.65	32.10
Social Capital	52.13	49.84	44.30
Physical Capital	52.97	39.14	36.00
Natural Capital	51.98	46.05	46.50
**Sensitivity**	**48.62**	**43.08**	**61.66**
Water Security	56.51	37.91	62.85
Food Security	48.41	49.80	70.30
Housing Security	13.76	22.81	19.33
Energy Security	23.16	35.85	35.62
Income Security	39.68	63.16	67.00
